# Synthesis and Antimycobacterial Assays of Some New Ethambutol Analogs

**DOI:** 10.3390/molecules30030600

**Published:** 2025-01-29

**Authors:** Rana Abdelaziz, Mthandazo Dube, Lea Mann, Adrian Richter, Dina Robaa, Norbert Reiling, Mohammad Abdel-Halim, Peter Imming

**Affiliations:** 1 Institut für Pharmazie, Martin-Luther-Universität Halle-Wittenberg, 06120 Halle (Saale), Germany; rana.abdelaziz@sickkids.ca (R.A.); mthandazo.dube@pharmazie.uni-halle.de (M.D.); lea.mann@pharmazie.uni-halle.de (L.M.); adrian.richter@pharmazie.uni-halle.de (A.R.); dina.robaa@pharmazie.uni-halle.de (D.R.); 2 RG Microbial Interface Biology, Research Center Borstel, Leibniz Lung Center, Parkallee 1–40, 23845 Borstel, Germany; nreiling@fz-borstel.de; 3 German Center for Infection Research (DZIF), Partner Site Hamburg-Lübeck-Borstel-Riems, 23845 Borstel, Germany; 4 Department of Pharmaceutical Chemistry, Faculty of Pharmacy, German University in Cairo, Cairo 11835, Egypt; mohammad.abdel-halim@guc.edu.eg

**Keywords:** mycobacteria, ethambutol (EMB), *Mycobacterium tuberculosis*, non-tuberculous mycobacteria (NTM)

## Abstract

Ethambutol (EMB) is a first-line anti-tuberculosis drug that is also considered in treatment regimens for infections caused by non-tuberculous mycobacteria (NTM). EMB targets the arabinosyl transferases EmbCAB, which are important for the synthesis of cell wall constituents. To further explore and narrow down the structural variability of EMB, we synthesized three series of new EMB analogs. We tested their activity against *Mycobacterium tuberculosis*, *Mycobacterium smegmatis*, *Mycobacterium abscessus* and *Mycobacterium intracellulare*. Only analogs that very closely resembled EMB showed comparable antimycobacterial activity.

## 1. Introduction

Tuberculosis (TB) is caused by *Mycobacterium tuberculosis* (*Mtb*) and is one of the most pervasive respiratory transmitted diseases in history. It is among the top 10 causes of death worldwide. According to a report by the WHO, 10.8 million new TB infections and 1.25 million deaths were reported in 2023 [1]. The continuous selection and emergence of multidrug-resistant (MDR) and extensively drug-resistant (XDR) TB strains raises the need to search for new or modify the already existing anti-tubercular agents [2,3].

Non-tuberculous mycobacteria (NTM) are mycobacteria that are not *Mtb* or *Mycobacterium leprae* and can cause pulmonary and extrapulmonary infections, like TB. They are becoming more common and are recognized as a growing public health concern. The population at highest risk are cystic fibrosis patients, with a 20% prevalence of secondary NTM infections [4]. NTM are resistant to most anti-TB antibiotics, emphasizing the need for new antibiotics that are active against NTM.

**Figure 1:**
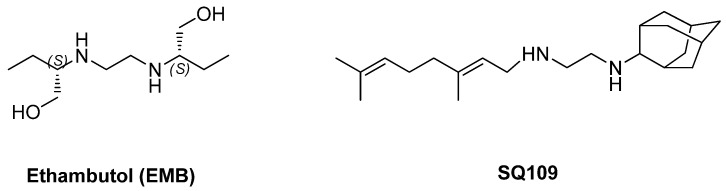
Structures of Ethambutol (EMB) and SQ109.

Ethambutol (EMB, Figure 1) is a first-line anti-TB drug substance that has been a mainstay of TB therapy for about 50 years. It is also considered for treatment regimens against infections with *Mycobacterium abscessus* complex (MABC) and *Mycobacterium avium* complex (MAC). EMB targets Emb proteins identified to be arabinofuranosyl transferases (AraTs). They are trans-membrane (TM) proteins that add a D-arabinofuranose sugar moiety from decaprenyl-phosphoryl-D-arabinose (DPA) to the growing arabinogalactan (AG) and lipoarabinomannan (LAM) [5,6,7]. Many symmetric and asymmetric EMB analogs have been reported [8,9,10,11,12]. Starting from N,N′-diisopropylethylenediamine, varying the length of the alkylene chain and the type of N-alkyl substituent and introducing secondary alkyl alcohol functions, ethambutol was selected from a series of congeners [5,9]. Later, terminal unsaturation was introduced, reportedly leading to in vivo (mice) anti-*Mtb* activity [13]. This finding was disregarded in the literature, which is why we prepared and tested this compound (**7**, v.i.). A small series of congeners of EMB did not have better activity [14]. The largest study by far synthesized more than 60,000 diamine analogs of EMB in pools of ten, deconvoluted approx. 3000 and identified 26 compounds with equal or better activity than EMB [9]. One of them was SQ109 (Figure 1), which was later shown to address several targets different from EMB’s. In summary, the studies showed that EMB allows for very little variation for antimycobacterial activity or, if the structural similarity is lower, other targets are addressed.

**Figure 2:**
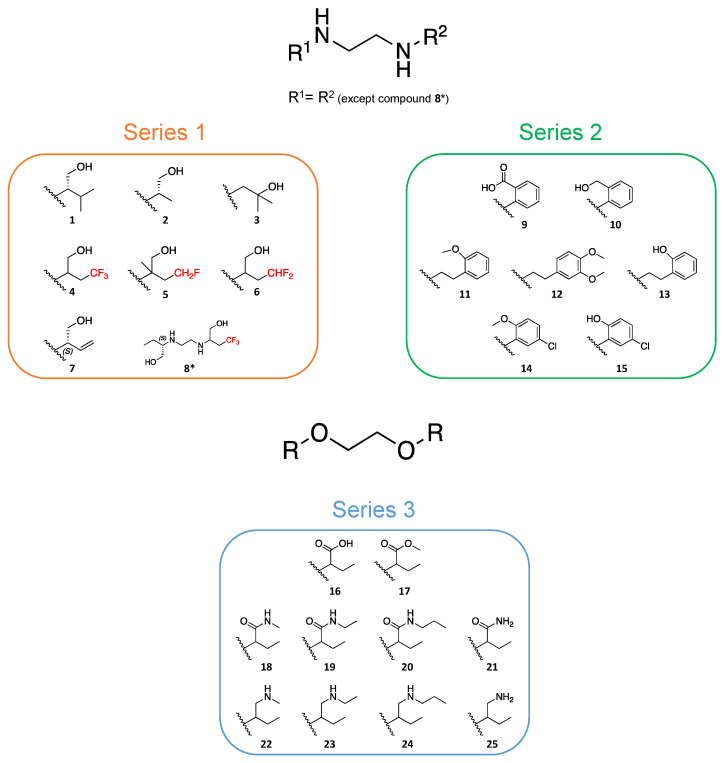
A diagram explaining the modifications made. In Series 1 and 2, the ethylene diamine scaffold of EMB was retained, while in Series 3, the positions of nitrogen and oxygen atoms in EMB were exchanged. * R^1^ ≠ R^2^.

We inspected the chemical space covered already and identified niches that had been disregarded. Our underlying incentive and rationale were twofold each: (1) Incentive: (a) EMB is a mainstay of TB therapy, but resistant mutants have spread; (b) EMB has low activity against therapeutically emerging mycobacteria. (2) Design rationale (Figure 2): Series 1: Explore fluorine substitution in the alkyl side-chain as it will reduce the basicity of the N atom but provide H–F binding to the target. This is of interest because X-ray target analyses suggest that only one N atom is protonated on site [6,7]. Series 2: Explore the possibility of hydrogen bonding through hydroxy, carboxy and ether groups connected to phenyl moieties. This included analogs where hydrogen bonding was prevented or inverted by hydroxy and alkoxy analogs. Series 3: Modify hydrogen binding possibilities by swapping the positions of the N and O atoms. On this basis, we synthesized three series of EMB analogs (Figure 2) and tested their activity against *Mtb*, *Msmeg*, *M. abscessus* and *M. intracellulare*.

## 2. Results and Discussion

**Scheme 1:**
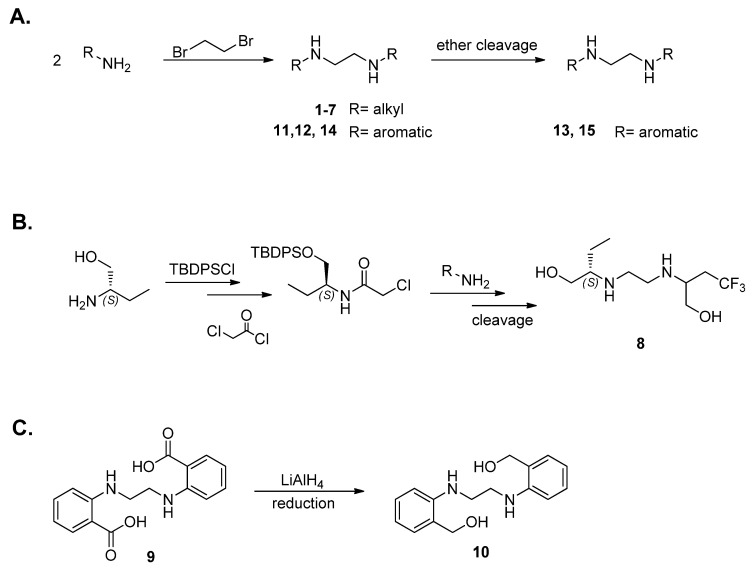
General synthetic scheme for the synthesis of EMB analogs retaining the ethylene diamine bridge (series 1 and 2). (**A**) Synthesis of symmetrical EMB analogs (**1–15**). (**B**) Synthesis of asymmetric EMB analog (**8**). (**C**) Synthesis of Symmetric EMB analog having N-atoms as substituents on aromatic systems (**10**).

The first and second series included analogs **1**–**15,** which retained the ethylene diamine bridge but had modified side chains (Scheme 1). Modifications addressed structural features that had not been considered previously because of the difficult access to the building blocks, particularly oligofluorinated alkyl side chains.

Compounds **1**–**7** were prepared by reacting different amino alcohols with dibromoethane (DBE) (Scheme 1A). The resulting secondary amines, which were more nucleophilic than the primary amine of the starting amino alcohol, reacted with the mono-alkylated bromoethane intermediate to also give side products (see Figure 4). Reaction progress was monitored by APCI-MS and stopped once the side product peaks began appearing; otherwise, the desired compound could not be purified. For compounds **1** and **2**, the amino alcohols used were enantiopure *S*-valinol and *S*-alaninol and the resulting compounds were assumed to be enantiopure as well. To prepare compound **8**, Scheme 1B was used according to a reported protocol [11].

**Figure 3:**
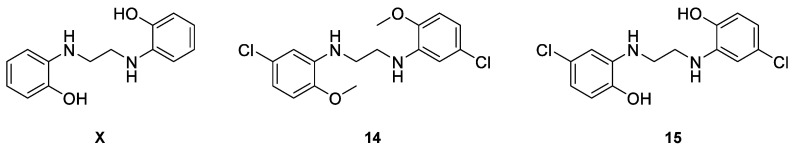
Structures of EMB analogs. X = 2-({2-[(2-hydroxyphenyl)amino]ethyl}amino)phenol [8].

Compound **10** was obtained by the reduction of the carboxylic acid function of *N*,*N*′-ethylenedianthranilic acid **(9)** within one hour using LiAlH_4_ in dry THF under reflux. Yellow crystals of pure **10** were isolated in 67% yield (Scheme 1C). In compounds **11** and **12**, phenylethylamines substituted with methoxy groups at different positions reacted with DBE to obtain the corresponding EMB analogs having the *N*,*N*′-diphenylethyl-1,2-ethylenediamine scaffold (Scheme 1A). The methoxy ether groups in compound **11** were cleaved with aqueous HBr solution to give **13** as a salt [15]. Compounds **14** and **15** are analogs of the previously reported EMB derivative 2-({2-[(2-hydroxyphenyl)amino]ethyl}amino)phenol (Figure 3) [8]. They were synthesized to study the effect of a *p*-Cl substituent and an ether instead of a hydroxyl group on the aromatic ring on activity. Compound **14** was obtained by refluxing dibromoethane (DBE) and 5-chloro-2-methoxyaniline in DMF [16]. The *O*-demethylation of **14** was accomplished using excess boron tribromide and quenching with methanol. Commonly, water or a basic aqueous sodium carbonate solution is used to quench the reaction, giving boric acid and HBr or NaBr as by-products [15]. Methanol, however, gave trimethyl borate and HBr, which were easily removed from the reaction mixture by evaporation under vacuum. The product was obtained as a dibromide salt.

**Scheme 2:**
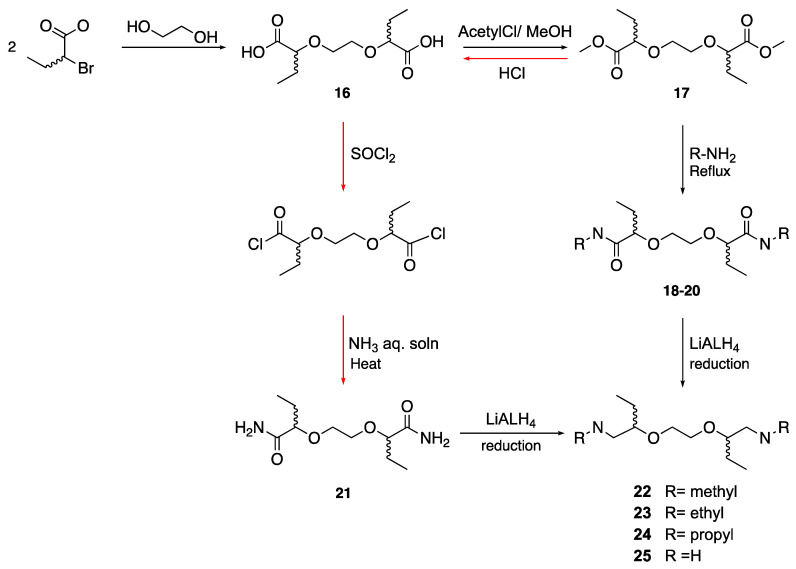
Synthetic scheme for the synthesis of EMB analogs with ethylenedioxy bridge of series 3. The red arrows show the synthetic path for compound **21**.

The third series included analogs **16**–**25** where the nitrogen and oxygen atoms of EMB have exchanged places, a variation that has not been reported (Scheme 2).

The first step was ether formation by bromine substitution from racemic 2-bromobutyric acid with dry ethylene glycol using NaH as a base in dry THF. The product was a di-acid (compound **16**) which was taken to the next step without purification. The second step was the esterification of the di-acid produced. Of note, the diester was a racemic mixture of the *levo*, *dextro* and *meso* isomers. Therefore, in principle, two sets of carbon signals can show in its ^13^C NMR (racemate and *meso*); some, however, overlapped. The purified dimethyl ester (**17**) was taken to the next step, which was amide synthesis. Methyl, ethyl and propyl amines were left to react with compound **17** to afford di-amides **18**–**20**. The speed of the reaction varied among the three amines. We found that it also depended on the water content, which is why a small amount of H_2_O was added with the ethyl and propyl amines. The synthesis of the primary di-amide **21** started with the hydrolysis of the pure dimethyl ester. Then, there was diacid chloride formation via the reaction of the recovered dicarboxylic with thionylchloride. Afterwards, 25% aq. ammonia solution was added to afford **21** (Scheme 2, red arrows). Finally, the four purified amides (**18**–**21**) were reduced using LiAlH_4_ in dioxane under dry conditions. The di-amine produced was obtained by vacuum filtration. APCI-MS and NMR showed that diamines **22**–**25** needed no further purification.

In general, the analogs produced were a mixture of enantiomers and the *meso* diastereoisomer because 2-bromobutyric acid was a racemic mixture. Only with compound **22** was it suspected that, during the purification of the di-amide (compound **18**), one of the stereoisomers was isolated. To determine which stereoisomer of compound **22** was present, a chiral derivatizing reagent (CDA) was added to an NMR sample and the spectrum obtained was analyzed. The CDA used was (*R*)-(+)-α-methoxy-α-trifluoromethylphenylacetic acid ((*R*)-MTPA, Mosher’s acid; see Supplementary Information S1) [17]. (*R*)-MTPA was added in increasing amounts from 0.16 to 2 equivalents (see Supplementary Information S1). The ^1^H NMR did not differ with or without Mosher’s acid; no change in chemical shifts was observed. In addition, ^19^F NMR showed one signal only (no doubling of signals; see Supplementary Information S1). From these findings, we concluded that the *meso*-amine was isolated.

## 3. Biological Evaluation

### 3.1. Screening Against Mycobacteria

The compounds were tested for antimycobacterial activity against *Mtb* (H37Rv), *Msmeg* (*mc*^2^ *155* pTEC27), *M. abscessus* (ATCC19977) and *M. intracellulare* (ATCC 35761).

Screening against *Mtb*: The compounds were divided into two sets; the first set was **1**–**6**, **9**–**12** and **16**–**25**, which were tested at 10 µg/mL (Table 1), and the second set was **4**, **7**, **8**, **11** and **13**–**15**, which were tested at different concentrations to measure their MIC_95_ (Table 2). None of these compounds showed higher potency than EMB (Table 1 and Table 2).

The results of the *Mtb* growth inhibition assays showed that more branching in the side chain of EMB abolished activity in **1** and **3**, which could be due to possible steric clashes in the binding pocket. Altering the side chain length in compound **2** (change α-ethyl to α-methyl) demonstrated minimal impact on activity when compared to EMB, with an inhibition of 63% and 67% at 10 µg/mL, respectively. No inhibition resulted if electron-withdrawing substituents—particularly a fluorine substituent (**4**–**6**)—were near the ethylenediamine N-atoms. In terms of SAR, this indicates that the basicity of the nitrogen atoms must not be reduced.

Compound **7**, the analog with two vinyl groups, showed a fourfold decrease in activity compared to EMB (Table 2), while the asymmetric EMB analog **8** showed no inhibition at the tested concentrations.

Moreover, compounds **9** and **10** did not exhibit activity despite the possibility of Mg^2+^ complexation. This can be attributed to the reduced basicity of the N-atoms (aniline). In addition, in both molecules, the N and O atoms are separated by three rather than two (EMB) carbon atoms, two of them having sp^2^ geometry.

Compound **11,** which differed structurally from EMB (phenoxyethyl side chain), showed lower potency than EMB, with an MIC_95_ of 64 µM compared to 16 µM for EMB. The free hydroxyl group in compound **13** led to an increase in MIC_95_ compared to the methyl ether in **11**. The highest concentration tested was 64 µM (Table 2). At this concentration, compound **13** exhibited only 9% inhibition.

Replacing the secondary alkyl amines with anilines in compounds **14** and **15** led to a decrease in activity (Table 2). Compound **14** showed almost no inhibition at 8 µM. At higher concentrations, compound **14** was insoluble and therefore could not be tested at higher concentrations. Compound **15**, the one with the free hydroxyl groups, showed ~80% inhibition at 64 µM. These data are consistent with the reported SAR of EMB in terms of the ionization of the two nitrogen atoms.

No activity was found when the positions of O and N atoms were swapped (**16**–**25**), highlighting again the importance of the two basic secondary amines of EMB.

### 3.2. Screening Against NTM

Compounds **1**–**6**, **9**–**12** and **16**–**25** were tested against *M. abscessus*; however, they did not show activity up to 100 µM. The second set included compounds **4**, **7**, **8**, **11** and **13**–**15** and was tested against *M. abscessus*, *Msmeg* and *M. intracellulare*. All the compounds showed an MIC_95_ > 100 µM (see Table 2).

### 3.3. In Silico Docking Study

In silico studies of our EMB analogs failed to explain the results of the in vitro growth inhibition assays (Supplementary Information S2). For instance, docking did not predict the loss of activity, as seen when comparing compounds **13** to **11**. In silico studies showed that both could still fit into the EMB binding pocket despite their structural differences from EMB. In addition, the in silico studies alone cannot explain the results of the in vitro growth inhibition assays, as mentioned above, since they do not address the uptake, excretion and metabolism of the compounds by the mycobacteria. Further biological studies, e.g., enzyme inhibition assays, are needed in order to provide better insight into the ability of these compounds to effectively bind into the active site of EmbCAB.

## 4. Experimental

### 4.1. Chemistry

Reagents were obtained from Sigma Aldrich and Enamine were used without further purification. All organic solvents used were of pure analytical grade. Dioxane was dried over molecular sieves (4 Å). THF was dried by refluxing under Argon with Na metal and benzophenone as an indicator for dryness. Column chromatography was carried out using silica-gel 40–60 μm mesh with CHCl_3_:MeOH:NH_4_OH, DCM:MeOH, EtOAc:Heptane, CHCl_3_:MeOH or EtOAc:MeOH:NH_4_OH. Mass spectrometry was performed on APCI-MS (Advion expression^S^ CMS; direct analysis probe), the flow rate used was 10 μL/min, the super soft method was used to avoid fragmentation and the *m*/*z* range was 10 to 1000 with an acquisition speed of 10,000 *m*/*z* units/sec; for ESI-MS (LCQ-Classic, Thermo Finnigan; direct injection), the flow rate was 20 μL/min, the scan range was 50–2000 *m*/*z*, the voltage was 5 KV and the capillary temperature was 220 °C. TLC was stained with KMnO_4_ for the detection of spots. ^1^H-NMR and ^13^C-NMR spectra were recorded at 400 MHz and 101 MHz, respectively, using a Varian VNMS-MR 400 spectrometer. ^1^H shifts are referenced to the residual protonated solvent signal (δ 7.26 for CDCl_3_, δ 3.31, 4.87 for CD_3_OD and δ 2.5 for DMSO-*d_6_*) and ^13^C shifts are referenced to the deuterated solvent signal (δ 77.0 for CDCl_3_, δ 49.0 for CD_3_OD and δ 39.5 for DMSO-*d_6_*). Chemical shifts are given in parts per million (ppm), and all coupling constants (*J*) are given in Hz.

The purities of compounds 9, 12, 18, 19 and 21 were determined by HPLC (Jasco.). Pump: PU-980 intelligent HPLC pump, Detector: UV-975 intelligent UV/VIS detector and Sampler: 851-AS intelligent sampler. HPLC-method: Flow rate 1 mL/min. The solvent system used was acetonitrile: water, 60:40 + 0.05% TFA. The pressure was 67 kg/cm^2^. Peaks were detected at λ = 220 nm.

The purities of compounds 10, 11, 13, 14, 15 and 20 were determined by HPLC Shimadzu (Kyoto, Japan). Pump: two LC-10AD pumps, Detector: SPD-M10A VP PDA detector and Sampler: SIL-HT autosampler. Analytical HPLC: a LiChrospher column RP-18, 5 μm particle size and 10 cm length were used, and the solvent system was MeOH:H_2_O, with 5 to 95% + 0.05% TFA over 30 min. The solvent system used for compound 20 was acetonitrile: water, 5 to 95%, with a flow rate of 1.2 mL/min over 6 min. Peaks were detected at λ = 254 nm.

#### 4.1.1. General Procedures for Synthesis of 2,2′-(Ethylene-1,2-diamino)dibutan-1-ol Analogs (Scheme 1A)

**Figure 4:**
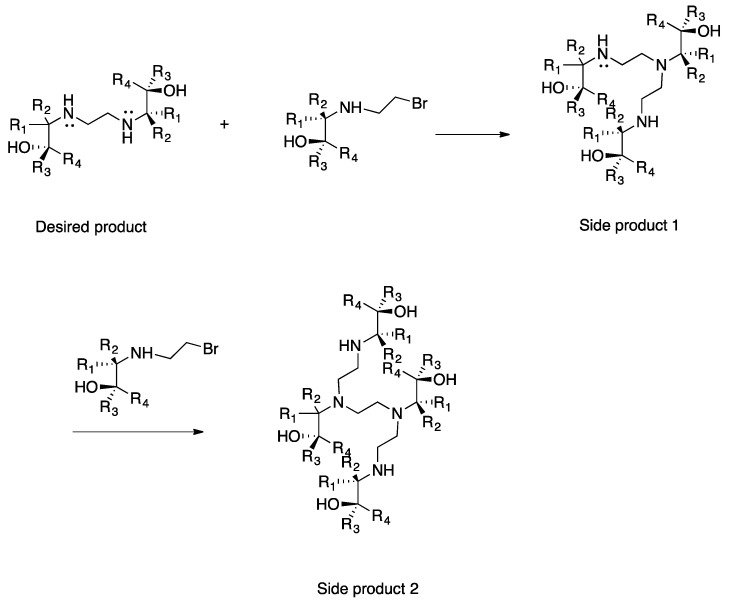
Example of the side products produced from Scheme 1. The desired product included two secondary amines which are more nucleophilic than the primary amine of the starting amino alcohol. As a result, these secondary amines reacted with mono-alkylated bromoethane intermediate, giving side products 1 and 2. The side products were detected by APCI-MS but not isolated.

Dibromoethane (DBE) was added dropwise to the respective amino alcohol in a 25 mL two-necked round bottom flask (1:2 moles). After the addition of DBE was complete, the reaction was left to stir at 80 °C and stopped when side products were detected in the APCI-MS spectra (Figure 4). After cooling down, one of two work-up procedures was carried out.

**Method 1**. The solid or sticky oil produced was dissolved in H_2_O and washed with CHCl_3_. The aqueous phase was basified with 2 equiv. NaOH solution and stirred for 10 min. The free base of the product was extracted with CHCl_3_ (50 mL × 3) and combined organic phases were dried over anhydrous Na_2_SO_4_. The solvent was evaporated and the product was purified by column chromatography (CHCl_3_:MeOH:NH_4_OH, 88:10:2) [18].

**Method 2**. In total, 1.75 M of KOH in ethanol was added and stirring was continued at room temperature for 1 h. The precipitated KBr was then filtered, and ethanol was evaporated under reduced pressure. The resulting oil was purified by recrystallization/precipitation. The oil was dissolved in the least amount of CHCl_3_ and then heptane was added dropwise until the solution became turbid and then left overnight. The formed precipitate was filtered by vacuum filtration [19].

**(2*S*,2′*S*)-2,2′-(ethylene-1,2-diamino)di(3-methylbutan-1-ol) (1):** The synthesis followed the general procedure mentioned above, but the reaction was left for 10 h until all start (*S*-valinol) was consumed and method 1 was used for the work-up. C_12_H_28_N_2_O_2_, yellow oil; yield: 24%; ^1^H NMR (400 MHz, CDCl_3_) δ 3.62 (dd, *J* = 10.8, 3.9 Hz, 2H), 3.38 (dd, *J* = 10.8, 7.9 Hz, 2H), 2.89–2.83 (m, 2H), 2.73–2.63 (m, 6H), 2.39 (ddd, *J* = 7.9, 6.4, 3.9 Hz, 2H), 1.76 (dq, *J* = 13.5, 6.8 Hz, 2H), 0.92 (dd, *J* = 24.0, 6.8 Hz, 12H); ^13^C NMR (101 MHz, CDCl_3_) δ 64.5, 61.7, 47.1, 29.2, 19.3, 18.5; MS (APCI): *m*/*z* 233.3 (M^+^ + 1); R*_f_* = 0.24 (CHCl_3_:MeOH: NH_4_OH, 88:10:2).

**(2*S*,2′*S*)-2,2′-(ethylene-1,2-diamino)di(propan-1-ol) (2):** It followed the general procedure using *S*-alaninol, and the second procedure for work-up was used. C_8_H_20_N_2_O_2_, white powder; yield: 10%; m.p. 92–95 °C; ^1^H NMR (400 MHz, CD_3_OD) δ 3.48 (dd, *J* = 10.9, 4.9 Hz, 2H), 3.39 (dd, *J* = 10.8, 6.8 Hz, 2H), 2.85–2.77 (m, 2H), 2.77–2.70 (m, 2H), 2.70–2.62 (m, 2H), 1.04 (d, *J* = 6.5 Hz, 6H); ^13^C NMR (101 MHz, CD_3_OD) δ 66.4, 55.7, 47.1, 16.7; MS (APCI): *m*/*z* 177.2 (M^+^ + 1); R_f_ = 0.35 (CHCl_3_:MeOH:NH_4_OH, 88:10:2).

**1,1′-(Ethylene-1,2-diamino)di(2-methylpropan-2-ol) (3):** It followed the general procedure using 1-amino-2-methyl-2-propanol, but the reaction was carried out after 1.5 h. The second work-up method was used. Note that this time, crystallization took longer than for other derivatives. C_10_H_24_N_2_O_2_, off-white semi-solid; yield: 16.5%; ^1^H NMR (400 MHz, CDCl_3_) δ 2.78 (s, 4H), 2.54 (s, 4H), 1.17 (s, 12H); ^13^C NMR (101 MHz, CDCl_3_) δ 69.5, 60.0, 49.7, 27.4. MS (APCI): 205.18 *m*/*z* (M^+^ + 1).

**2,2′-(Ethylene-1,2-diamino)di(4,4,4-trifluorobutan-1-ol) (4):** 2-amino-4,4,4-triflourobutan-1-ol.HCl (2.828 mmol) was stirred with DIPEA (2.828 mmol) at 60 °C and then DBE (1.414 mmol) was added dropwise. The mixture was left to stir for 4 h. After cooling down, the second work-up method mentioned in the general procedure was used; however, only CHCl_3_ precipitate started to form and was filtered after being left overnight. C_10_H_18_F_6_N_2_O_2_, white powder; yield: 27%; m.p. 117–122 °C; ^1^H NMR (400 MHz, CD_3_OD) δ 3.63 (dd, *J* = 11.2, 4.7 Hz, 2H), 3.50 (dd, *J* = 11.2, 5.8 Hz, 2H), 2.90 (qd, *J* = 5.9, 4.6 Hz, 2H), 2.74 (s, 4H), 2.45–2.19 (m, 4H); ^13^C NMR (101 MHz, CD_3_OD) δ 126.9 (d, ^1^*J*_CF_ = 275.8 Hz), 62.4, 53.9 (d, ^3^*J*_CF_ = 2.5 Hz), 45.8, 45.7, 34.9 (q, ^2^*J*_CF_ = 27.4 Hz); MS (APCI): *m*/*z* 313.3 (M^+^ + 1); R*_f_* = 0.23 (CHCl_3_:MeOH:NH_4_OH, 88:10:2).

**2,2′-(Ethylene-1,2-diamino)di(4-fluoro-2-methylbutan-1-ol) (5):** The synthesis followed the general procedure using 2-amino-4-fluoro-2-methylbutan-1-ol and the second work-up method was carried out exactly as for compound **4**. C_12_H_26_F_2_N_2_O_2_, white powder; yield: 4.4%; m.p. 115–123 °C; ^1^H NMR (400 MHz, CD_3_OD) δ 4.65 (td, *J* = 6.4, 1.8 Hz, 2H), 4.53 (td, *J* = 6.4, 1.8 Hz, 2H), 3.41 (s, 4H), 2.62 (s, 4H), 1.86 (t, *J* = 6.3 Hz, 2H), 1.80 (t, *J* = 6.3 Hz, 2H), 1.07 (s, 6H); ^13^C NMR (101 MHz, CD_3_OD) δ 80.6 (d, ^1^*J*_CF_ = 162.1 Hz), 65.8, 55.0 (d, ^4^*J*_CF_ = 3.9 Hz), 41.0, 36.1 (dd, ^2^*J*_CF_ = 18.7, 3.0 Hz), 20.4; MS (APCI): *m*/*z* 269.3 (M^+^ + 1); R*_f_* = 0.14 (CHCl_3_:MeOH:NH_4_OH, 88:10:2).

**2,2′-(Ethylene-1,2-diamino)di(4,4-difluorobutan-1-ol) (6):** The compound was synthesized with the same procedure as compound **4** using 2-amino-4,4,-difluorobutan-1-ol but precipitation failed and column chromatography was used instead to purify the product (CHCl_3_:MeOH:NH_4_OH, 78:20:2). C_10_H_20_F_4_N_2_O_2_, beige, semi-solid; yield: 6%; ^1^H NMR (400 MHz, CD_3_OD) δ 6.33–5.82 (m, 2H), 3.70–3.57 (m, 2H), 3.56–3.45 (m, 2H), 2.94–2.83 (m, 2H), 2.86–2.63 (m, 4H), 2.08–1.89 (m, 4H).; ^13^C NMR (101 MHz, CD_3_OD) δ 16.5 (t, *J* = 237.2 Hz), 113.8 (t, ^1^*J*_CF_ = 237.4 Hz), 54.1 (t, ^3^*J*_CF_ = 5.5 Hz), 54.0 (t, ^3^*J*_CF_ = 5.5 Hz), 36.2 (t, ^2^*J*_CF_ = 21.2 Hz), 35.7 (t, ^2^*J*_CF_ = 21.0 Hz); MS(APCI): *m*/*z* 277.1 (M^+^ + 1); R_f_ = 0.35 (CHCl_3_:MeOH:NH_4_OH, 78:20:2).

**(2S,2′S)-2,2′-(ethane-1,2-diylbis(azanediyl))bis(but-3-en-1-ol) (7)** The compound was synthesised by adding dibromoethane (DBE) dropwise to (S)-2-aminobut-3-en1-ol hydrochloride in a 25 mL two-necked round bottom flask (1:2 moles). After the addition of DBE was complete, the reaction was left to stir at 60 °C for 2 h. The sticky oil produced was dissolved in H_2_O and washed with CHCl_3_. The aqueous phase was basified with 2 equiv. NaOH solution and stirred for 30 min. The free base of the product was extracted with CHCl_3_/EtOH (2:1) and combined organic phases were dried over anhydrous Na_2_SO_4_. C_10_H_20_N_2_O_2_, white, semi-solid; yield: 5%; ^1^H NMR (400 MHz, CD_3_OD) δ 5.63 (ddd, *J* = 17.3, 10.3, 8.1 Hz, 2H), 5.24 (ddd, *J* = 17.3, 1.8, 0.9 Hz, 2H), 5.19 (ddd, *J* = 10.4, 1.8, 0.7 Hz, 2H), 3.53 (dd, *J* = 10.9, 4.9 Hz, 2H), 3.44 (dd, *J* = 10.9, 7.6 Hz, 2H), 3.19–3.09 (m, 2H), 2.84–2.71 (m, 2H), 2.67–2.54 (m, 2H), ^13^C NMR (101 MHz, CD_3_OD) 138.7, 118.6, 65.7, 64.7, 47.3; MS(APCI): *m*/*z* 201.158 (M^+^ + 1); R_f_ = 0.33 (DCM:MeOH:NH_4_OH, 79:20:1).

#### 4.1.2. Synthesis of Asymmetric EMB Analog (**8**, Scheme 1B) [11]

**Synthesis of [(2*S*)-2-aminobutoxy](*tert*-butyl)diphenylsilane (1i):** TBDSiCl (1.1 equiv.) was added dropwise to a mixture of imidazole (1.1 equiv.) in DCM at 0 °C. After stirring for 15 min, (*s*)-(*+*), amino alcohol was added. The solution was left to stir for 16 h at room temperature before being poured into a saturated NaHCO_3_ solution. The pH was kept below 12 to avoid the cleavage of the *tert*-butyldiphenylsilane. The organic phase was extracted with DCM (3×) and the collected organic phases were dried over MgSO_4_. DCM was evaporated under vacuum and the crude oil was purified by column chromatography (DCM:1% MeOH + drops of NH_4_OH) to give **1i** oil. C_20_H_29_NOSi, 77% yield, APCI-MS: *m*/*z* 328.3 [M + H]^+^, ^1^H NMR (400 MHz, CDCl_3_) δ 7.72–7.63 (m, 4H), 7.48–7.33 (m, 6H), 3.63 (dd, *J* = 9.9, 4.1 Hz, 1H), 3.43 (dd, *J* = 9.8, 7.1 Hz, 1H), 2.85–2.74 (m, 1H), 1.52–1.36 (m, 2H), 1.07 (s, 9H), 0.89 (t, *J* = 7.5 Hz, 3H).

**Synthesis of *N*-[(2*S*)-1-[(*tert*-butyldiphenylsilyl)oxy]butan-2-yl]-2-chloroacetamide (1j):** Chloroacetyl chloride (1.1 equiv.) was added dropwise to a cooled solution (0 °C) of **1i** and DIPEA (2 equiv.) in DCM. The solution was left to stir overnight at room temperature. Water was added to quench the reaction. The organic phase was extracted with EtOAc (3×) and dried over MgSO_4_. After the evaporation of the solvent, flash chromatography (*tert*-butylmethylether:heptane, 1:2) was used to obtain the pure **1j**. C_22_H_30_ClNO_2_Si, 50% yield, APCI-MS: *m*/*z* 404.2 [M]^+^, ^1^H NMR (400 MHz, CDCl_3_) δ 7.70–7.60 (m, 4H), 7.50–7.33 (m, 6H), 6.96–6.89 (m, 1H), 4.12–3.96 (m, 2H), 3.98–3.85 (m, 1H), 3.70 (d, *J* = 3.3 Hz, 2H), 1.79–1.56 (m, 2H), 1.12–1.05 (m, 9H), 0.95–0.81 (m, 3H).

**Synthesis of *N*-[(2*S*)-1-[(*tert*-butyldiphenylsilyl)oxy]butan-2-yl]-2-[(4,4,4-trifluoro-1-hydroxybutan-2-yl)-amino]acetamide (1k):** 2-amino-4,4,4-triflourobutan-1-ol.HCl (1 equiv.) was added to a cooled solution (0 °C) of **1j** and DIPEA (3 equiv.) in DMF. The reaction was stirred at 70 °C for 14 h before quenching with water. The organic phase was extracted with CHCl_3_ and brine (3×) to get rid of DIPEA and DMF. The collected organic phases were dried over MgSO_4_. Flash chromatography (CHCl_3_:MeOH + 1% NH_4_OH, 0 → 5%) yielded **1k**. C_26_H_37_F_3_N_2_O_3_Si, orange oil, 40% yield, APCI-MS: *m*/*z* 510.3 [M]^+^, ^1^H NMR (400 MHz, CDCl_3_) δ 7.64 (dq, *J* = 8.0, 1.8 Hz, 4H), 7.49–7.33 (m, 6H), 7.15 (d, *J* = 9.3 Hz, 1H), 7.03 (d, *J* = 9.1 Hz, 1H), 3.97–3.85 (m, 1H), 3.76–3.59 (m, 3H), 3.48 (dd, *J* = 11.1, 4.8 Hz, 1H), 3.26 (d, *J* = 1.7 Hz, 2H), 3.00–2.88 (m, 1H), 2.45 (s, 1H), 2.41–2.12 (m, 2H), 1.76–1.46 (m, 2H), 1.07 (s, 9H), 0.86 (td, *J* = 7.5, 2.7 Hz, 3H).

**Synthesis of 4,4,4-trifluoro-2-[(2-{[(2S)-1-hydroxybutan-2-yl]amino}ethyl)amino]butan-1-ol (8)** A two-necked round-bottom flask was dried and flushed with Argon. LiAlH_4_ (6 equiv.) was suspended in dry dioxane and cooled to 0 °C. **1k** (1 equiv.) was diluted in a small amount of dry dioxane before being added dropwise to the suspension. The mixture was then left to stir under Reflux (100 °C) overnight. For the workup, the reaction flask was cooled to 0 °C and H_2_O (H_2_O:LiAlH_4_, 1:1), NaOH 15% aq. Solution (NaOH:LiAlH_4_, 1:1) and H_2_O (H_2_O:LiAlH_4_, 3:1) were cautiously added and left to stir overnight at room temperature. The solids were removed by vacuum filtration and the filtrate was concentrated under reduced pressure. The crude oil was dissolved in CHCl_3_ and then drops of heptane were added until **8** crushed out as a white precipitate. It was filtered and left overnight in the desiccator to dry. C_10_H_21_F_3_N_2_O_2_, white solid, 9% yield, m.p. 118–120 °C, calculated monoisotopic mass *m*/*z* 258.15551, ESI-HRMS: *m*/*z* 281.1448 [M + Na]^+^, ^1^H NMR (400 MHz, CD_3_OD) δ 3.71–3.60 (m, 2H), 3.53 (dd, *J* = 11.2, 5.7 Hz, 1H), 3.45 (dd, *J* = 11.0, 6.6 Hz, 1H), 2.94 (m, 1H), 2.82–2.68 (m, 4H), 2.52 (tt, *J* = 6.8, 5.0 Hz, 1H), 2.46–2.25 (m, 2H), 1.63–1.39 (m, 2H), 0.97 (t, *J* = 7.5 Hz, 3H). ^13^C NMR (101 MHz, CD_3_OD) δ 126.9 (q, CF_3_, ^1^*J*_CF_ = 275.9 Hz), 62.6, 62.4 (d, ^4^*J*_CF_ = 1.2 Hz), 60.6–60.5 (m), 53.9 (q, ^3^*J*_CF_ = 2.4 Hz), 46.0, 45.8, 34.9 (q, ^2^*J*_CF_ = 27.2 Hz), 23.4, 9.3. The HPLC purity was 89% and was not further purified since it did not show any in vitro activity.

#### 4.1.3. Synthesis of Symmetric EMB Analogs Having N-Atoms as Substituents on Aromatic Systems (Scheme 1A,C)

***N*,*N*′-Ethylenedianthranilic acid (9) was commercially available.** C_16_H_16_N_2_O_4_, white powder; m.p.228 °C [20]; ^1^H NMR (400 MHz, DMSO-*d*_6_) δ 7.78 (dd, *J* = 8.0, 1.7 Hz, 2H), 7.35 (ddd, *J* = 8.7, 7.1, 1.8 Hz, 2H), 6.82 (dd, *J* = 8.6, 1.0 Hz, 2H), 6.56 (ddd, *J* = 8.0, 7.1, 1.0 Hz, 2H), 3.45 (s, 4H); ^13^C NMR (101 MHz, DMSO-*d*_6_) δ 170.3, 151.2, 134.9, 132.2, 114.8, 111.7, 110.6, 41.7; MS (APCI): *m*/*z* 301.1 (M^+^ + 1).

**2,2′-(ethylene-1,2-diamino)-di(phenylmethanol) (10):** In a two-necked 25 mL round bottom flask under Argon, LiAlH_4_ (1.99 mmol, 6 equiv.) was suspended in 10 mL dry THF. *N*,*N*′-ethylenedianthranilic acid (0.33 mmol) was added and the mixture was heated under reflux for 1 h. The completion of the reaction was concluded using TLC (EtOAc:Heptane, 1:1). The reaction was cooled to 0 °C with an ice/water bath. Then, it was treated cautiously in sequence with H_2_O (0.1 mL), NaOH 15% aq. solution (0.1 mL) and H_2_O (0.3 mL) and left to stir overnight. THF was decanted and the residue was filtered under vacuum and washed with EtOAc and CHCl_3_. Organic layers were combined and dried over anhydrous Na_2_SO_4_. When evaporating some of the solvent under reduced pressure, crystals started to appear, and the flask was removed and left to crystallize for 3 days. Crystals of pure 10 were then filtered. C_16_H_20_N_2_O_2_, yellow crystals; yield: 67%; m.p. 123–124 °C; ^1^H NMR (400 MHz, CD_3_OD) δ 7.19–7.10 (m, 2H), 7.07 (dd, *J* = 7.4, 1.6 Hz, 2H), 6.74 (dd, *J* = 8.1, 1.1 Hz, 2H), 6.63 (td, *J* = 7.4, 1.1 Hz, 2H), 4.56 (s, 4H), 3.44 (s, 4H), 3.34 (s, 4H); ^13^C NMR (101 MHz, CD_3_OD) δ 148.5, 130.0, 129.86, 126.8, 117.9, 111.8, 64.1, 43.8; MS (APCI): *m*/*z* 273.2 (M^+^ + 1); R*_f_* = 0.22 (EtOAc:Heptane, 1:1).

***N*^1^,*N*^2^-di(2-methoxyphenethyl)ethane-1,2-diamine dihydrochloride (11):** The compound was synthesized following the general procedure mentioned above, but the reaction was left for 16 h at 100 °C until all 2-methoxyphenethylamine was consumed and method one was used for the work-up, except the solid obtained after drying was dissolved in EtOH, neutralized with dil. HCl and then recrystallized using diethylether; C_20_H_30_N_2_O_2_Cl_2_, white solid; yield: 13%; m.p. 236–241 °C; ^1^H NMR (400 MHz, DMSO-*d*_6_) δ 9.55 (s, 4H), 7.26 (t, *J* = 7.8 Hz, 2H), 6.98–6.75 (m, 6H), 3.76 (s, 6H), 3.33 (s, 4H), 3.27–3.16 (m, 4H), 2.98 (dd, *J* = 9.8, 6.5 Hz, 4H); ^13^C NMR (126 MHz, DMSO-*d*_6_) δ 159.9, 138.9, 130.2, 121.3, 114.8, 112.8, 55.5, 48.1, 43.2, 32.0; MS (ESI): *m*/*z* 329.4 (M^+^ + 1); R*_f_* = 0.59 (MeOH:CHCl_3_ 3% + 1% TFA).

***N*^1^,*N*^2^-bis(3,4-dimethoxyphenethyl)ethane-1,2-diamine dihydrochloride (12):** The synthesis was the same as for compound **11**, using 3,4-Dimethoxyphenylethylamine as the start. C_22_H_34_N_2_O_4_Cl_2_, white solid; yield 9%; m.p. 226–230 °C; ^1^H NMR (400 MHz, DMSO-*d*_6_) δ 9.44 (s, 4H), 6.91–6.85 (m, 4H), 6.77 (dd, *J* = 8.2, 2.0 Hz, 2H), 3.76 (s, 6H), 3.73 (s, 6H), 3.34 (s, 4H), 3.18 (s, 4H), 2.90 (m, 4H); ^13^C NMR (101 MHz, DMSO-*d*_6_) δ 148.8, 147.8, 129.2, 120.6, 112.6, 112.1, 55.6, 47.9, 42.8, 31.1. MS (ESI): *m*/*z* 389.3 (M^+^ + 1); R*_f_* = 0.66 (CHCl_3_:3%MeOH:TEA 0.5%).

***N*^1^,*N*^2^-di((2-hydroxyphenyl)ethyl)ethane-1,2-diamine dihydrobromide (13):** An aqueous solution HBr (48%, excess) was added carefully to **11**. The mixture was left to stir for 3 h at 130 °C and then left overnight at room temperature. The reaction was monitored with thin-layer chromatography (TLC, CHCl_3_:MeOH 2%:NH_4_OH 0.05%). Water was evaporated under vacuum and the solid was purified by preparative HPLC. C_18_H_26_N_2_O_2_^2+^, grey solid, 23% yield, melts with decomposition at 250 °C, calculated monoisotopic mass *m*/*z* 302.19943, ESI-HRMS: *m*/*z* 301.1912 (C_18_H_25_N_2_O_2_) [M + H]^+^, ^1^H NMR (400 MHz, Deuterium Oxide) δ 7.33 (t, *J* = 7.8 Hz, 2H), 6.95–6.84 (m, 6H), 3.46 (s, 4H), 3.42 (t, *J* = 7.3 Hz, 4H), 3.03 (t, *J* = 7.3 Hz, 4H); ^13^C NMR (101 MHz, Deuterium Oxide) δ 155.9, 137.9, 130.5, 120.9, 115.7, 114.3, 49.0, 43.1, 31.6.

***N^1^*,*N^2^*-bis(5-chloro-2-methoxyphenyl)ethane-1,2-diamine (14):** Under an inert atmosphere, dibromoethane (DBE, 6.34 mmole) was added dropwise to a solution of 5-chloro-2-methoxyaniline (4 equiv.) in dry DMF. CaCO_3_ (1.66 equiv.) was added and the mixture was stirred vigorously at 100 °C for 2 h [16]. After the removal of DMF under vacuum, the mixture was extracted three times with DCM and dried over Na_2_SO_4_. The crude oil was purified by column chromatography (*tert*-butylmethylether:heptane, 1:2 +1% TFA). C_16_H_18_Cl_2_N_2_O_2,_ White solid, 15% yield, m.p. 135–136 °C, calculated monoisotopic mass *m*/*z* 340.07453, ESI-HRMS: *m*/*z* 341.0814 [M + H]^+^, ^1^H NMR (500 MHz, CDCl_3_) δ 6.64 (d, *J* = 8.3 Hz, 2H), 6.63 (dd, *J* = 8.3, 2.2 Hz, 2H), 6.58 (d, *J* = 2.2 Hz, 2H), 4.77–4.72 (m, 2H), 3.81 (s, 6H), 3.40 (s, 4H). ^13^C NMR (126 MHz, CDCl_3_) δ 145.5, 138.9, 126.4, 115.9, 110.2, 109.8, 55.6, 42.7.

***N^1^*,*N^2^*-bis(5-chloro-2-hydroxyphenyl)ethane-1,2-diamine dihydrobromide (15):** To cleave the ether, 5 equiv. of BBr_3_ (1M in DCM) were slowly added to **14** and left to stir at 0 °C—room temperature for 5 h [21]. The reaction progress was monitored by the disappearance of the start signal in the APCI-MS. MeOH was added to quench the reaction, and after the evaporation of the solvent, the pure **15** was obtained without further purification. C_14_H_16_Cl_2_N_2_O_2_^2+^_,_ brown solid, 78% yield, m.p. 135–136 °C, calculated monoisotopic mass *m*/*z* 312.04323, ESI-HRMS: *m*/*z* 311.0366 [M − H]^−^,392.9605 [M + Br]^−^, 313.0506 [M + H]^+^, 392.9588 [M + Br]^+^. ^1^H NMR (400 MHz, CD_3_OD) δ 7.18 (d, *J* = 2.5 Hz, 2H), 7.08 (dd, *J* = 8.7, 2.5 Hz, 2H), 6.90 (d, *J* = 8.6 Hz, 2H), 3.65 (s, 4H); ^13^C NMR (101 MHz, CD_3_OD) δ 148.9, 129.1, 127.4, 125.9, 120.4, 117.8, 45.8.

#### 4.1.4. General Procedures for Synthesis of 2,2′-(Ethylene-1,2-dioxo)dibutan-1-amine Analogs (Scheme 2)

**Synthesis of 2,2′-(ethylene-1,2-dioxo)dibutanoic acid. Method A:** In a three-necked 250 mL round bottom flask and under Argon, NaH (4 equiv.) 60% in paraffin was washed with heptane (2 × 20 mL) to remove paraffin. After decanting the heptane, dry THF was added then ethylene glycol (1 equiv.) was added gradually, and the mixture was left to stir at room temperature for 30 min. The reaction flask was put in an ice bath (0 °C) and 2-bromobutyric acid (2 equiv.) was added dropwise after being diluted in dry THF. After the addition was complete, the reaction was left to reflux overnight. The reaction was quenched by the addition of distilled water. The acidification to pH~2 was accomplished by the dropwise addition of conc. HCl. The aqueous phase was extracted with ethyl acetate (3×), and then the combined organic fractions were dried over anhydrous Na_2_SO_4_. Ethyl acetate was removed under reduced pressure, leaving the yellow oil. The crude oil was taken to the next step without further purification [22].

**Method B.** In a three-necked 250 mL round bottom flask under Argon, NaH (80 mmol) 60% in paraffin was suspended in 10 mL dry DMF. Ethylene glycol (10 mmol) was added, and the mixture was left at room temperature to stir for 30 min. The reaction flask was put in an ice bath (0 °C) and 2-bromobutyric acid (20 mmol) was added dropwise. After the addition was complete, the reaction was left at 60 °C. The reaction was quenched by the addition of distilled water. The acidification went to pH~2 by the dropwise addition of conc. HCl. The aqueous phase was extracted with ethyl acetate (×3), and then the combined organic fractions were passed over anhydrous Na_2_SO_4_. Ethyl acetate was evaporated. The oil obtained was purified by column chromatography (wash with 400 mL hexane to remove paraffin, DCM:MeOH 5%).

**2,2′-(Ethylene-1,2-dioxo)-dibutanoic acid (16):** C_10_H_18_O_6_, brown crystals; yield: 21%; melts with decomposition between 180–230 °C; ^1^H NMR (400 MHz, CD_3_OD) δ 3.86 (s, 4H), 3.77–3.65 (m, 2H), 1.84 (tt, *J* = 14.3, 7.1 Hz, 4H), 1.01–0.90 (m, 6H); ^13^C NMR (101 MHz, CD_3_OD) δ 178.2, 83.1, 68.5, 24.7, 8.4; MS(ESI): *m*/*z* 233.032 (M^+^ − 1); R*_f_* = 0.31 (DCM:MeOH, 1:1).

**Synthesis of dimethyl 2,2′-(ethylene-1,2-dioxo)dibutanoate:** A solution of MeOH (20 mL) and acetyl chloride (1 mL) at 0 °C was left to stir for 15 min. before adding the diaicid (**16**) (2 mmol) portionwise. The mixture was heated to reflux overnight. MeOH was evaporated under vacuum, followed by the addition of water and saturated NaHCO_3_ to neutralize the aq. phase. This was followed by extraction with ethyl acetate (50 mL × 3); the combined organic layers were dried over anhydrous Na_2_SO_4_ and the solvent was evaporated under reduced pressure to give an oily product. The resulting dimethylester was purified by column chromatography (EtOAc:Heptane, 1:1).

**Dimethyl 2,2′-(ethylene-1,2-dioxo)dibutanoate (17):** C_10_H_22_O_6,_ oil; yield: 24%; ^1^H NMR (400 MHz, CDCl_3_) δ 3.90 (dt, *J* = 7.3, 5.4 Hz, 2H), 3.81–3.73 (m, 2H), 3.72 (s, 6H), 3.61–3.53 (m, 2H), 1.83–1.66 (m, 4H), 1.00–0.90 (m, 6H); ^13^C NMR (101 MHz, CDCl_3_) δ 173.2, 173.2, 80.6, 80.4, 77.3, 77.0, 76.7, 69.9, 69.7, 51.7, 51.7, 26.2, 26.1, 9.6, 9.5; MS (ESI): *m*/*z* 285.79 (M^+^ + 23); MS (APCI): *m*/*z* 263.1 (M^+^ + 1); R*_f_* = 0.54 (EtOAc:Heptane, 1:1).

**Synthesis of 2,2′-(ethylene-1,2-dioxo)dibutanamide derivatives:** In a 10 mL round bottom flask, the purified ester was added gradually to aqueous alkyl amine solution (4 equiv.). The mixture was left to stir at 70 °C (reflux) until the reaction was complete. For methyl amine (40% solution), the reaction took 3 h, while for ethylamine (70% solution), it took 3 days, and for propyl amine, (98% solution) it took 7 days. After the reaction was complete, the product was concentrated under reduced pressure and left in the desiccator overnight to remove water. The diamide was purified by column chromatography (EtOAc:MeOH:NH_4_OH, 94.5:5:0.5 **18**, CHCl_3_:MeOH:NH_4_OH, 89:10:1 **19**, CHCl_3_:MeOH, 95:5 **20**).

**2,2′-(Ethylene-1,2-dioxo)di(*N*-methylbutanamide) (18)**: C_12_H_24_N_2_O_4_, oil; yield: 57%; ^1^H NMR (400 MHz, CDCl_3_) δ 6.81 (d, *J* = 65.5 Hz, 2H), 3.77 (ddd, *J* = 6.9, 5.5, 4.2 Hz, 2H), 3.73–3.68 (m, 2H), 3.68–3.59 (m, 2H), 2.84 (d, *J* = 4.9 Hz, 6H), 1.97–1.82 (m, 2H), 1.77–1.64 (m, 2H), 0.96 (td, *J* = 7.4, 3.9 Hz, 6H); ^13^C NMR (101 MHz, CDCl_3_) δ 172.9, 172.8, 82.1, 69.8, 69.6, 26.3, 25.9, 25.6, 9.3, 9.2; MS (APCI): *m*/*z* 261.1 (M^+^ + 1); R*_f_* = 0.12 (EtOAc:MeOH:NH_4_OH, 94.5:5:0.5).

**2,2′-(Ethylene-1,2-dioxo)di(*N*-ethylbutanamide) (19):** C_14_H_28_N_2_O_4_, oil; yield: 18%; ^1^H NMR (400 MHz, CDCl_3_) δ 6.76 (d, *J* = 55.2 Hz, 2H), 3.75–3.63 (m, 4H), 3.64–3.59 (m, 2H), 3.36–3.17 (m, 4H), 1.94–1.78 (m, 2H), 1.75–1.55 (m, 2H), 1.12 (t, *J* = 7.3 Hz, 6H), 0.93 (td, *J* = 7.4, 4.0 Hz, 6H); ^13^C NMR (101 MHz, CDCl_3_) δ 172.0, 172.0, 82.0, 82.0, 69.7, 69.5, 33.8, 33.7, 26.3, 25.9, 15.0, 14.9, 9.4, 9.2; MS (APCI): *m*/*z* 289.3 (M^+^ + 1); R*_f_* = 0.41 (CHCl_3_:MeOH:NH_4_OH, 97:2:1).

**2,2′-(Ethylene-1,2-dioxo)di(*N*-propylbutanamide) (20):** C_16_H_32_N_2_O_4_, oil; yield: 59%; ^1^H NMR (400 MHz, CDCl_3_) δ 6.79 (d, *J* = 60.5 Hz, 2H), 3.76–3.73 (m, 2H), 3.71–3.57 (m, 4H), 3.29–3.13 (m, 4H), 1.93–1.81 (m, 2H), 1.69 (tt, *J* = 14.6, 7.3 Hz, 2H), 1.58–1.47 (m, 4H), 1.02–0.87 (m, 12H); ^13^C NMR (101 MHz, CDCl_3_) δ 172.2, 172.17, 82.1, 69.8, 69.6, 40.7, 40.6, 26.4, 26.0, 22.94, 22.90, 11.4, 11.3, 9.5, 9.3; MS (APCI): *m*/*z* 317.4 (M^+^ + 1); R*_f_* = 0.4 (CHCl_3_:MeOH, 95:5).

**Synthesis of 2,2′-(ethylene-1,2-dioxo)dibutanamide**: The diester was hydrolyzed to the dicarboxylic acid again by adding 1N HCl (2 equiv.) and refluxed overnight. The mixture was concentrated under reduced pressure and then extracted with diethylether (20 mL × 5) and dried over anhydrous Na_2_SO_4_. The solvent was evaporated and the obtained oil was left in the desiccator overnight. Afterwards, SOCl_2_ (2 equiv.) was added together with a few small crystals of NaCl and left to reflux overnight. Excess SOCl_2_ was removed by adding toluene, followed by evaporation under reduced pressure. The final step was adding NH_3_ (25% aq. solution) and heating to 70 °C. The reaction was monitored with APCI until completion. The resulted diamide was purified by column chromatography (CHCl_3_:MeOH:NH_4_OH, 89:10:1).

**2,2′-(Ethylene-1,2-dioxo)dibutanamide (21):** C_10_H_20_N_2_O_4_, white powder; yield: 59.45%; m.p. 119–120 °C; ^1^H NMR (400 MHz, CDCl_3_) δ 6.73 (d, *J* = 38.6 Hz, 2H), 5.96 (s, 2H), 3.77–3.71 (m, 4H), 3.71–3.65 (m, 2H), 1.85 (ddt, *J* = 12.1, 4.6, 2.8 Hz, 2H), 1.73 (dq, *J* = 14.3, 7.1 Hz, 2H), 0.97 (td, *J* = 7.4, 3.5 Hz, 6H); ^13^C NMR (101 MHz, CDCl_3_) δ 175.41, 175.40, 82.01, 82.0, 69.8, 69.7, 26.0, 25.7, 9.3, 9.2; MS (APCI): *m*/*z* 233. 2 (M^+^ + 1); R*_f_* = 0.35 (CHCl_3_:MeOH:NH_4_OH, 89:10:1).

**Synthesis of 2,2′-(ethylene-1,2-dioxo)di(butan-1-amine) derivatives:** In a two-necked 50 mL round bottom flask under Argon, LiAlH_4_ (6 equiv.) was suspended in 5 mL dioxane. The flask was cooled to 0 °C in an ice/water bath, and the amide derivative was first dissolved in 5 mL dioxane and then added dropwise while stirring. After the complete addition, the reaction was left to reflux at 100 °C overnight. The reaction was cooled to 0 °C and treated cautiously in sequence with H_2_O (H_2_O:LiAlH_4_, 1:1), NaOH 15% aq. solution (NaOH:LiAlH_4_, 1:1), H_2_O (H_2_O:LiAlH_4_, 3:1) and left to stir overnight. The solids were removed by vacuum filtration over a glass filter and washed with dioxane and EtOAc. The filtrate was concentrated under reduced pressure and left overnight in the desiccator. No further purification of the diamine produced was required.

**2,2′-(Ethylene-1,2-dioxo)di(N-methylbutan-1-amine) (22):** C_12_H_28_N_2_O_2_, yellow oil; yield: 85.48%; ^1^H NMR (400 MHz, CDCl_3_) δ 3.70–3.61 (m, 2H), 3.56–3.48 (m, 2H), 3.36 (p, *J* = 6.1 Hz, 2H), 2.56 (d, *J* = 5.6 Hz, 4H), 2.40 (s, 6H), 1.96 (s, 2H), 1.59–1.41 (m, 4H), 0.87 (t, *J* = 7.5 Hz, 6H); ^13^C NMR (101 MHz, CDCl_3_) δ 80.6, 68.96, 68.9, 55.1, 55.06, 36.5, 25.1, 25.05, 9.6; MS (APCI): *m*/*z* 233.3 (M^+^ + 1).

**2,2′-(Ethylene-1,2-dioxo)di(N-ethylbutan-1-amine) (23):** C_14_H_32_N_2_O_2,_ yellow oil; yield: 69.7%; ^1^H NMR (400 MHz, CDCl_3_) δ 3.72–3.65 (m, 2H), 3.62–3.52 (m, 2H), 3.46–3.35 (m, 2H), 2.64 (dddd, *J* = 8.9, 7.4, 5.6, 1.9 Hz, 8H), 1.62–1.39 (m, 4H), 1.18–1.05 (m, 6H), 0.94–0.80 (m, 6H); ^13^C NMR (101 MHz, CDCl_3_) δ 80.5, 80.45, 68.8, 68.7, 52.64, 52.62, 44.03, 44.00, 25.0, 24.94, 14.9, 9.48, 9.46.; MS (APCI): *m*/*z* 261.3 (M^+^ + 1).

**2,2′-(Ethylene-1,2-dioxo)di(N-propylbutan-1-amine) (24):** C_16_H_36_N_2_O_2,_ yellow oil; yield: 69.7%; ^1^H NMR (400 MHz, CDCl_3_) δ 3.71–3.64 (m, 2H), 3.62–3.54 (m, 2H), 3.42 (dddd, *J* = 13.5, 12.0, 6.9, 3.9 Hz, 2H), 2.68–2.59 (m, 4H), 2.58–2.48 (m, 4H), 1.65–1.37 (m, 8H), 0.92–0.87 (m, 12H); ^13^C NMR (101 MHz, CDCl_3_) δ 80.8, 69.1, 69.0, 53.11, 53.1, 52.18, 52.16, 25.3, 25.25, 23.2, 11.9, 9.8, 9.78.; MS (APCI): *m*/*z* 289.4 (M^+^ + 1).

**2,2′-(Ethylene-1,2-dioxo)di(butan-1-amine) (25):** C_10_H_24_N_2_O_2_, yellow oil; yield: 98.3%; ^1^H NMR (500 MHz, CDCl_3_) δ 3.69–3.60 (m, 2H), 3.61–3.52 (m, 2H), 3.19 (tt, *J* = 8.6, 4.5 Hz, 2H), 2.75 (dd, *J* = 13.2, 3.7 Hz, 2H), 2.64 (ddd, *J* = 13.2, 6.9, 1.4 Hz, 2H), 1.61–1.49 (m, 2H), 1.51–1.37 (m, 2H), 0.97–0.76 (m, 6H); ^13^C NMR (126 MHz, CDCl_3_) δ 82.9, 82.8, 68.9, 44.6, 24.5, 9.7; MS(APCI): *m*/*z* 205.2 (M^+^ + 1).

### 4.2. Microbiology

#### 4.2.1. The First Set of Test Compounds **1**–**6, 9**–**12, 16**–**25**

**Protocol of *Mtb* assay: Bacteria.** Green fluorescent protein (GFP)-expressing *M. tuberculosis* bacteria (H37Rv strain) [2,23] were grown in 7H9 complete medium (cat# 271310; BD Difco; Becton Dickinson, Heidelberg, Germany) supplemented with oleic acid-albumin-dextrose-catalase (OADC, 10%; BD, # 212351), 0.2% glycerol and 0.05% Tween 80. At the mid-log phase (OD_600_ = 0.4), the cultures were harvested and frozen in aliquots at −80 °C.

**Compound preparation and assay procedure:** Starting from a compound stock solution in DMSO of 10 mg/mL, 1 μL of each compound was diluted with 800 μL 7H9 test medium. Then, 80 μL of each concentration was added in triplicate to a black 96 well plate with a clear bottom (Corning Incorporated, Corning, NY, USA). The aliquots of *Mtb* bacteria needed were thawed in a heating block at 37 °C and centrifuged for 10 min at 3700× *g* in a swingout rotor. Supernatants were discarded and bacteria were thoroughly resuspended in 7H9 medium (10% OADC) in the absence of glycerol and Tween80 by use of a syringe and a 26-gauge syringe needle to reach a concentration of 2 × 10^6^/20 μL. In total, 20 μL of the bacterial suspension was added to the wells containing an 80 μL well in the absence (Ctrl) or presence of the compounds. Each plate was prepared with rifampicin (National Reference Center, Borstel, Germany) as the reference compound (dilutions in water). The plates were sealed with an air-permeable membrane (Porvair Sciences, Norderstedt, Germany) and cultured under mild agitation (Heidolph, Schwabach, Germany), not stacked, at 37 °C in an incubator. Bacterial growth was determined at day 7 by measuring the relative fluorescence intensity using a microplate reader (Synergy 2, BioTek, Fisher Scientific GmbH, Schwerte, Germany) [23].

**Protocol of *M. abscessus* assay: Single point in vitro activity determination.** The compounds were screened in 96-well flat-bottom Corning Costar plates at a concentration of 100 µg/mL in MHII medium supplemented with 0.05% tyloxapol (final volume of 100 µL). The concentration of the inoculum was 5 × 10^5^ cells/mL (OD_600_ 0.1 = 1 × 10^8^ CFU/mL). The starting inoculum was diluted from a preculture at the mid-log phase (OD_600_ 0.3 to 0.7). The plates were sealed with parafilm, put in a container with moist tissue and incubated for 6 days at 37 °C. Each plate had four negative controls (1% DMSO) and positive controls (175 µM gentamicin). After incubation, the plates were monitored by the determination of OD_600_ values (plate reader: Victor^2^ 1420 Multilabel Counter PerkinElmer, Rodgau, Germany. The assay was performed in duplicate. A growth inhibition of >90% is considered activity.

#### 4.2.2. Second Set of Test Compounds **4, 7, 8, 11, 13**–**15** [5]

**Protocol of *Mtb* assay:** *M. tuberculosis* (*Mtb*) strain H37Rv (ATCC 25618) carrying a mCherry-expressing plasmid (pCherry10) [24] was cultured in 7H9 complete medium (BD Difco; Becton Dickinson) supplemented with oleic acid-albumin-dextrose-catalase (OADC, 10%; BD), 0.2% glycerol and 0.05% Tween80, as previously described. At the mid-log phase (OD_600_ = 0.4), the cultures were harvested and frozen in aliquots at −80 °C [25]. Frozen aliquots of mCherry-*Mtb* H37Rv were thawed and centrifuged (3700× *g*, 10 min). Supernatants were discarded and bacteria were thoroughly resuspended in 7H9 medium (10% OADC) in the absence of glycerol and Tween80 by use of a syringe and a 26-gauge syringe needle. The bacterial suspension was passed in and out of the syringe about 10 times. Compounds were tested in twofold dilutions starting at 64 µM in triplicates (2 × 10^6^ bacteria, volume 100 µL) for their anti-tubercular activity using 96-well flat clear bottom black polystyrene microplates (Corning^®^ CellBIND^®^, New York, NY, USA). Each plate was prepared with rifampicin (National Reference Center, Borstel, Germany) as a reference compound. The plates were sealed with an air-permeable membrane (Porvair Sciences, Wrexham, UK) in a 37 °C incubator with mild agitation (TiMix5, Edmund Bühler GmbH, Bodelshausen, Germany), as previously described [26]. Bacterial growth was measured as relative light units (RLU) from the fluorescence intensity obtained at an excitation wavelength of 575 nm and an emission wavelength of 635 nm (microplate reader, BioTek Synegy 2, Fisher Scientific GmbH, Schwerte, Germany) after 7 days. The obtained values were normalized to RLU values of the solvent control (DMSO)-treated bacteria set to 100%), and MIC_95_ of each compound was determined. MIC_95_ was defined as the minimum concentration of the compound required to achieve a reduction in fluorescence by 95%. Obtained MIC values were validated by a visual Resazurin microtiter assay (REMA) [27] by adding 30 μL of 0,02% Resazurin (Cayman Chemical, Ann Arbor, USA) solution to each well followed by another 20 h of culture without agitation.

**Protocol of M. intacellulare, M. smegmatis and M. abscessus assays** [5].

**Bacterial cells and culture media.** *M. intracellulare* ATCC 35761, *M.abscessus* ATCC19977 or *M. smegmatis mc*^2^ *155* pTEC27, expressing RFP tdTomato, were used for the activity assays. Stocks of the bacteria grown in complete 7H9 broth were stored in approximately 15% glycerol at −80 °C. Using an inoculation loop, the bacteria were spread on 7H10 plates (containing 400 mg/mL hygromycin) and grown in an incubator at 37 °C.

The bacteria were grown in 7H9 broth supplemented with 10% ADS (0.8% sodium chloride, 5.0% bovine serum albumin and 2.0% dextrose), 0.05% Tween 80 and hygromycin (400 mg/mL). The culture volume was 10 mL in a 50 mL Falcon tube. The tubes were covered to protect the photosensitive hygromycin and shaken in an incubator at 37 °C.

**MIC determination**. MICs were determined against *M. intracellulare* ATCC 35761, *M. abscessus* ATCC19977 or *M. smegmatis* mc^2^ 155 pTEC27, the broth microdilution method. 96-well flat bottom tissue culture plates (Sarstedt AG & Co, Nümbrecht, Germany, 83.3924.500) were used. In the fourth well of each row, two times the desired highest concentration of the tested compound was added in 7H9 medium. Each compound was diluted twofold in a nine-point serial dilution. The concentration of the starting inoculum was 5 × 10^5^ cells ml^−1^. The starting inoculum was diluted from a preculture at the mid-log phase (OD_600_ 0.2 to 0.8, mid-log phase) and an OD_600_ of 0.1 was correlated to 1 × 10^8^ CFU mL^−1^. The plates were sealed with parafilm, placed in a container with moist tissue and incubated at 37 °C. The incubation period for *M. intracellulare* is five days, and the incubation period for *M. smegmatis* and *M. abscessus* is three days. Row three of the 96-well plate included eight negative controls (1% dimethyl sulfoxid [DMSO]) and row two contained eight positive controls (100 µM amikacin). After incubation, the plates were monitored by RFP measurement (λ*_ex_* = 544 nm λ*_em_* = 590 nm) (BMG labtech Fluostar Optima). The assay was performed in duplicate and the results were validated by OD measurement.

Every assay plate contained eight wells with 1% DMSO as the negative control, which corresponds to 100% bacterial growth, and eight wells with the respective inhibitor as a positive control, in which 100% inhibition of bacterial growth was reached. Controls were used to monitor the assay quality through the determination of the Z′ score. The Z′ factor was calculated as follows:

$$Z^{\prime }=1-\frac{3\left({\mathrm{SD}}_{\mathrm{inhibitor}}+{\mathrm{SD}}_{\mathrm{DMSO}}\right)}{M_{\mathrm{inhibitor}}-M_{\mathrm{DMSO}}}$$

(SD = standard deviation, M = mean).

The percentage of growth inhibition was calculated by the following equation:

$$\%\mathrm{growth}\;\mathrm{inhibition}=-100\%\times \frac{{\mathrm{RFP}}_{\mathrm{signal}}\left(\mathrm{sample}\right)-{\mathrm{RFP}}_{\mathrm{signal}}\left(\mathrm{DMSO}\right)}{{\mathrm{RFP}}_{\mathrm{signal}}\left(\mathrm{DMSO}\right)-{\mathrm{RFP}}_{\mathrm{signal}}\left(\mathrm{inhibitor}\right)}$$

## 5. Conclusions

The in vitro growth inhibition assays of the EMB analogs reported here corroborate that even small modifications to EMB will either reduce or abolish activity. Or, they will lead to a shift in target, as was reported for SQ109, which was designed as an EMB analog, albeit its constitution differs substantially from the parent compound, EMB [10,11]. Neither EMB nor any of the analogs inhibited the growth of the notoriously robust *M. abscessus* up to a concentration of 100 µM. The fluorination of EMB’s alkyl chains abolished activity, perhaps because of the diminished electron density of the nitrogen atoms, leading to weak complexation properties, which are thought to cause anti-*Mtb* activity. Analogous reasoning is proposed to explain why swapping the nitrogen and oxygen atoms to give the “inverted” EMB analogs **22**–**25** did not retain activity. In contrast, compound **2** with only methyl instead of ethyl side chains matched EMB activity, suggesting that the binding site is small, the binding mainly necessitating proper positioning of the nitrogen atoms. Binding and cocrystallization studies of EMB and the analogs will support or refute this hypothesis. EMB analogs with phenylalkyl substituents had anti-*Mtb* activity and are leads for analogs with lower MICs. However, it needs to be investigated if they indeed address EMB target(s).

## Figures and Tables

**Table 1:** Percentage growth inhibition of Mtb for compounds **1**–**6**, **9**–**12** and **16**–**25** at a test concentration of 10 µg/mL.

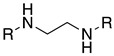			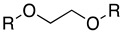		
Code	R	% Inhibition 10 µg/mL	Code	R	% Inhibition 10 µg/mL
**1**	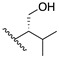	n.i.	**16**	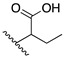	n.i.
**2**	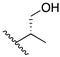	63	**17**	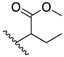	n.i.
**3**	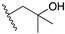	n.i.	**18**	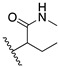	n.i.
**4**	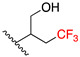	n.i.	**19**	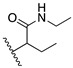	n.i.
**5**	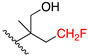	n.i.	**20**	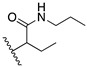	n.i.
**6**	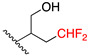	n.i.	**21**	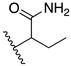	n.i.
**9**	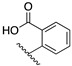	n.i.	**22**	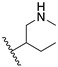	n.i.
**10**	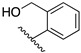	n.i.	**23**	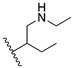	n.i.
**11**	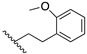	42	**24**	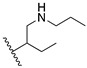	n.i.
**12**	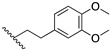	n.i.	**25**	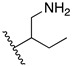	n.i.
**EMB**	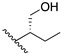	67			

n.i. = no inhibition.

**Table 2:** MIC_90_ * (µM) of the tested EMB analogs against Mtb, Msmeg, M. abscessus and M. intracellulare.

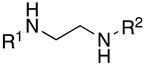						
Code	R^1^ = R^2^		Mtb (MIC_95_) *	Msmeg	M. abscessus	M. intracellulare
**4**	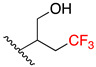		>>64	>100	>100	>100
**7**	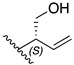		64	n.d.	>100	n.d.
**8**	**R^1^** 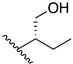	**R^2^** 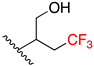	>>64	>100	>100	>100
**11**	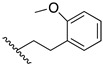		64	>100	>100	>100
**13**	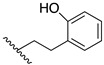		>>64	>100	>100	>100
**14**	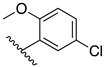		>8 µM **	>100	n.d.	>100
**15**	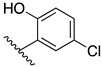		>64	>100	>100	>100
**EMB**	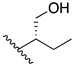		16	1.6	150	4.7

* MIC_90_ or MIC_95_ = minimum concentration that caused >90% (or >95%) growth reduction and indicates an average of two independent measurements. ** 8 µM was the highest tested concentration due to the compound’s insolubility at higher concentrations.

## Data Availability

The data that support the findings of this study are available in the Supplementary Material of this article.

## Supplementary Materials

The following supporting information can be downloaded at: https://www.mdpi.com/article/10.3390/molecules30030600/s1, Supporting Information S1 and S2.
