# Supplementary material for: Synthesis and Antimycobacterial Assays of Some New Ethambutol Analogs
Source: Molecules. 2025 Jan 29;30(3):600. doi: 10.3390/molecules30030600 (PMC11820526; doi:10.3390/molecules30030600)
Supplement: Supplementary file 1 [file molecules-30-00600-s001.zip › molecules-3390504-supplementary.pdf]

Supplementary Information 1

Synthesis and antimycobacterial assays of some new ethambutol analogues

Rana Abdelaziz[1*], Mthandazo Dube[1], Lea Mann[1], Adrian Richter[1], Dina Robaa[1], Norbert Reiling[2,3], Mohammad Abdel-Halim[4], ✉[1]Peter Imming[1]

[1]Institut für Pharmazie, Martin-Luther-Universität Halle-Wittenberg, Halle (Saale), Germany.
[2]RG Microbial Interface Biology, Research Center Borstel, Leibniz Lung Center, Parkallee 1–40, Borstel 23845, Germany.

[3]German Center for Infection Research (DZIF), Partner Site Hamburg-Lübeck-Borstel-Riems, Borstel, Germany.

[4]Department of Pharmaceutical Chemistry, Faculty of Pharmacy, German University in Cairo, Cairo 11835, Egypt.

*Current address: Molecular Medicine Program, The Hospital for Sick Children, Toronto, Canada.

Contents

NMR **3**

Compound 1 3

Compound 2 5

Compound 3 6

Compound 4 7

Compound 5 9

Compound 6 10

Compound 7 12

Compound 8 13

Compound 9 14

Compound 10 15

Compound 11 16

Compound 12 18

Compound 13 19

Compound 14 21

Compound 15 23

Compound 16 25

Compound 17 26

Compound 18 28

✉ Corresponding author: peter.imming@pharmazie.uni-halle.de

Compound 19 30
Compound 20 31
Compound 21 33
Compound 22 34
Compound 23 36
Compound 24 38
Compound 25 39
HRMS 41
Compound 8 41
Compound 13 41
Compound 14 42
Compound 15 42
HPLC 44
Compound 9 44
Compound 10 45
Compound 11 45
Compound 12 46
Compound 13 46
Compound 14 47
Compound 15 47
Compound 18 48
Compound 19 49
Compound 20 50
Compound 21 51

Figures

Figure S1. ^{13}C NMR of Compound **17**.......... 27
Figure S2. (A) Mosher's acid (MTPA). **(B)** ^{1}H NMR of **22** with 0.16 equivalents MTPA. **(C)** ^{1}H NMR of **22** with 2 equivalents of MTPA. **(D)** ^{19}F NMR of **22** with 2 equivalents MTPA.......... 35

NMR

Compound 1

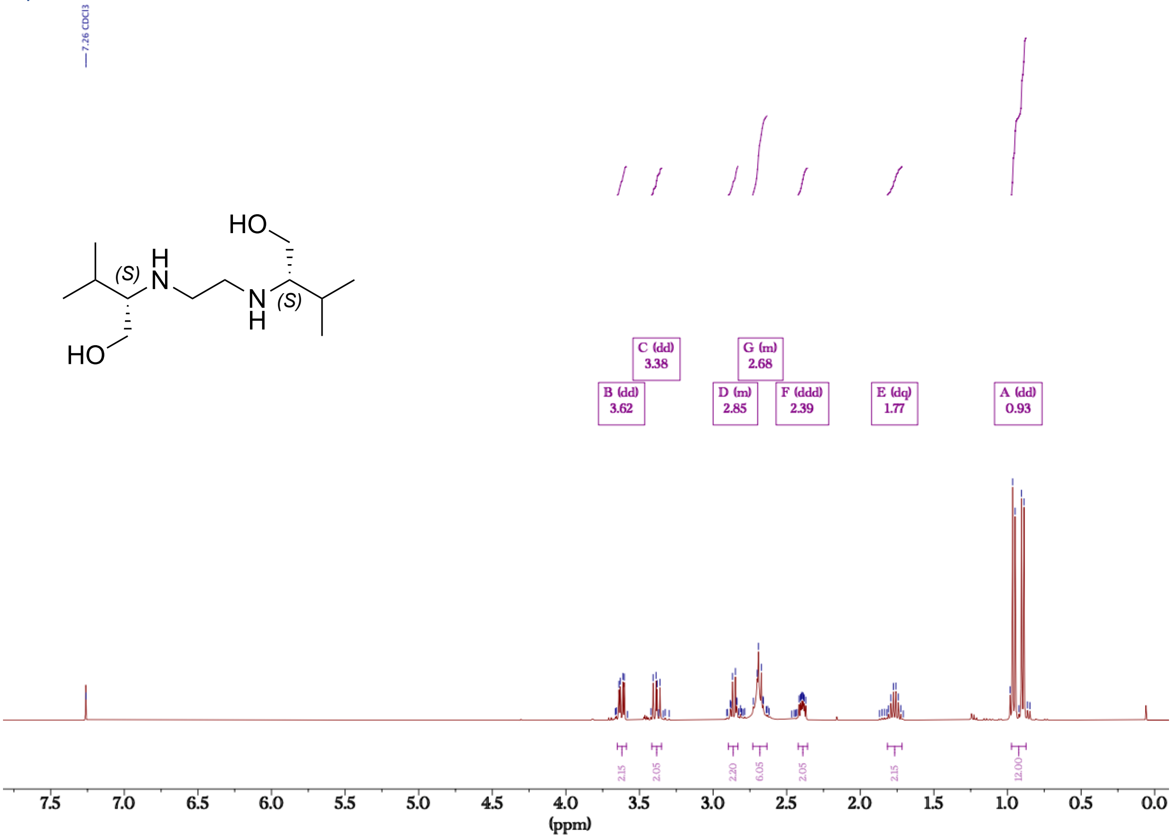

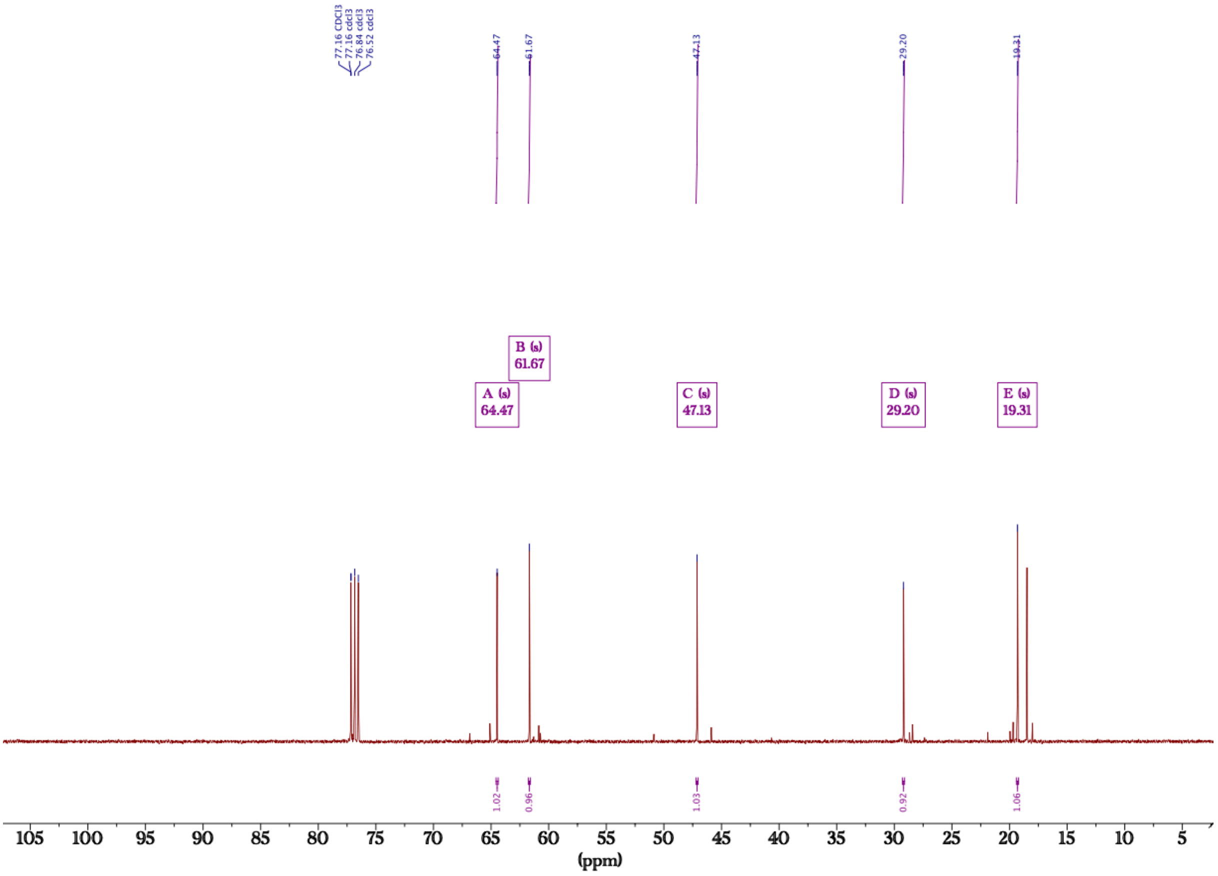
77.16 CDCl3
77.16 cdcl3
76.84 cdcl3
76.52 cdcl3
64.47
61.67
47.13
29.20
19.31
B (s)
61.67
A (s)
64.47
C (s)
47.13
D (s)
29.20
E (s)
19.31
1.02
0.96
1.03
0.92
1.06
105
100
95
90
85
80
75
70
65
60
55
50
45
40
35
30
25
20
15
10
5
(ppm)

Compound 2

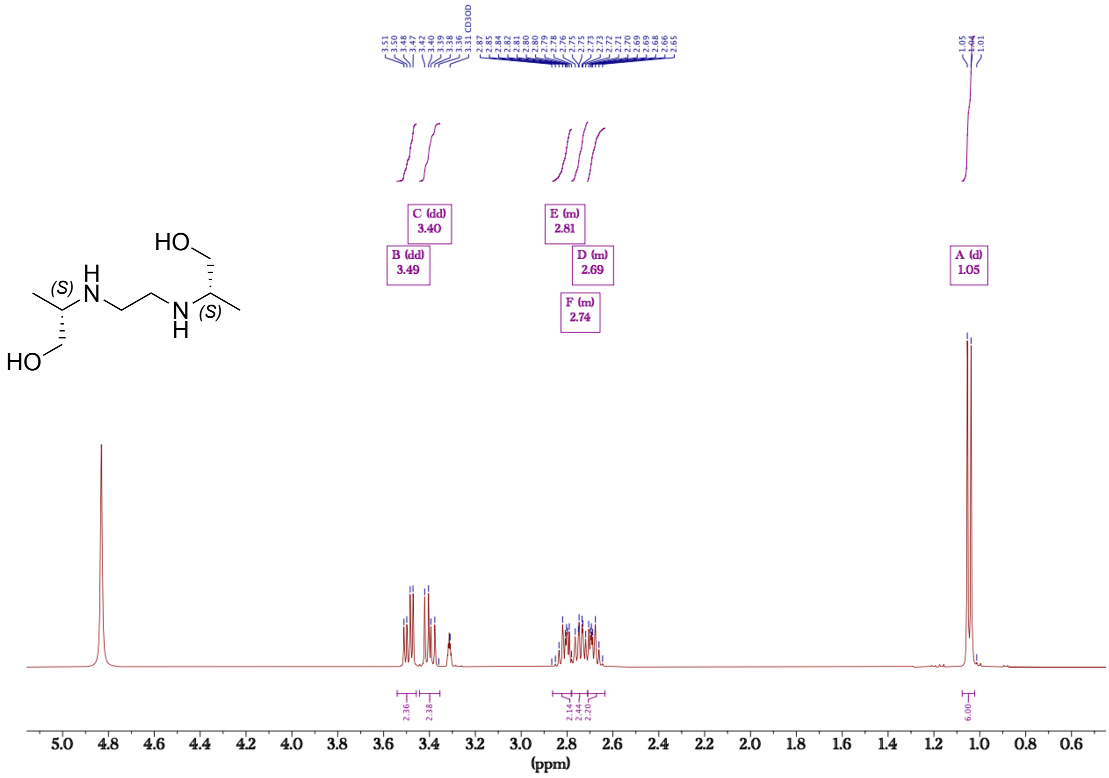
HO
(S)
H
N
N
H
(S)
HO
CD3OD
C (dd)
3.40
B (dd)
3.49
E (m)
2.81
D (m)
2.69
F (m)
2.74
A (d)
1.05
2.36
2.38
2.14
2.44
2.20
6.00
(ppm)

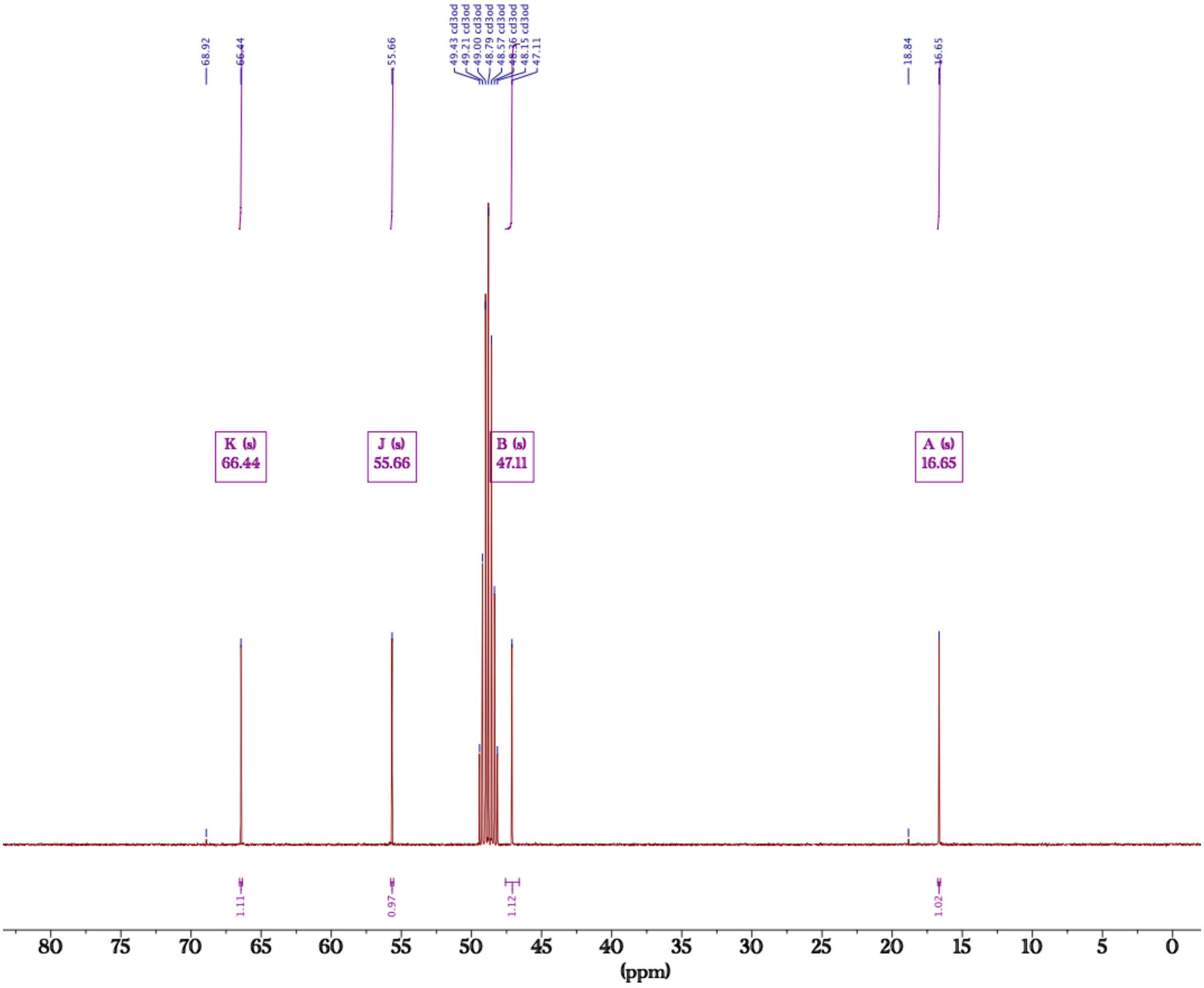
68.92
66.44
55.66
49.43 cd3od
49.21 cd3od
49.00 cd3od
48.79 cd3od
48.57 cd3od
48.36 cd3od
48.15 cd3od
47.11
18.84
16.65
K (s)
66.44
J (s)
55.66
B (s)
47.11
A (s)
16.65
1.11
0.97
1.12
1.02
(ppm)

Compound 3

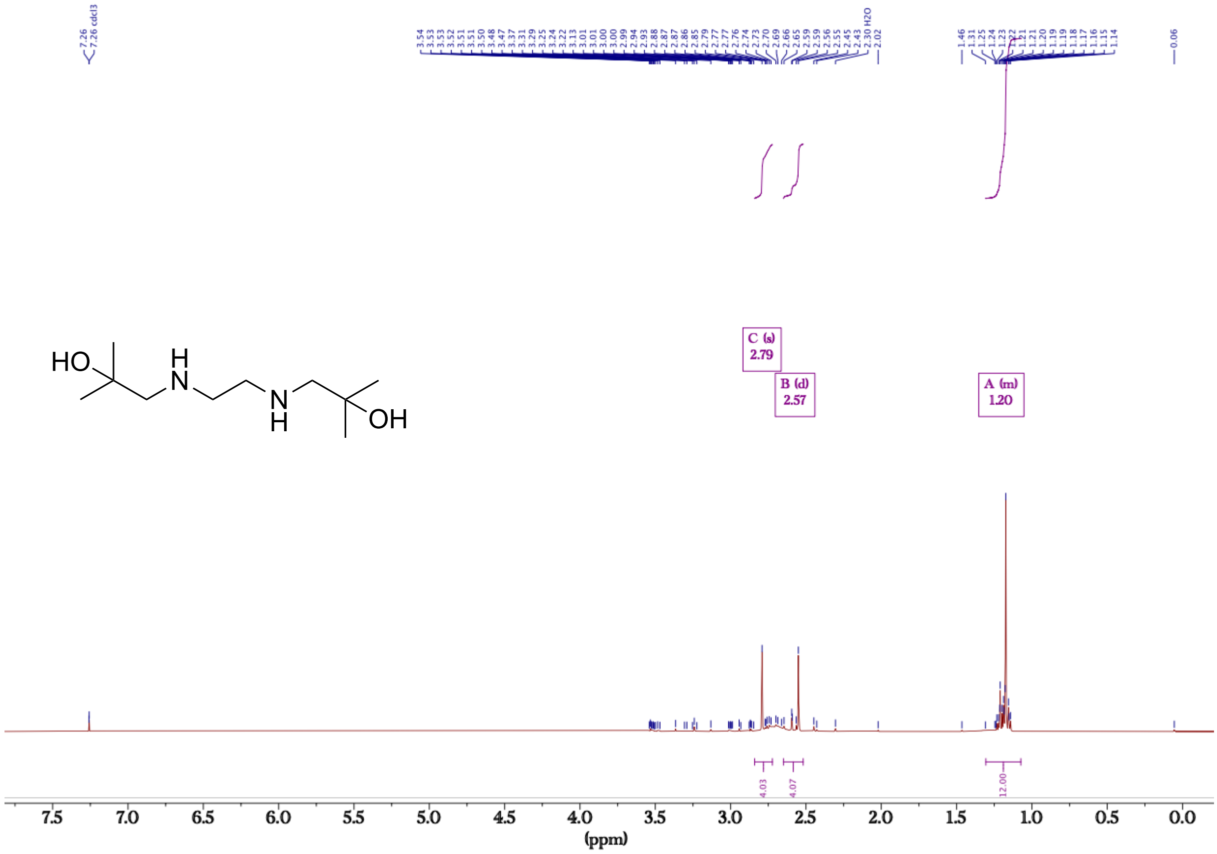

HO
H
N
N
H
OH
7.26
7.26 cdcl3
2.30 H2O
2.02
0.06
C (s)
2.79
B (d)
2.57
A (m)
1.20
4.03
4.07
12.00
(ppm)

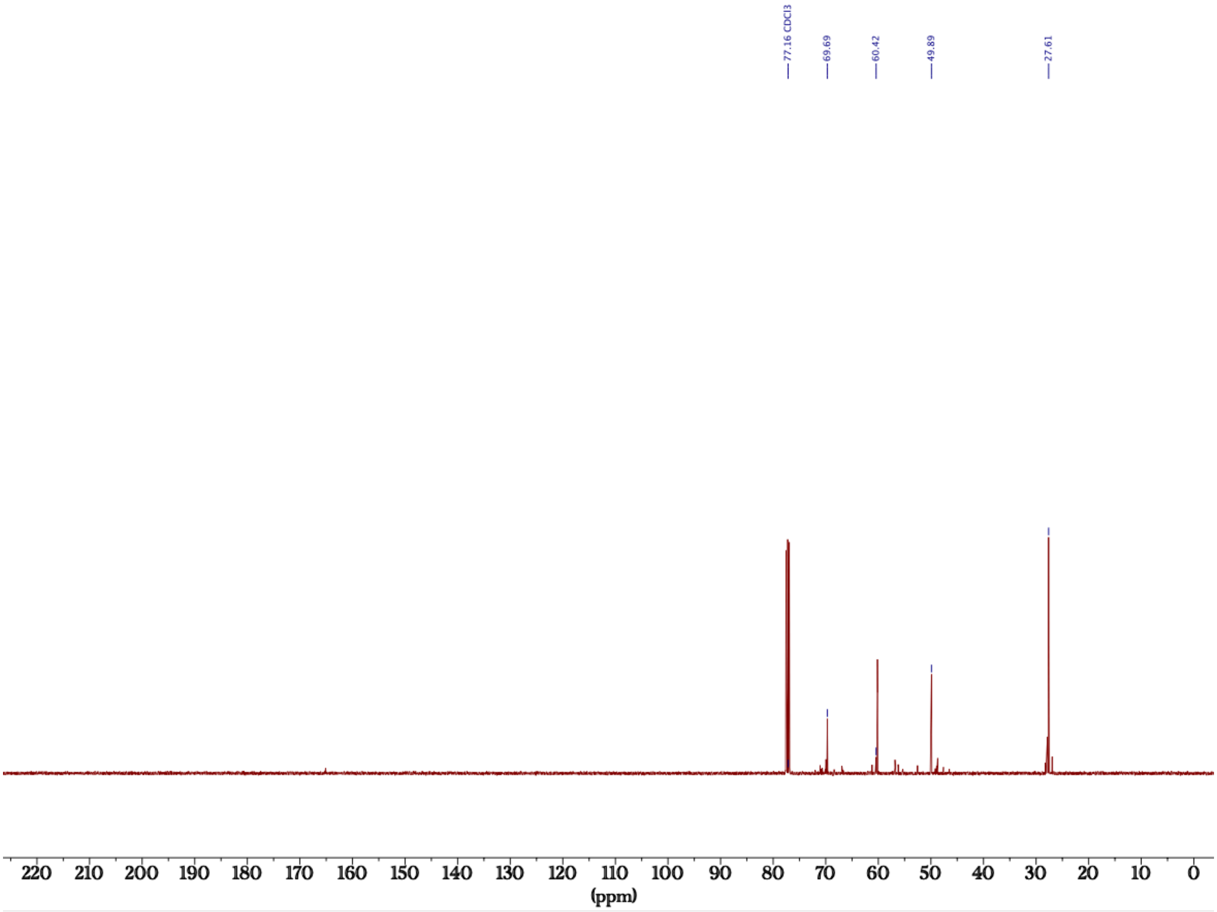

77.16 CDCl3
69.69
60.42
49.89
27.61
(ppm)

Compound 4

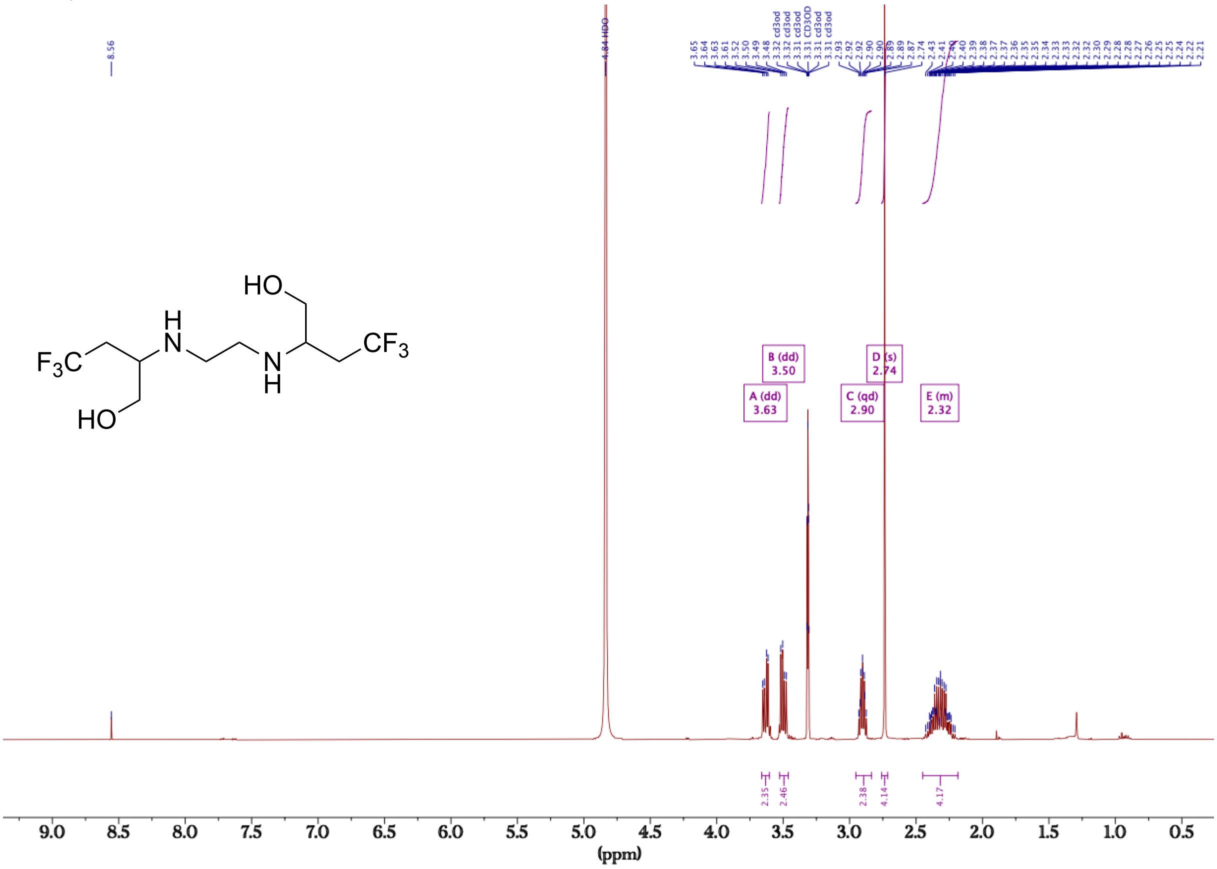

HO
H
N
F3C
N
H
CF3
HO
A (dd)
3.63
B (dd)
3.50
C (qd)
2.90
D (s)
2.74
E (m)
2.32
(ppm)

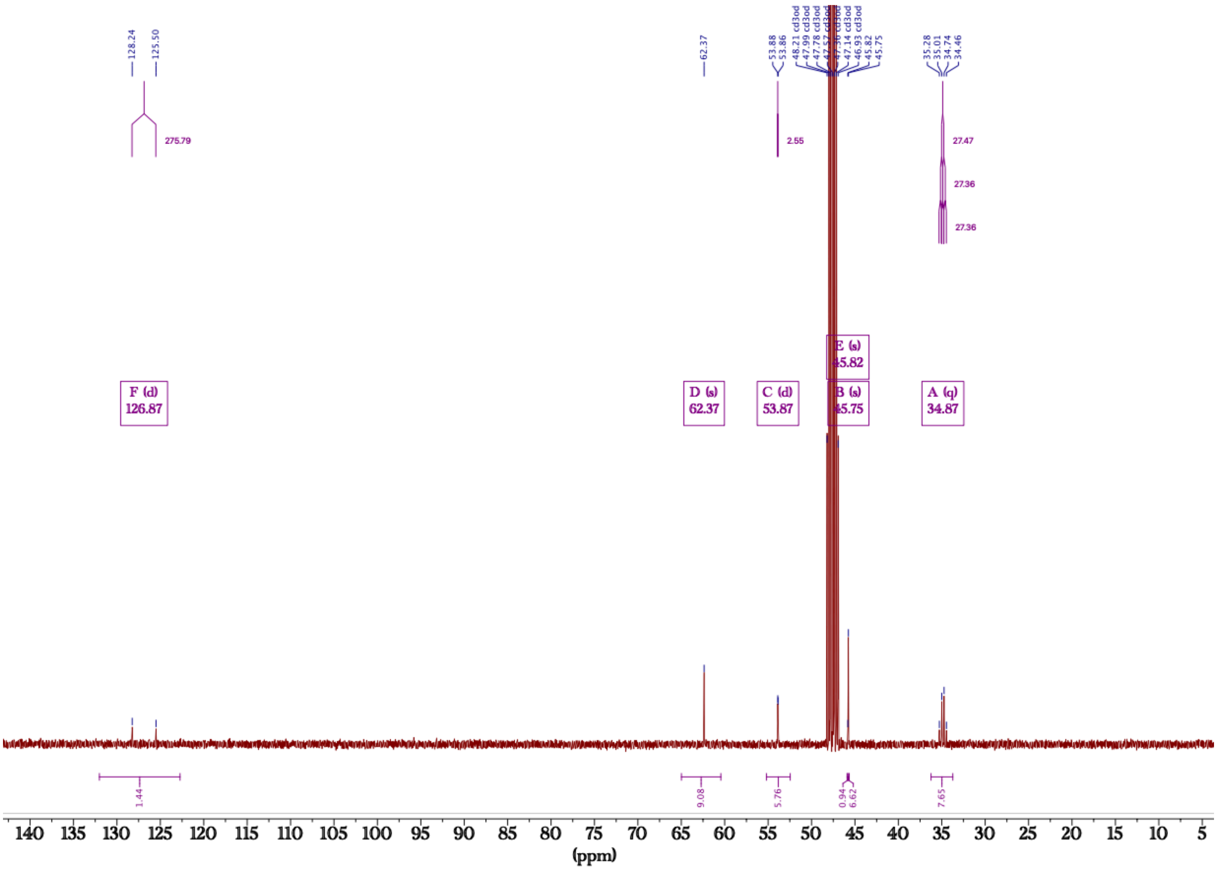

F (d)
126.87
D (s)
62.37
C (d)
53.87
E (s)
45.82
B (s)
45.75
A (q)
34.87
(ppm)

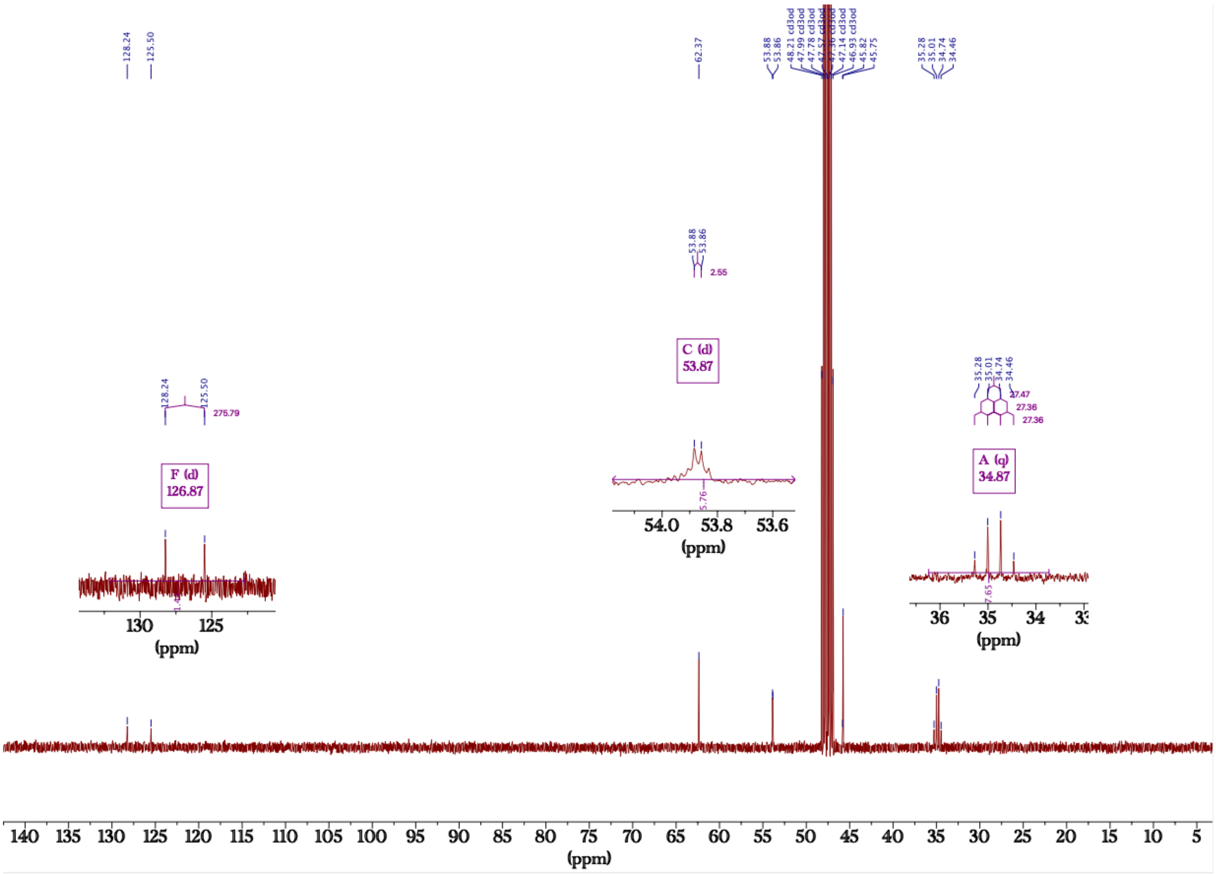

128.24
125.50
62.37
53.88
53.86
48.21 cd3od
47.99 cd3od
47.78 cd3od
47.57 cd3od
47.36 cd3od
47.14 cd3od
46.93 cd3od
45.82
45.75
35.28
35.01
34.74
34.46
2.55
C (d)
53.87
275.79
F (d)
126.87
A (q)
34.87
27.47
27.36
27.36
5.76
7.65
(ppm)

Compound 5

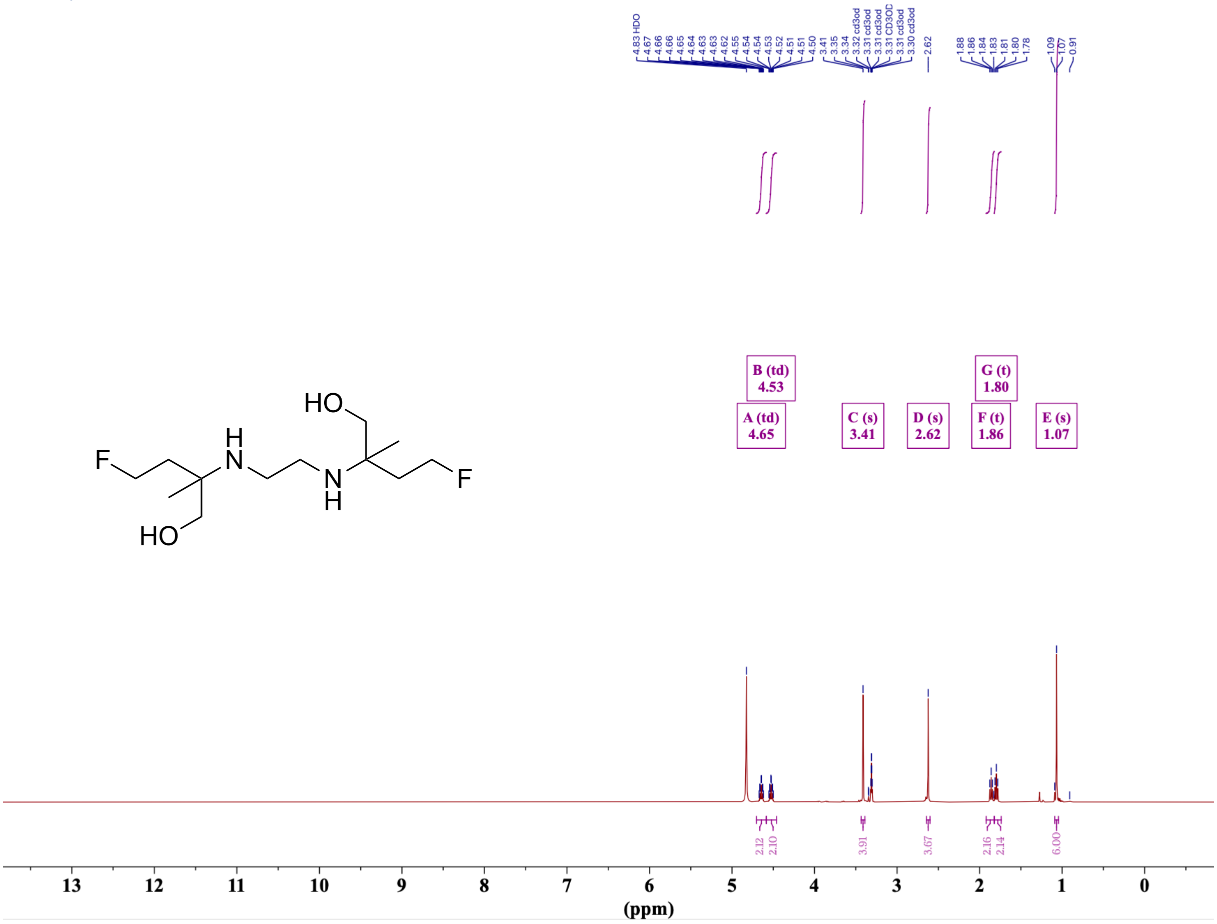

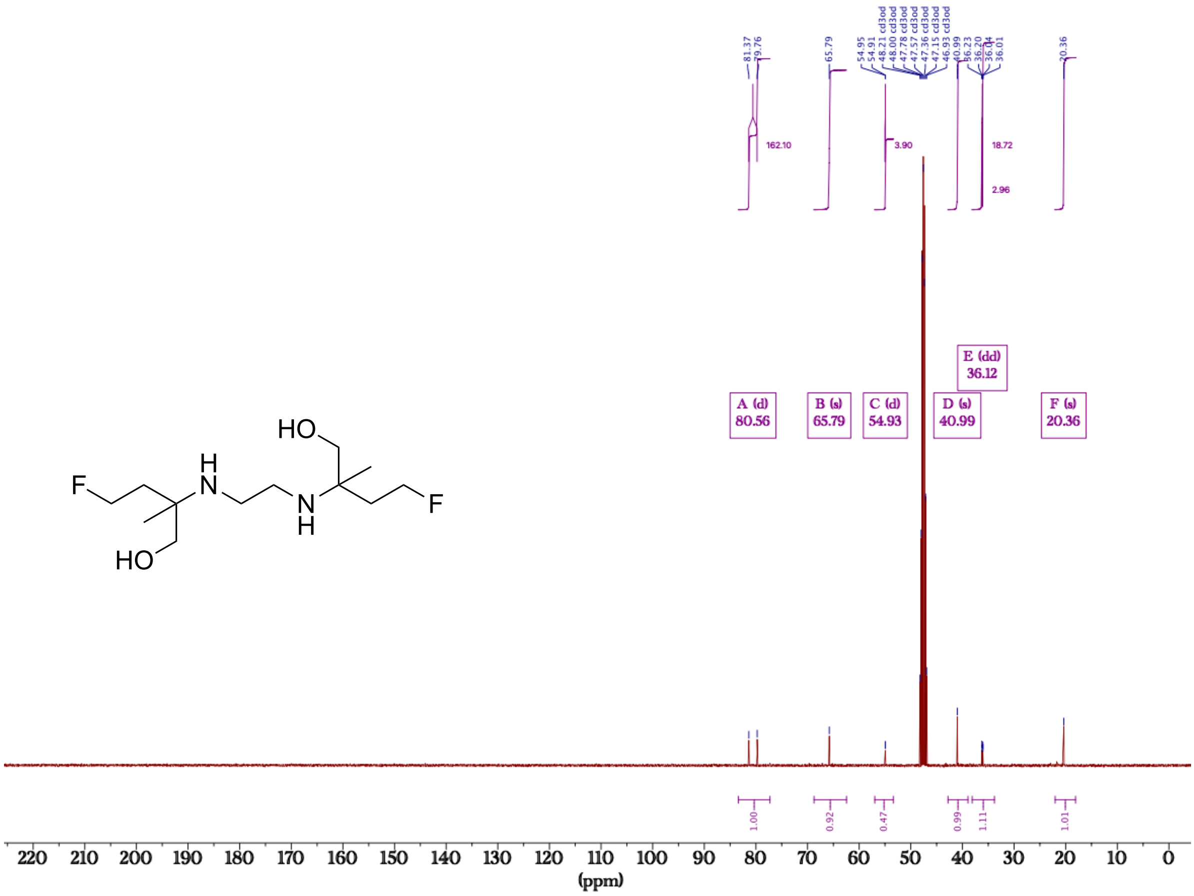

Compound 6

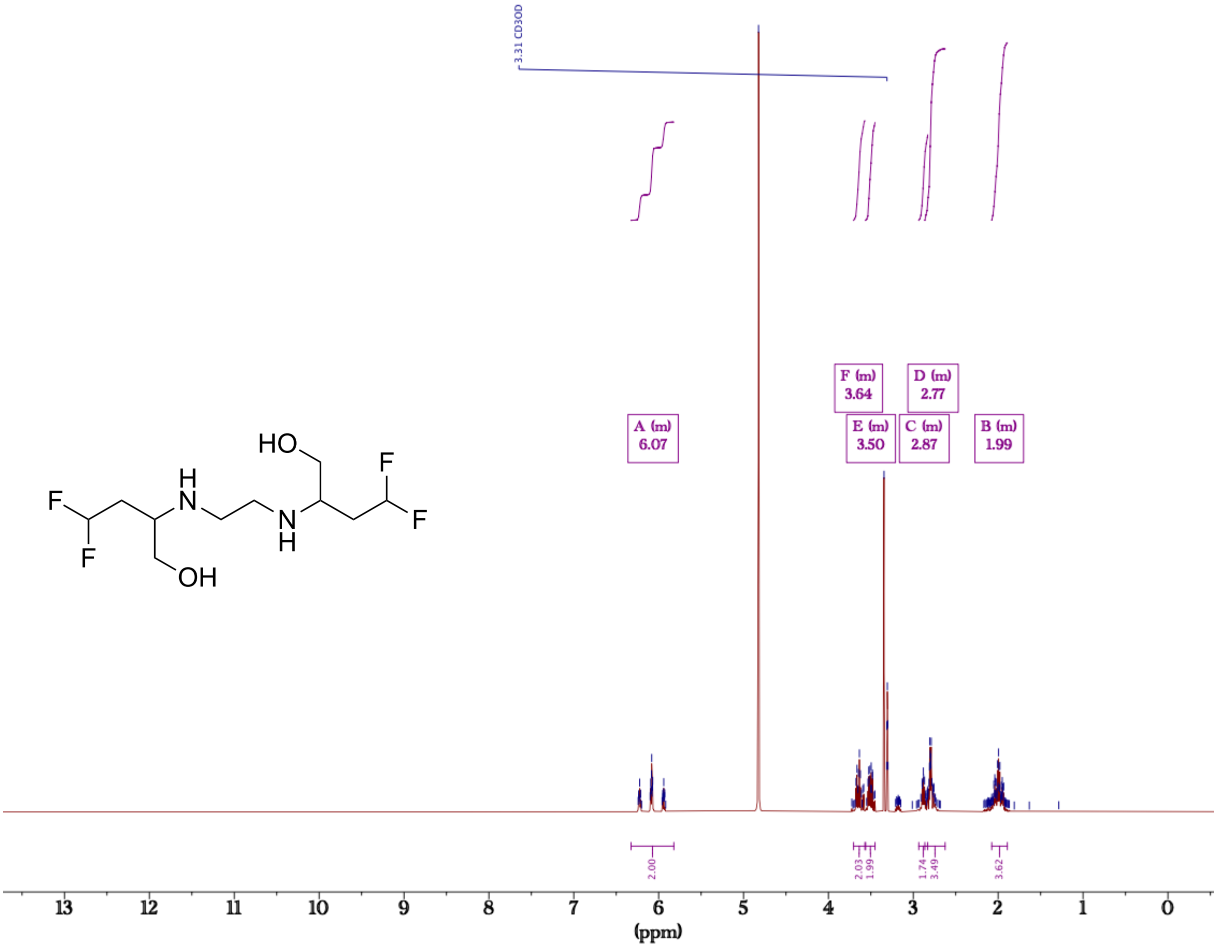
3.31 CD3OD
A (m)
6.07
F (m)
3.64
E (m)
3.50
D (m)
2.77
C (m)
2.87
B (m)
1.99
2.00
2.03
1.99
1.74
3.49
3.62
13
12
11
10
9
8
7
6
5
4
3
2
1
0
(ppm)

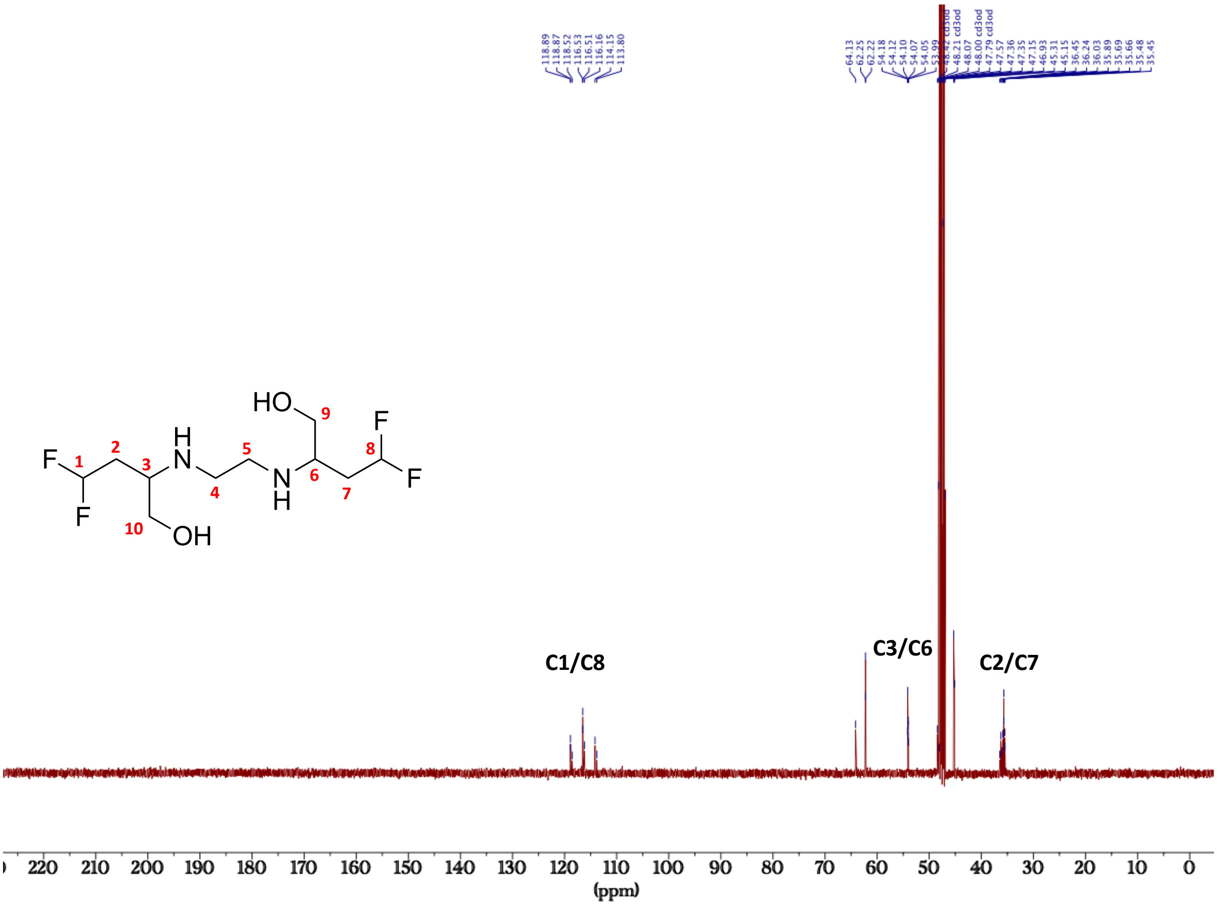

C1/C8
C3/C6
C2/C7
(ppm)

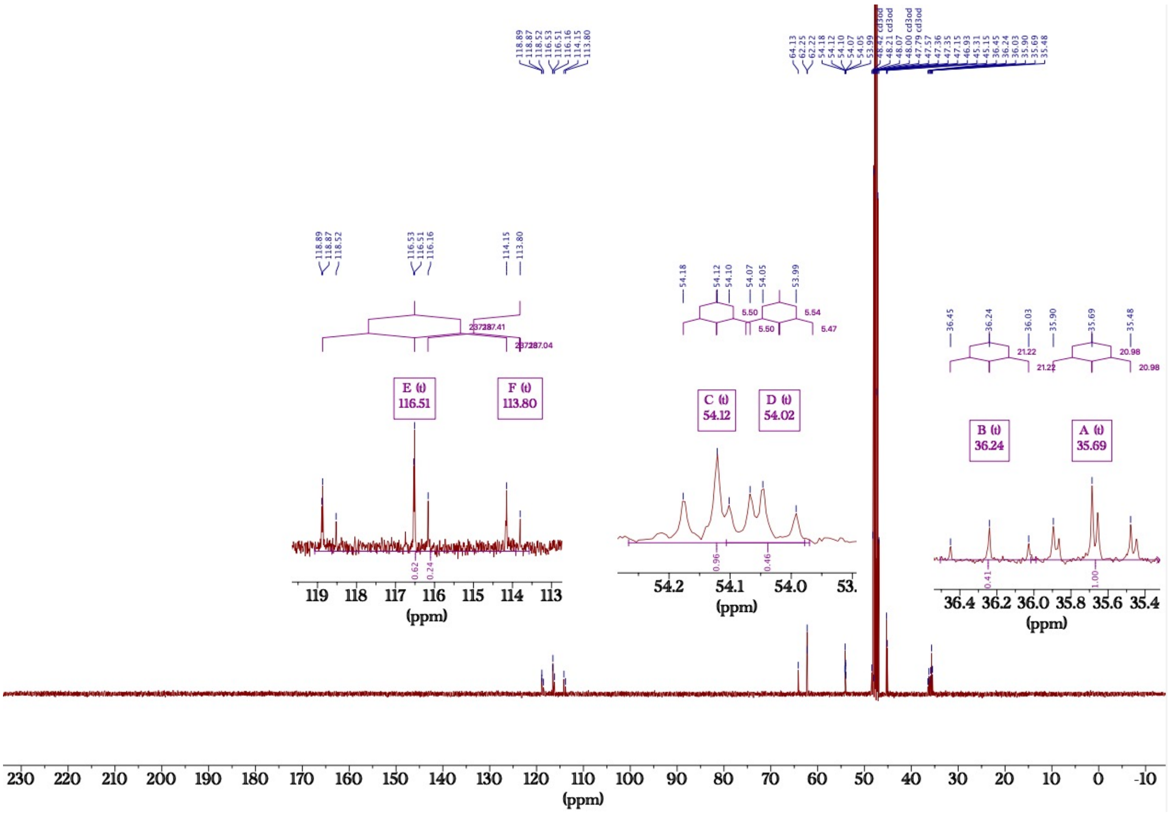

E (t) 116.51
F (t) 113.80
C (t) 54.12
D (t) 54.02
B (t) 36.24
A (t) 35.69
(ppm)

Compound 7

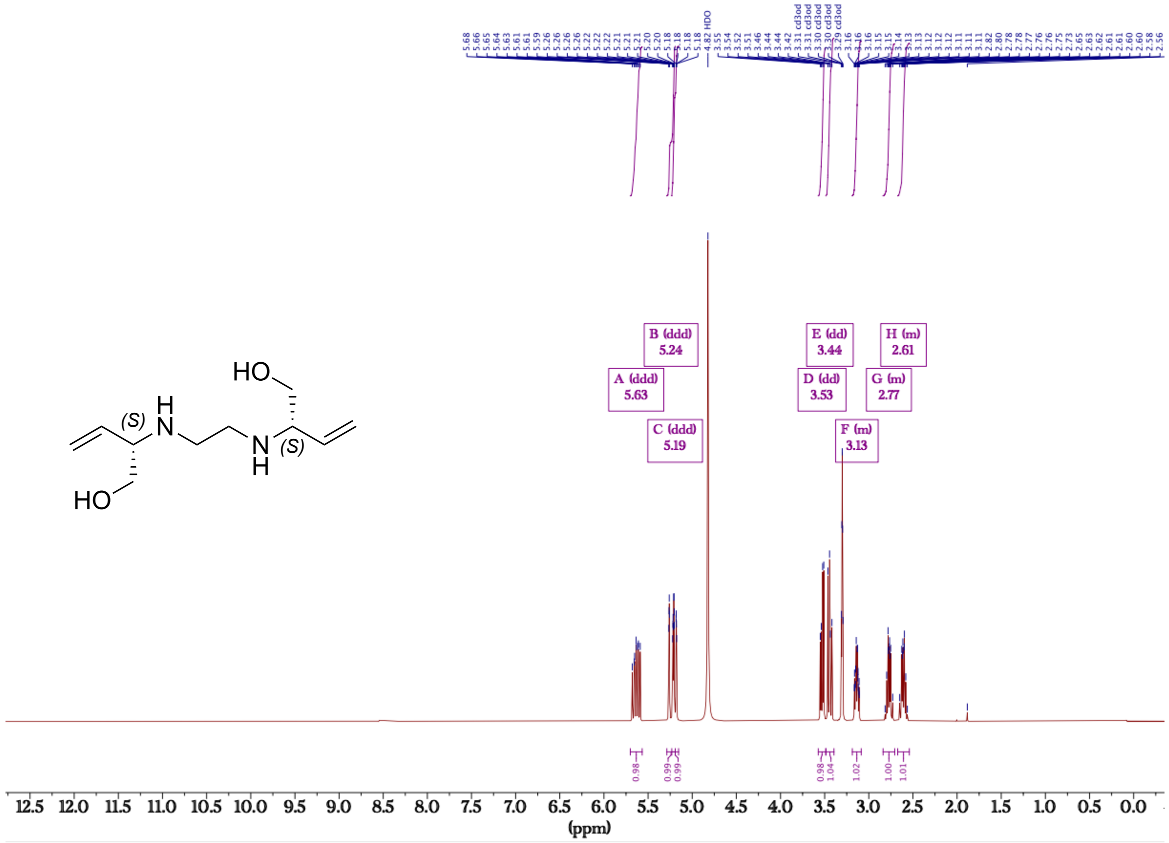

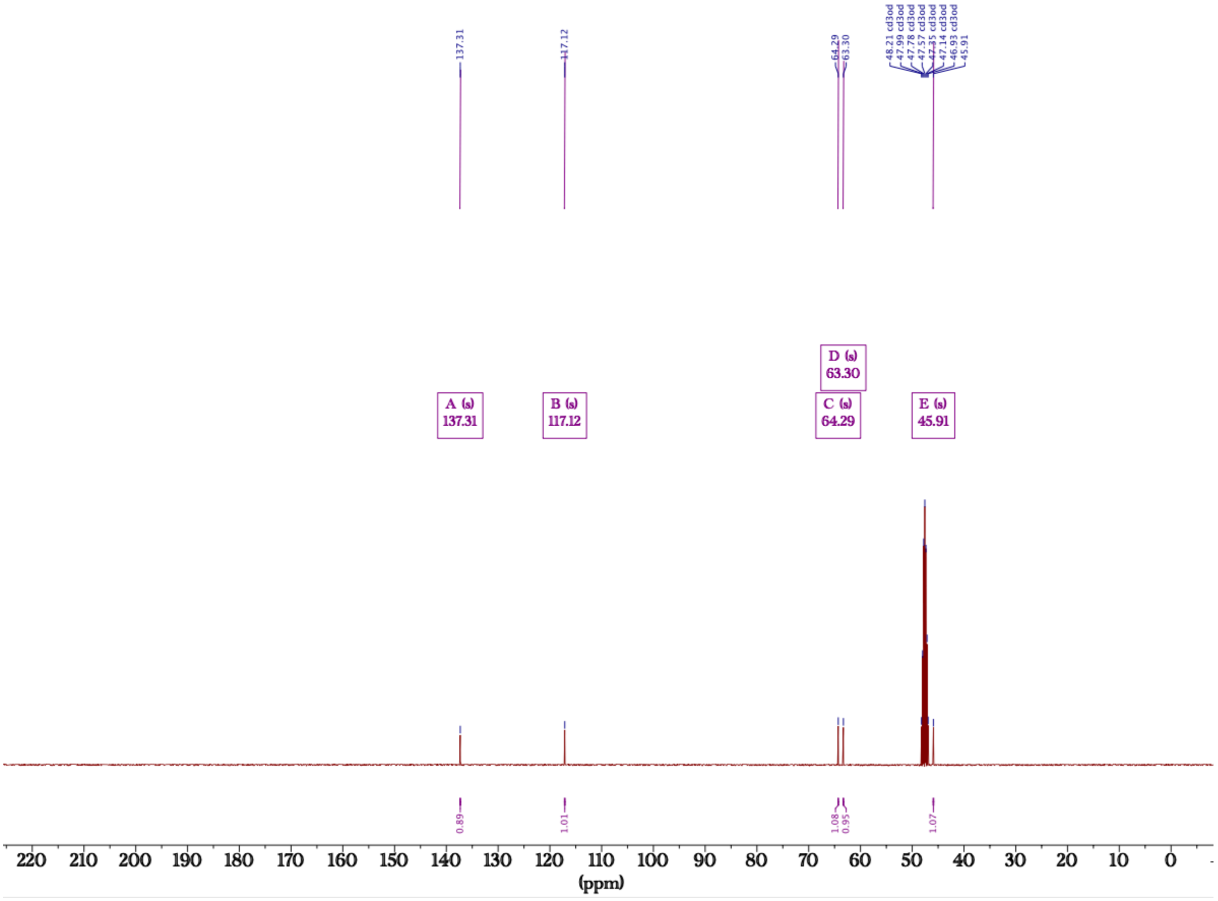

Compound 8

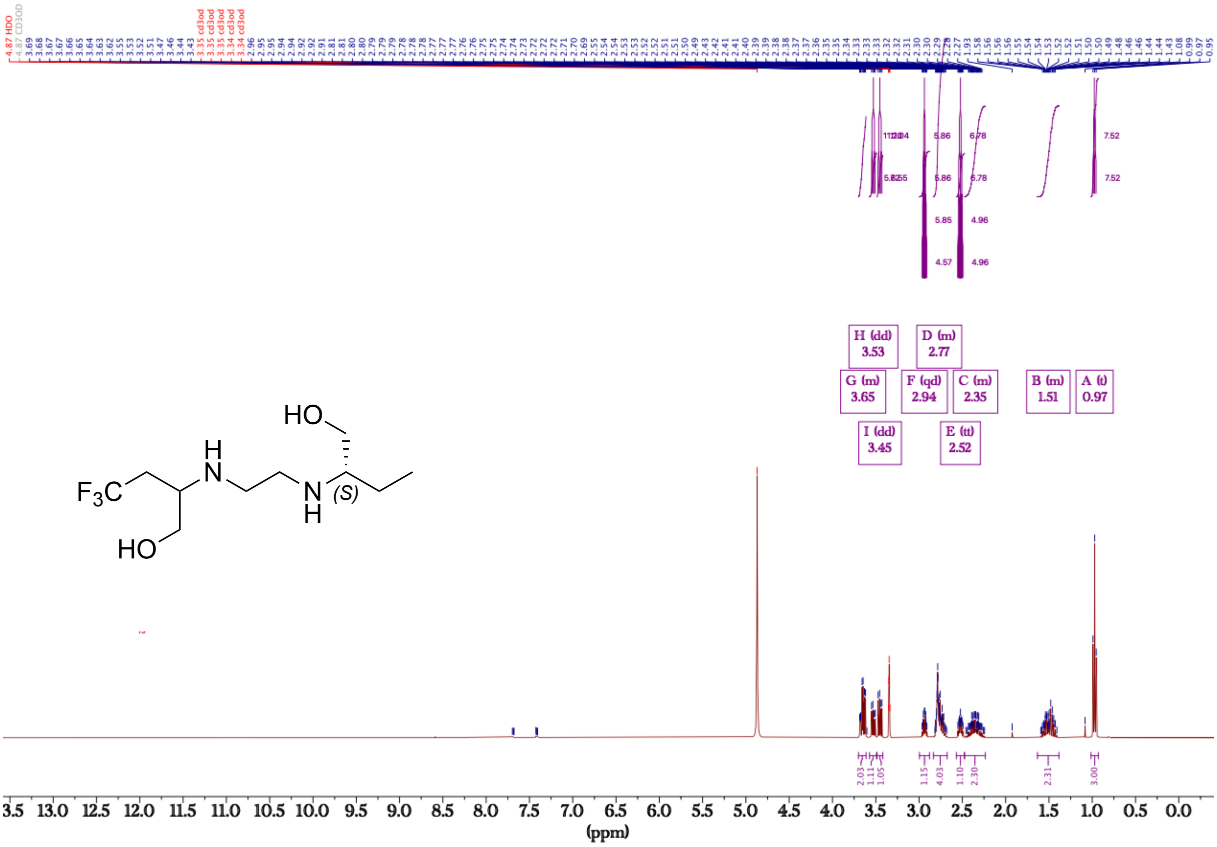

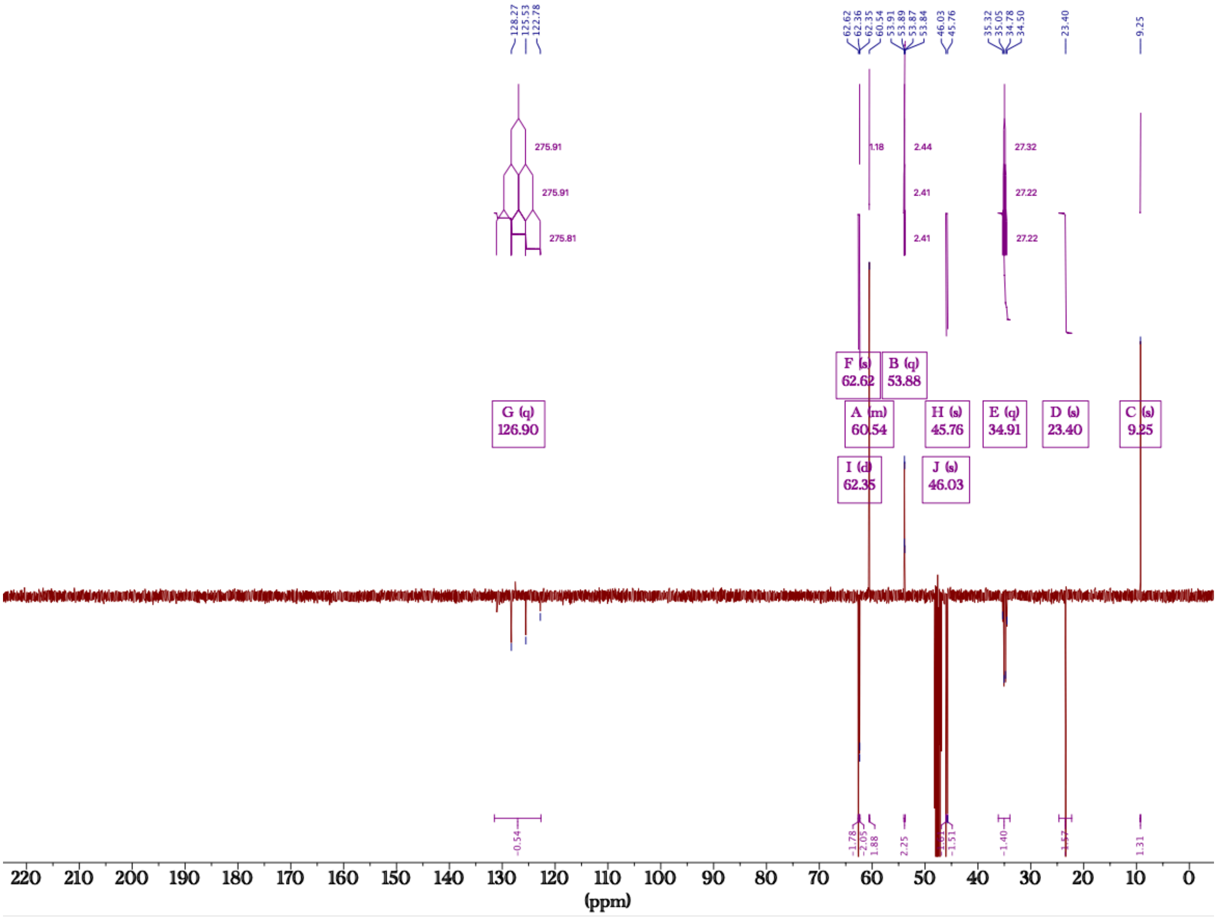

Compound 9

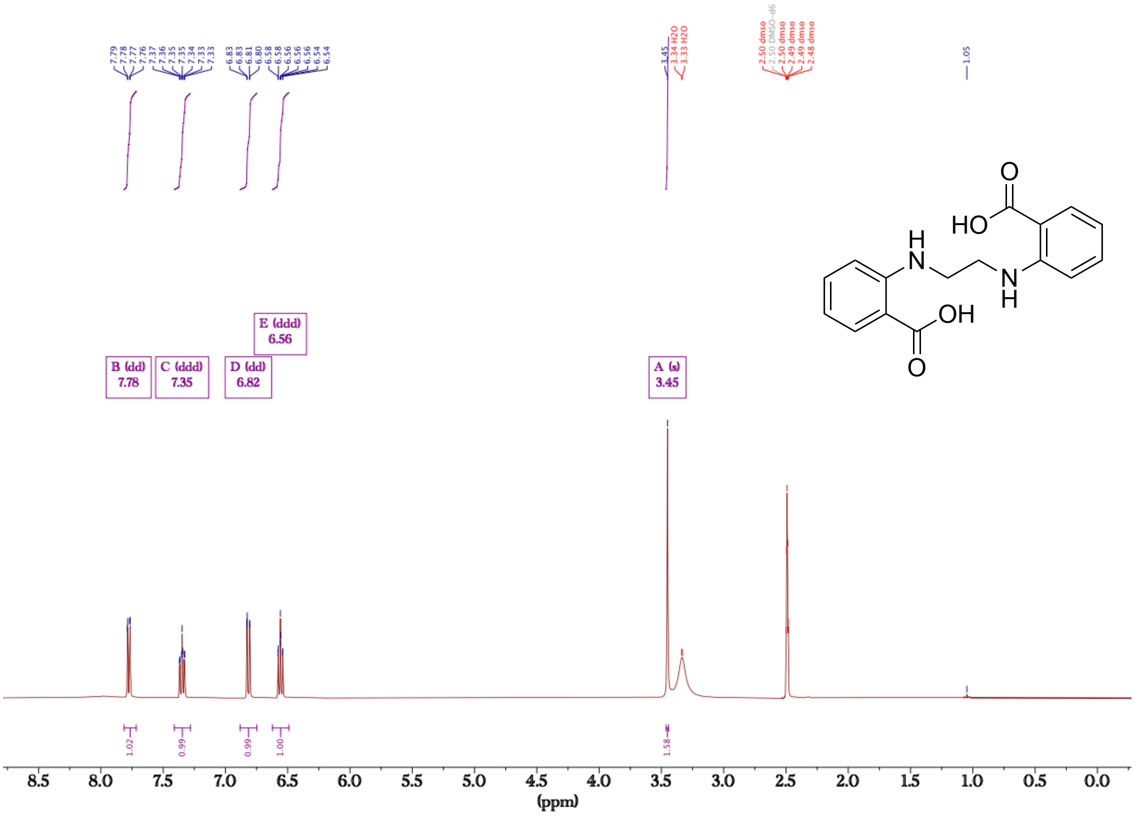

HO
OH
O
O
H
N
N
H
B (dd) 7.78
C (ddd) 7.35
D (dd) 6.82
E (ddd) 6.56
A (s) 3.45
(ppm)

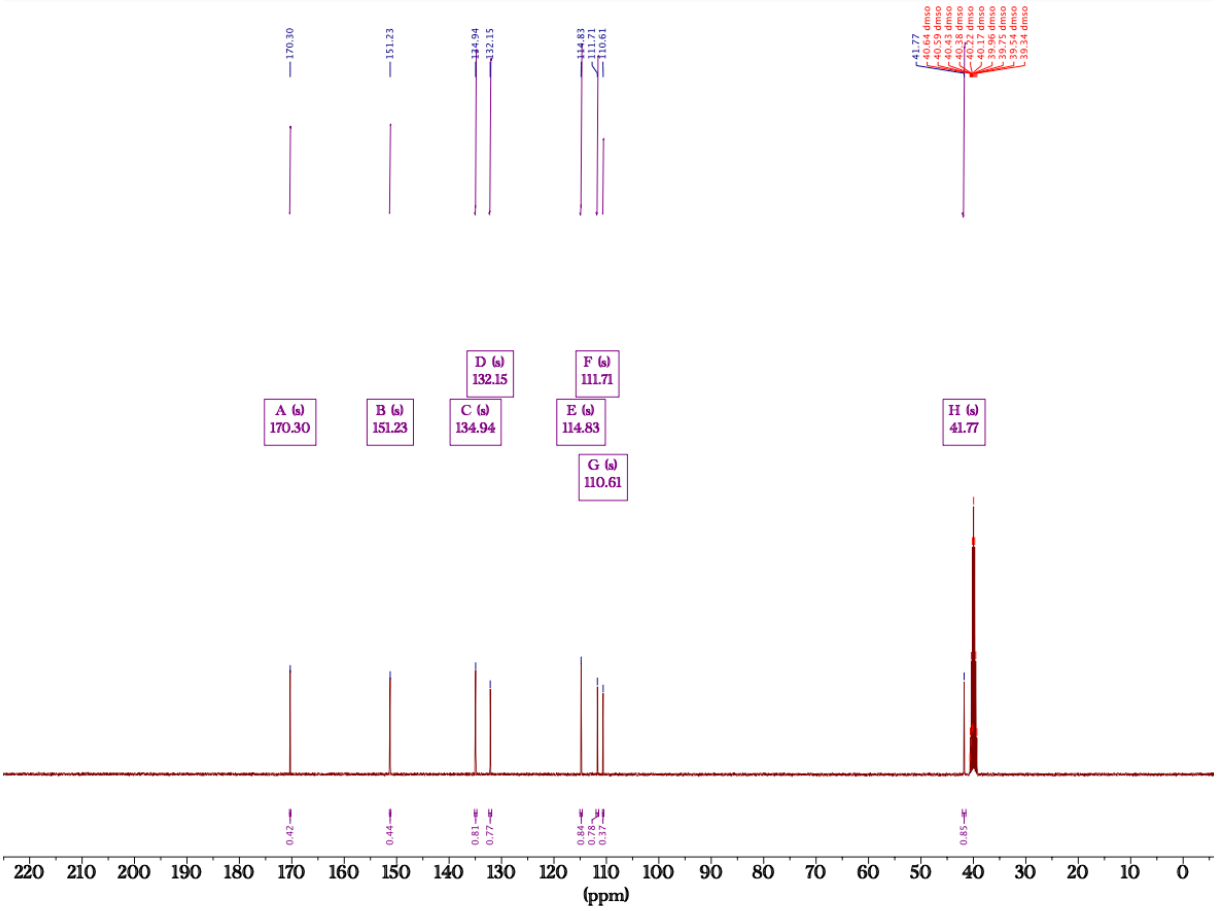

A (s) 170.30
B (s) 151.23
C (s) 134.94
D (s) 132.15
E (s) 114.83
F (s) 111.71
G (s) 110.61
H (s) 41.77
(ppm)

Compound 10

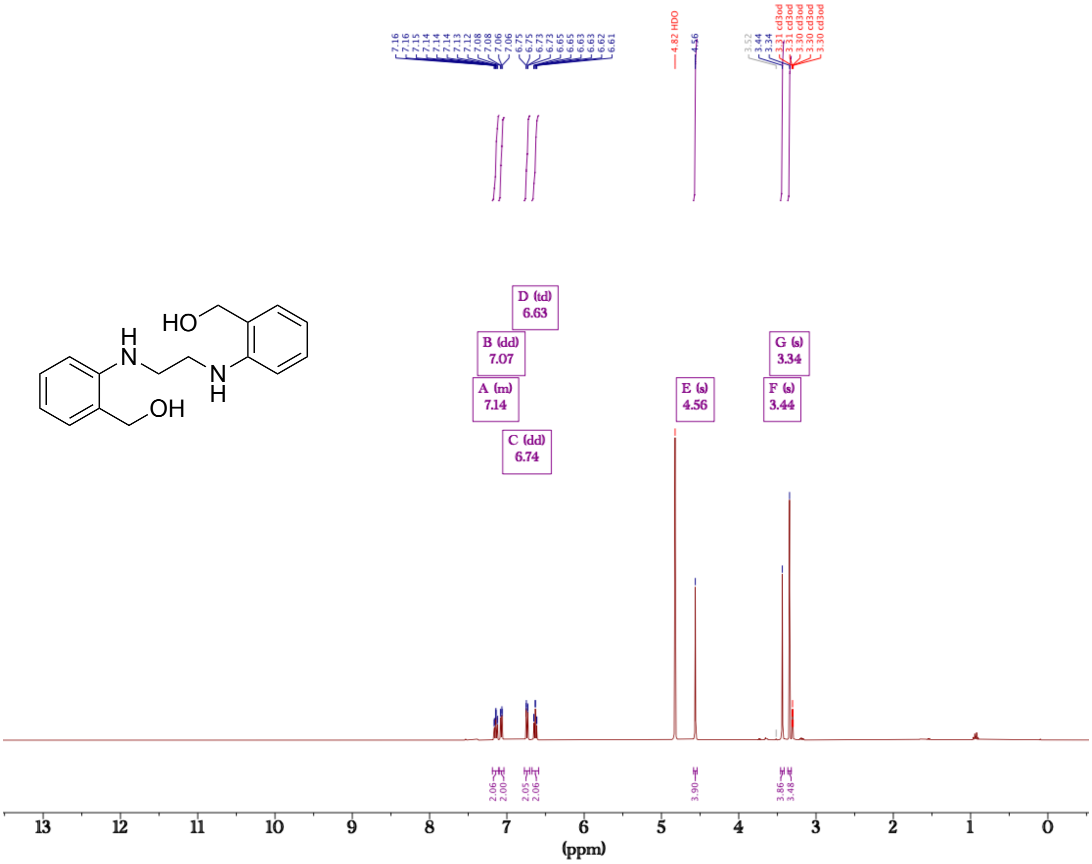

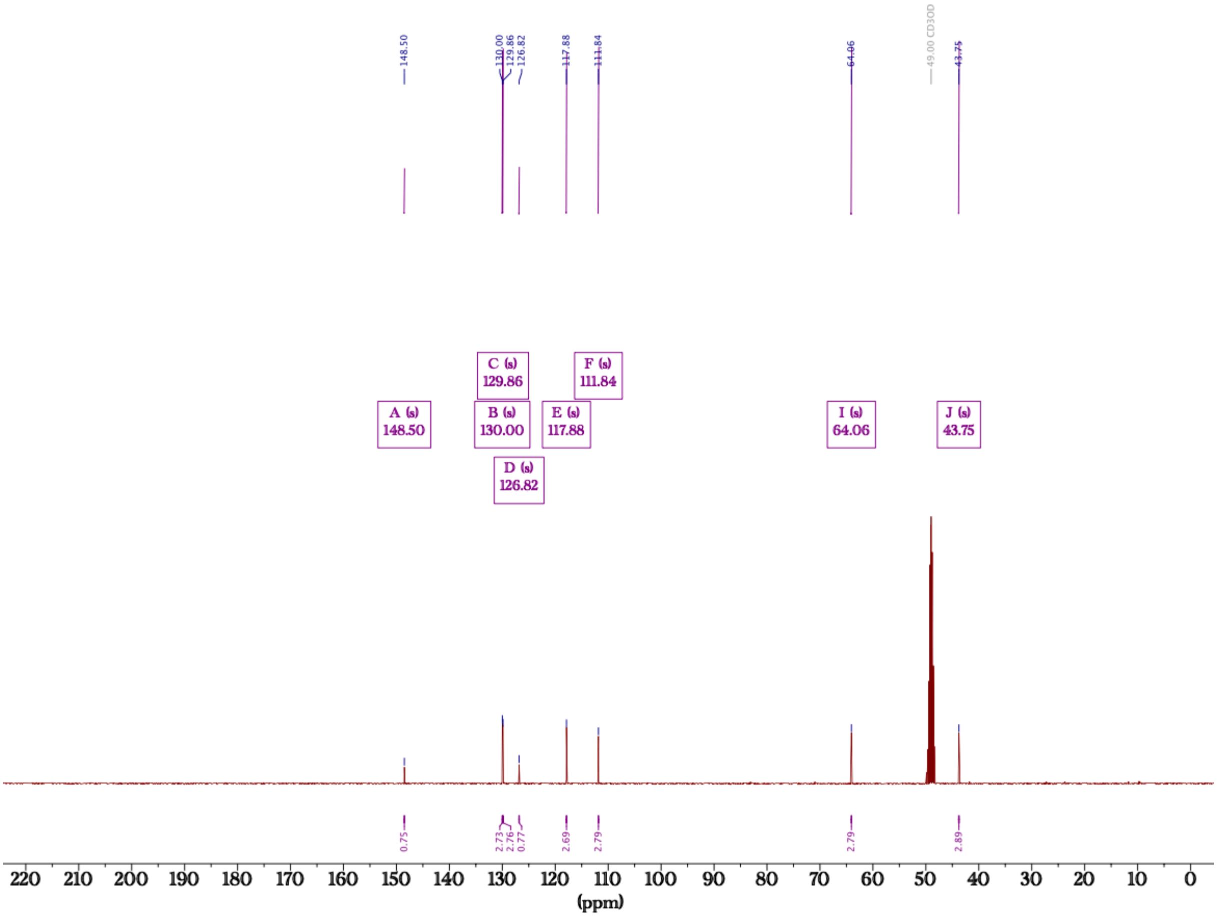

Compound 11

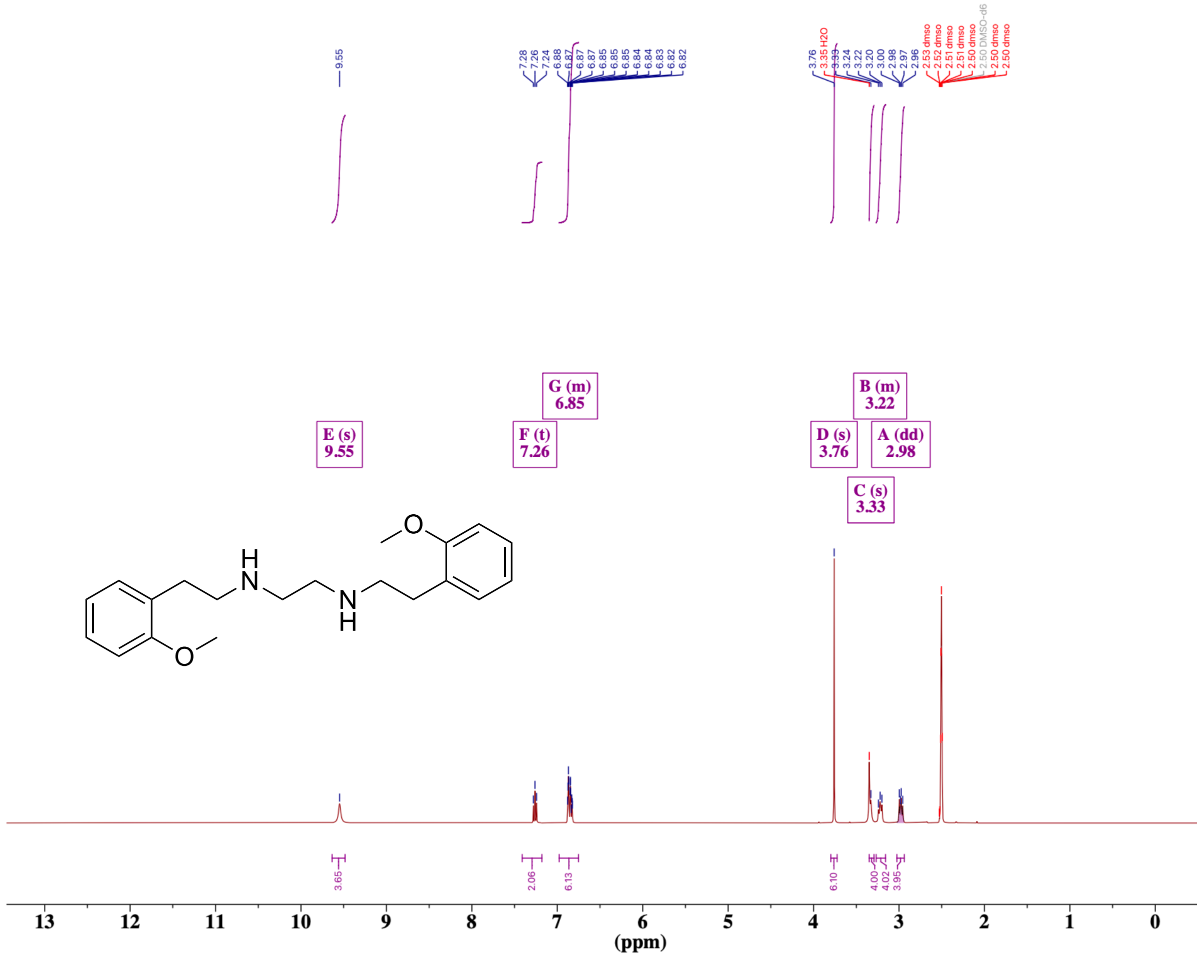

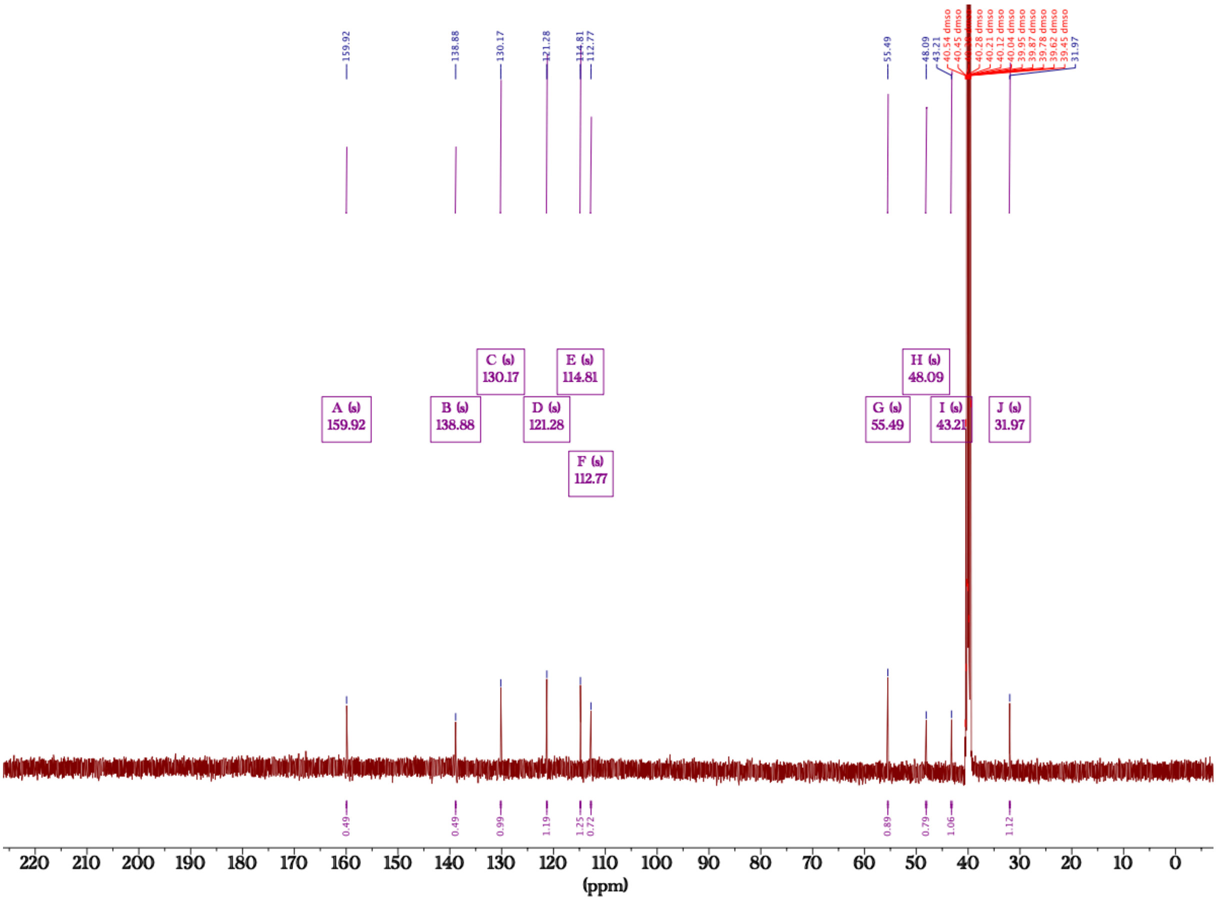
A (s)
159.92
B (s)
138.88
C (s)
130.17
D (s)
121.28
E (s)
114.81
F (s)
112.77
G (s)
55.49
H (s)
48.09
I (s)
43.21
J (s)
31.97
(ppm)

Compound 12

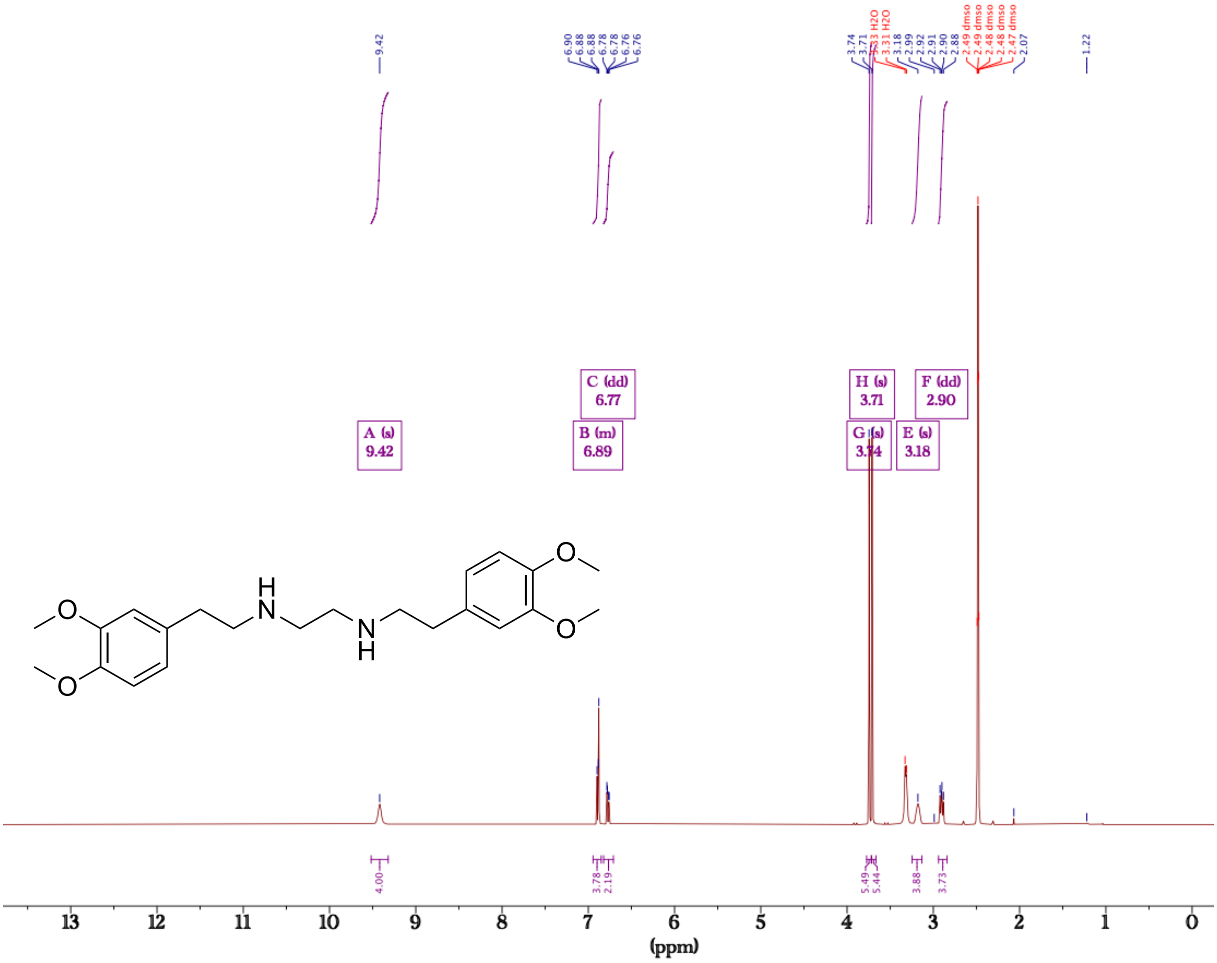

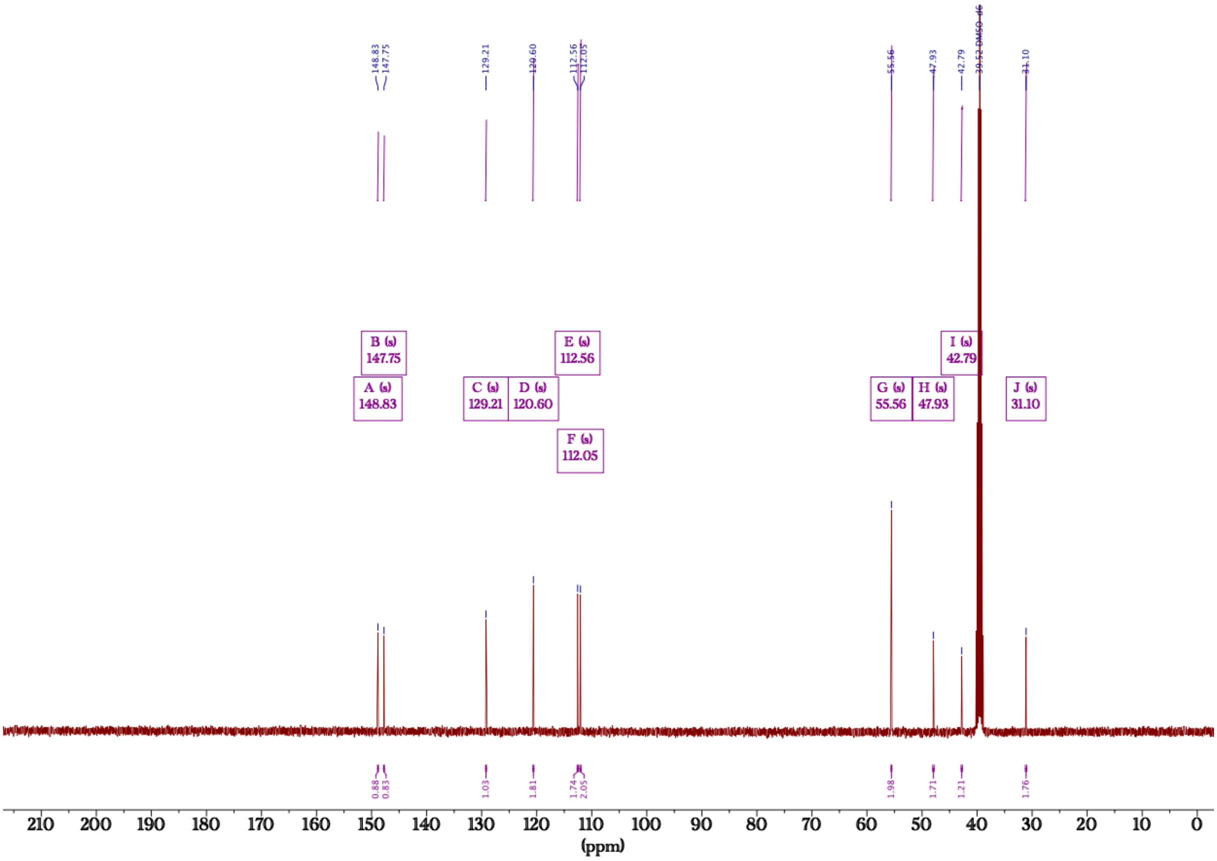

Compound 13

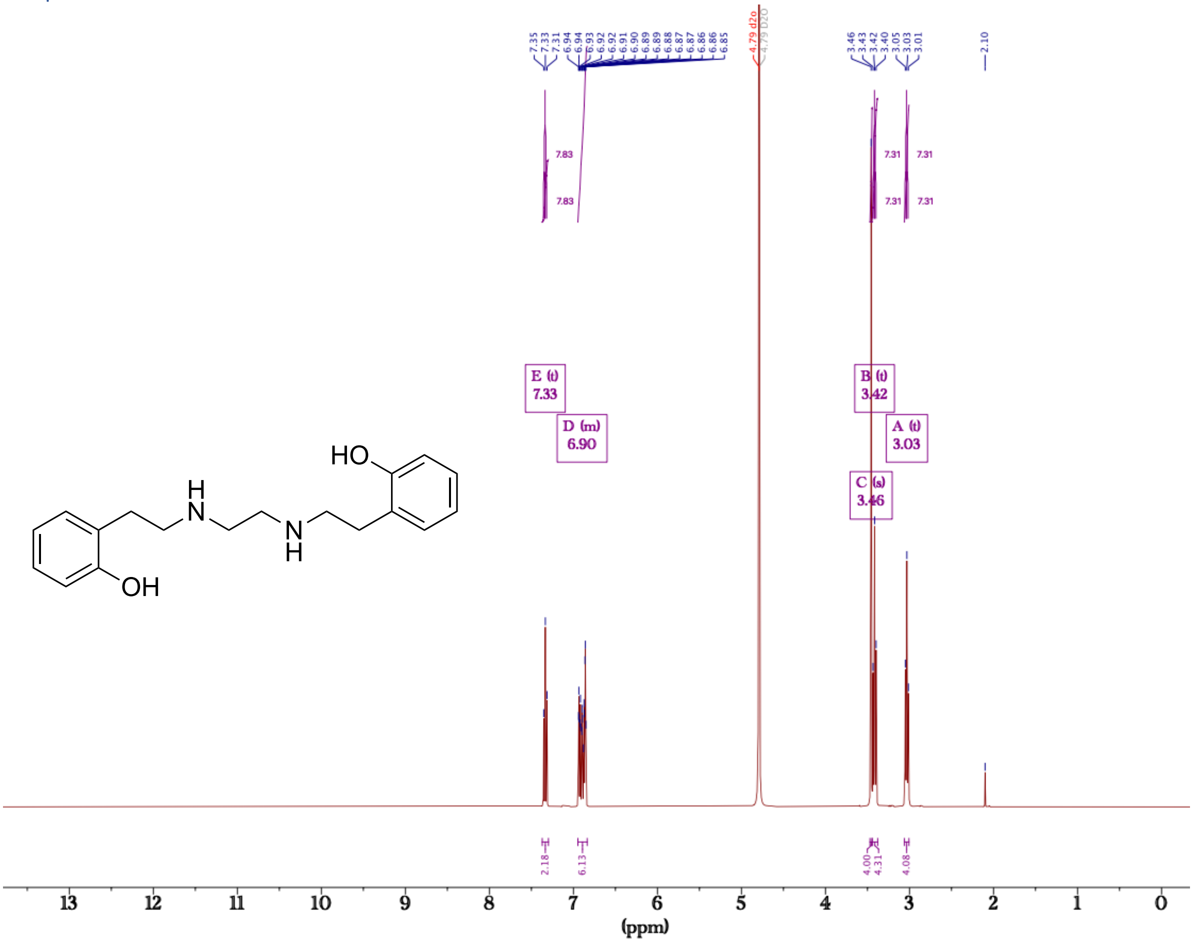

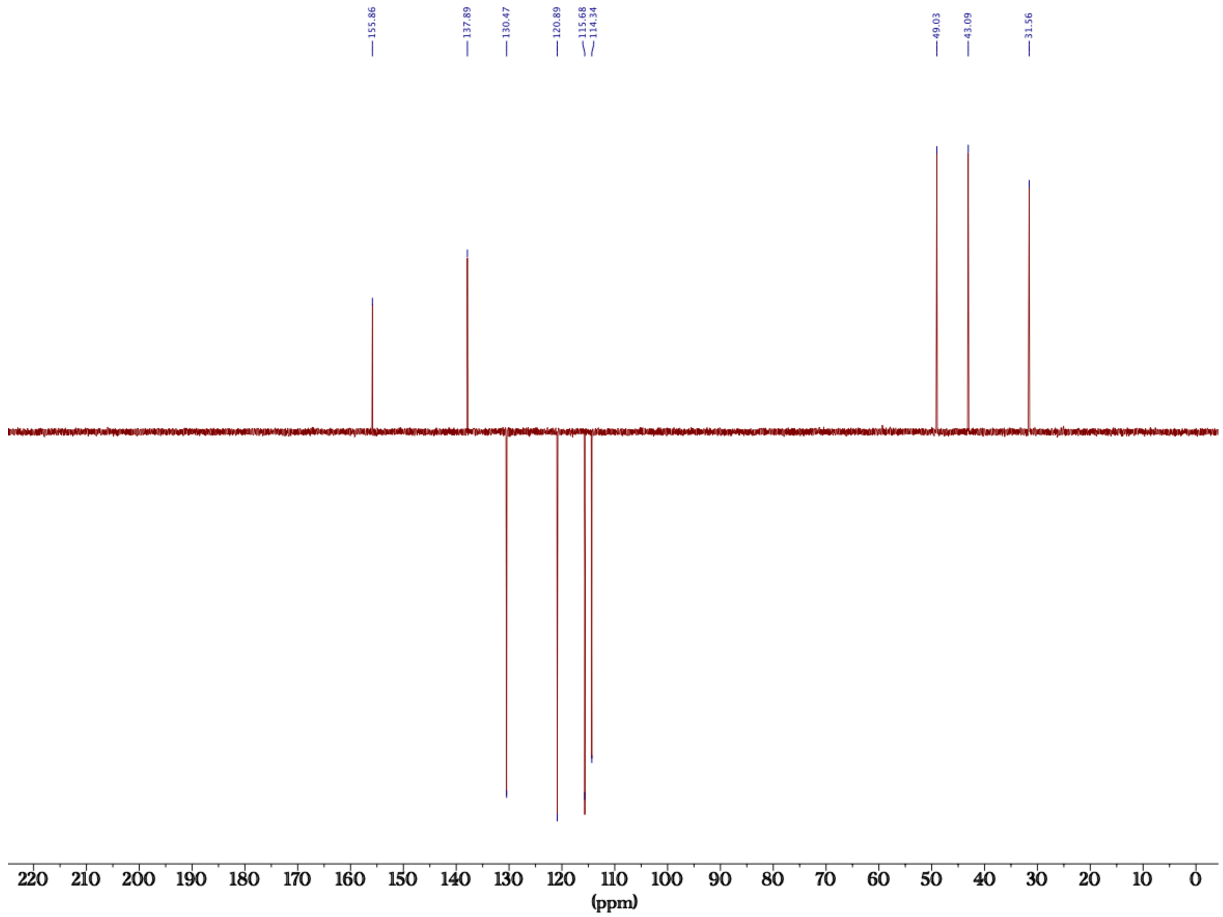
155.86
137.89
130.47
120.89
115.68
114.34
49.03
43.09
31.56
220 210 200 190 180 170 160 150 140 130 120 110 100 90 80 70 60 50 40 30 20 10 0
(ppm)

Compound 14

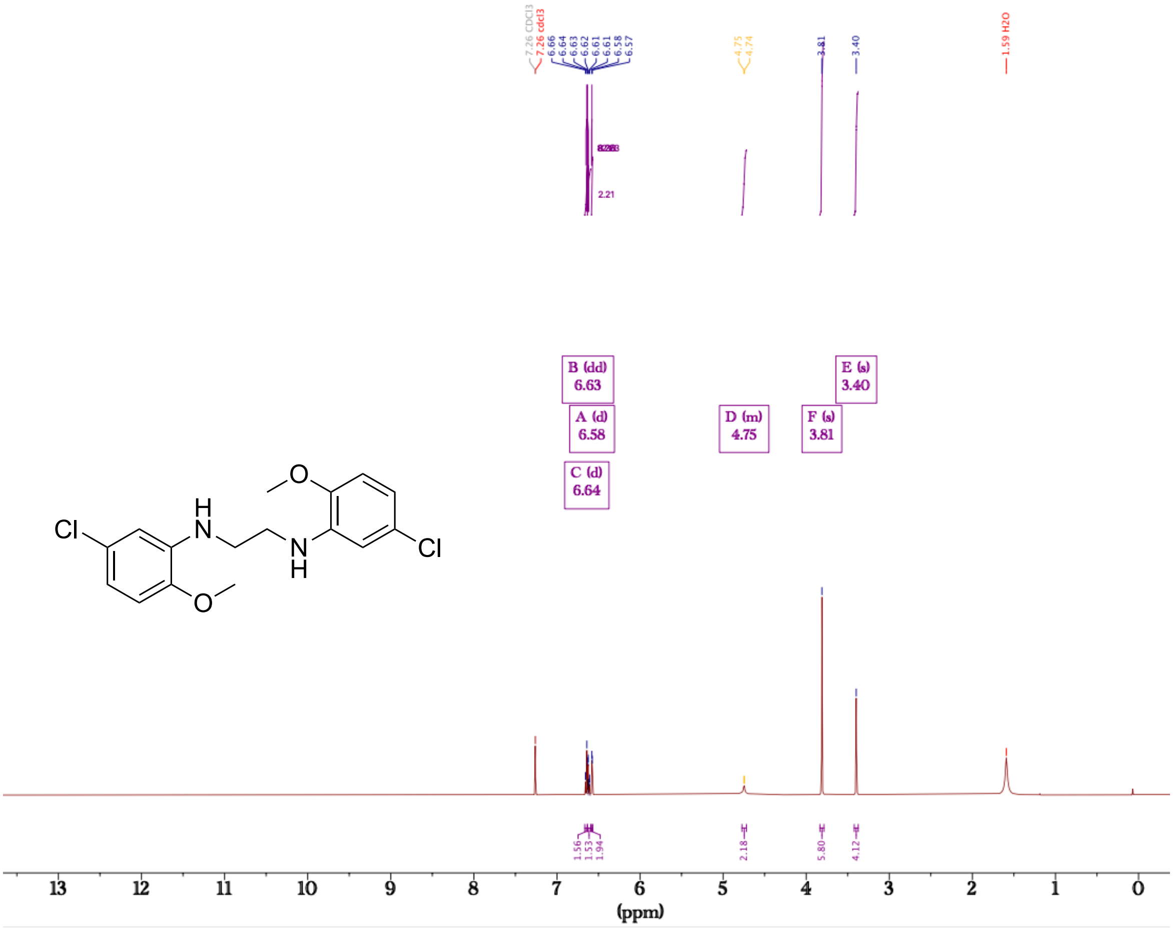

7.26 CDCl3
7.26 cdcl3
6.66
6.64
6.63
6.62
6.61
6.61
6.58
6.57
4.75
4.74
3.81
3.40
1.59 H2O
2.21
B (dd)
6.63
A (d)
6.58
C (d)
6.64
D (m)
4.75
F (s)
3.81
E (s)
3.40
Cl
H
N
N
H
O
O
Cl
1.56
1.53
1.94
2.18
5.80
4.12
13
12
11
10
9
8
7
6
5
4
3
2
1
0
(ppm)

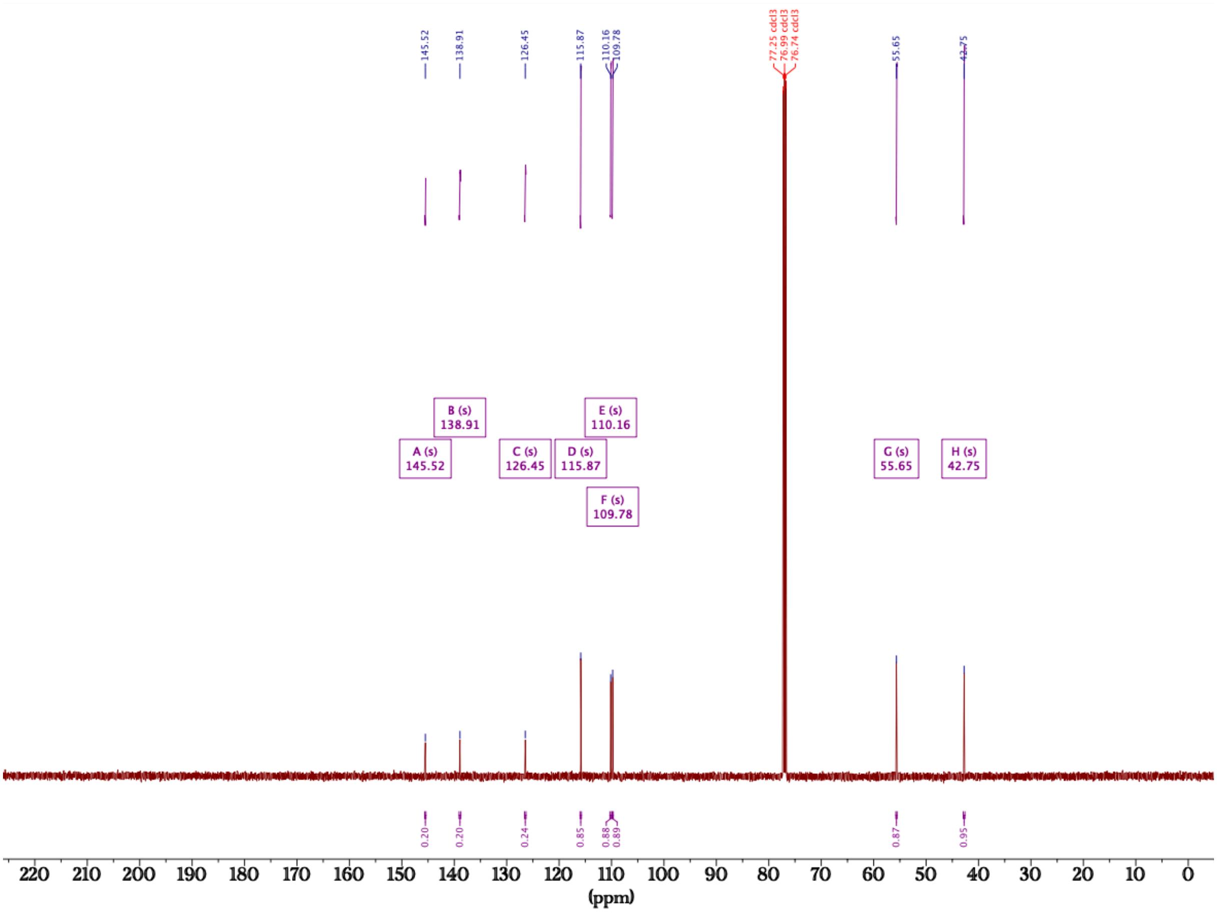

145.52
138.91
126.45
115.87
110.16
109.78
77.25 cdcl3
76.99 cdcl3
76.74 cdcl3
55.65
42.75
B (s) 138.91
E (s) 110.16
A (s) 145.52
C (s) 126.45
D (s) 115.87
G (s) 55.65
H (s) 42.75
F (s) 109.78
0.20
0.20
0.24
0.85
0.88
0.89
0.87
0.95
220 210 200 190 180 170 160 150 140 130 120 110 100 90 80 70 60 50 40 30 20 10 0
(ppm)

Compound 15

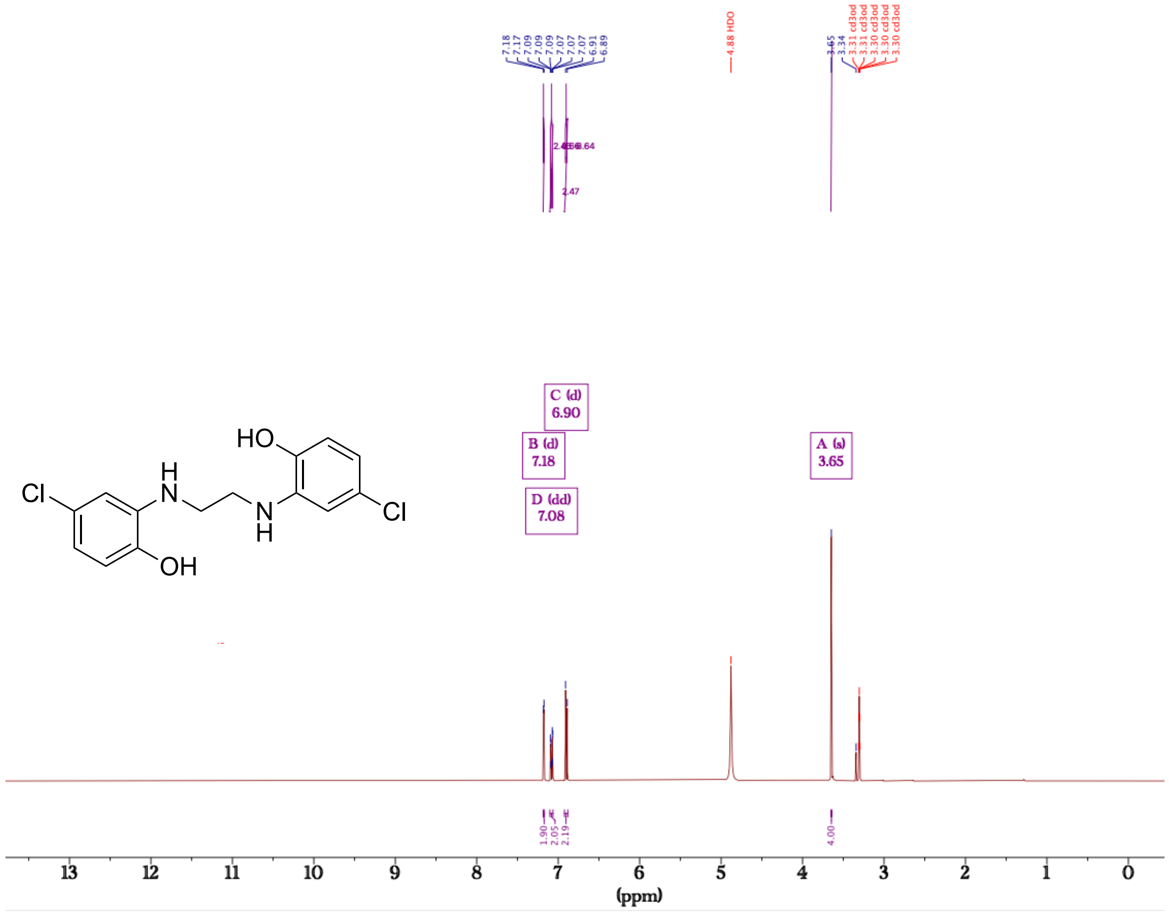

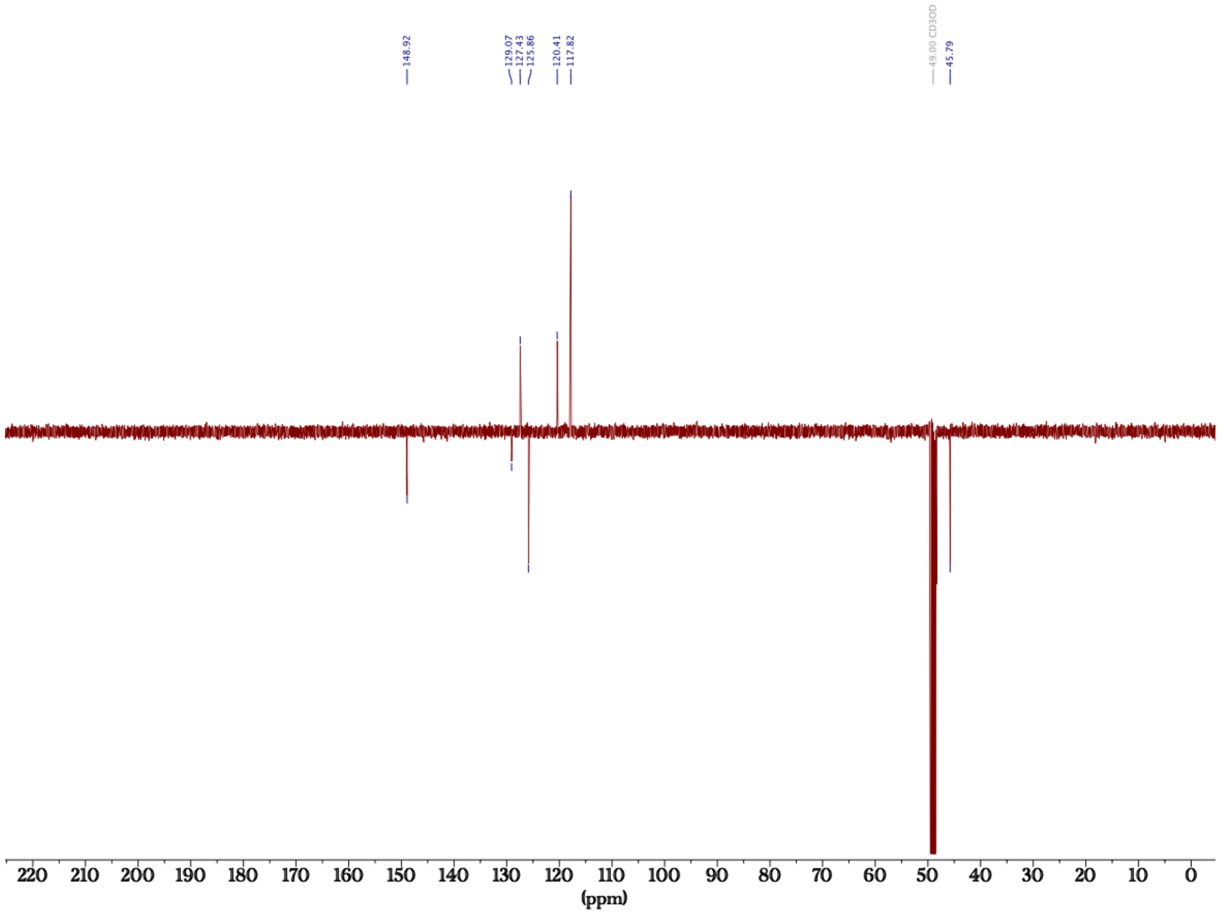
148.92
129.07
127.43
125.86
120.41
117.82
49.00 CD3OD
45.79
220 210 200 190 180 170 160 150 140 130 120 110 100 90 80 70 60 50 40 30 20 10 0
(ppm)

Compound 16

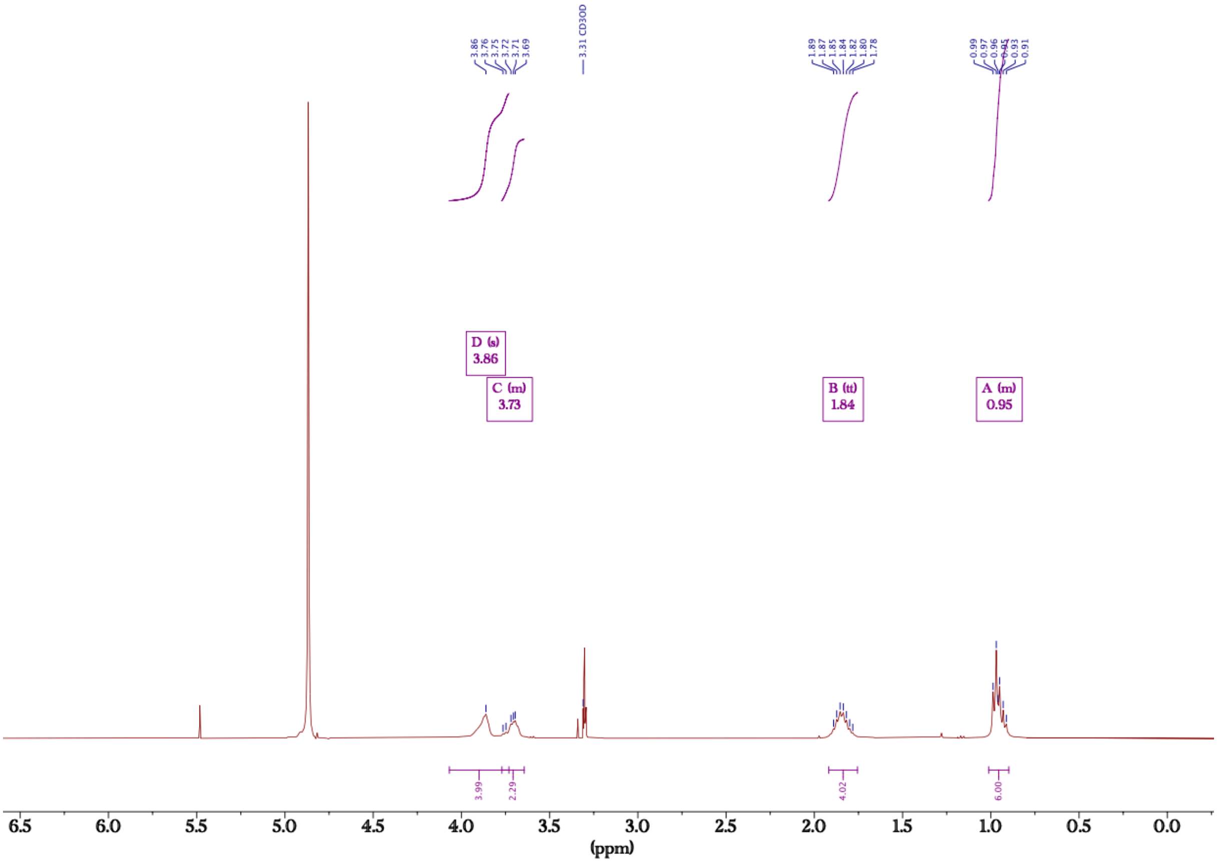
3.86
3.76
3.75
3.72
3.71
3.69
3.31 CD3OD
1.89
1.87
1.85
1.84
1.82
1.80
1.78
0.99
0.97
0.96
0.93
0.91
D (s)
3.86
C (m)
3.73
B (tt)
1.84
A (m)
0.95
3.99
2.29
4.02
6.00
6.5 6.0 5.5 5.0 4.5 4.0 3.5 3.0 2.5 2.0 1.5 1.0 0.5 0.0
(ppm)

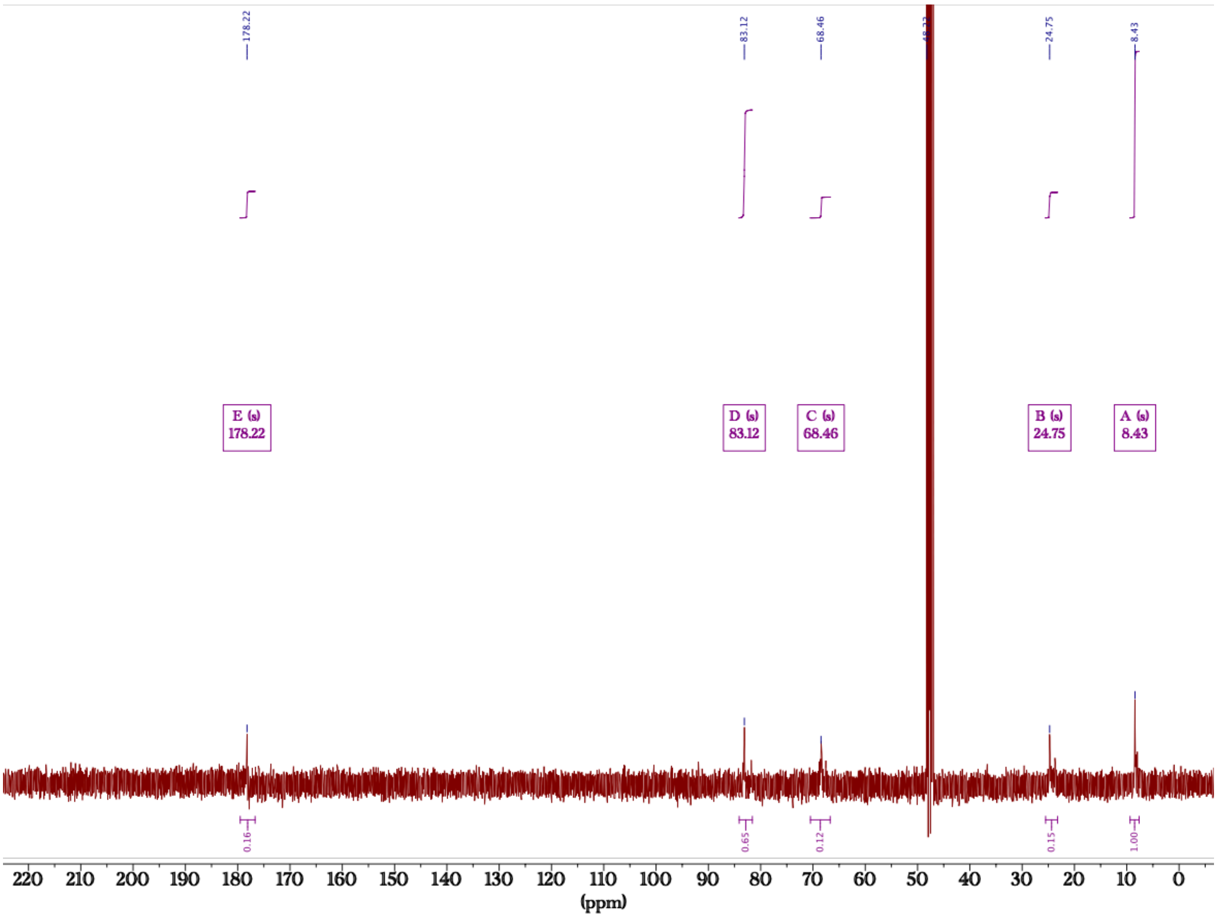
178.22
83.12
68.46
24.75
8.43
E (s)
178.22
D (s)
83.12
C (s)
68.46
B (s)
24.75
A (s)
8.43
0.16
0.65
0.12
0.15
1.00
220 210 200 190 180 170 160 150 140 130 120 110 100 90 80 70 60 50 40 30 20 10 0
(ppm)

Compound 17

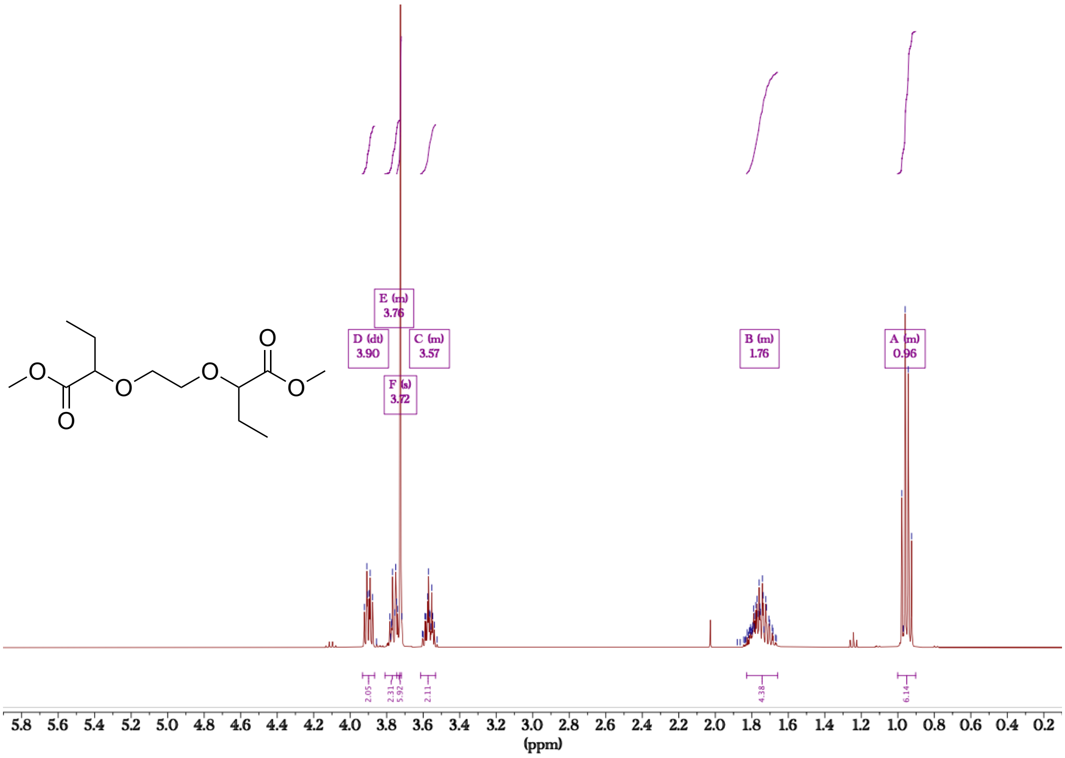

E (m) 3.76
D (dt) 3.90
C (m) 3.57
F (s) 3.72
B (m) 1.76
A (m) 0.96
2.05
2.31
5.92
2.11
4.38
6.14
5.8 5.6 5.4 5.2 5.0 4.8 4.6 4.4 4.2 4.0 3.8 3.6 3.4 3.2 3.0 2.8 2.6 2.4 2.2 2.0 1.8 1.6 1.4 1.2 1.0 0.8 0.6 0.4 0.2
(ppm)

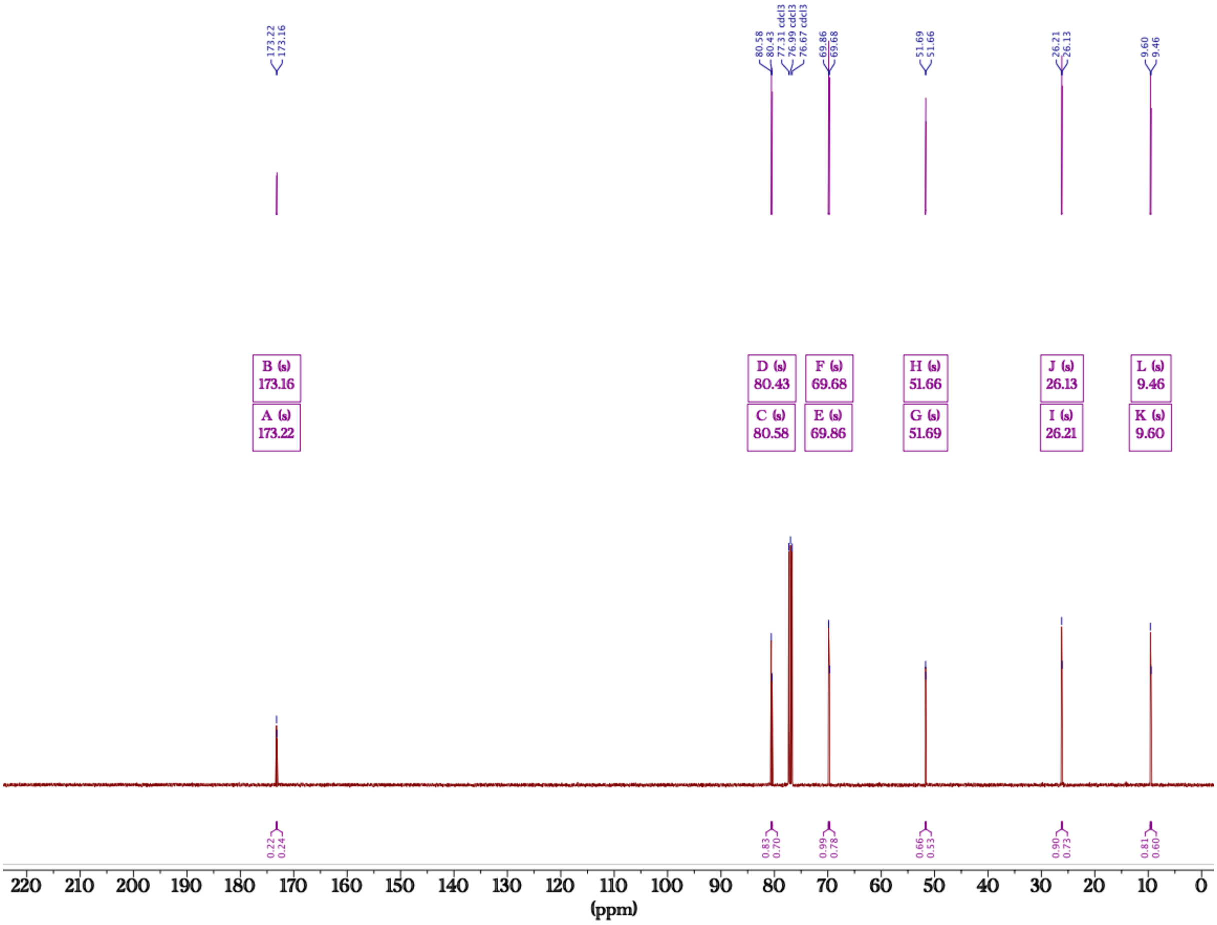

173.22
173.16
80.58
80.43
77.31 cdcl3
76.99 cdcl3
76.67 cdcl3
69.86
69.68
51.69
51.66
26.21
26.13
9.60
9.46
B (s) 173.16
A (s) 173.22
D (s) 80.43
C (s) 80.58
F (s) 69.68
E (s) 69.86
H (s) 51.66
G (s) 51.69
J (s) 26.13
I (s) 26.21
L (s) 9.46
K (s) 9.60
0.22
0.24
0.83
0.70
0.99
0.78
0.66
0.53
0.90
0.73
0.81
0.60
220 210 200 190 180 170 160 150 140 130 120 110 100 90 80 70 60 50 40 30 20 10 0
(ppm)

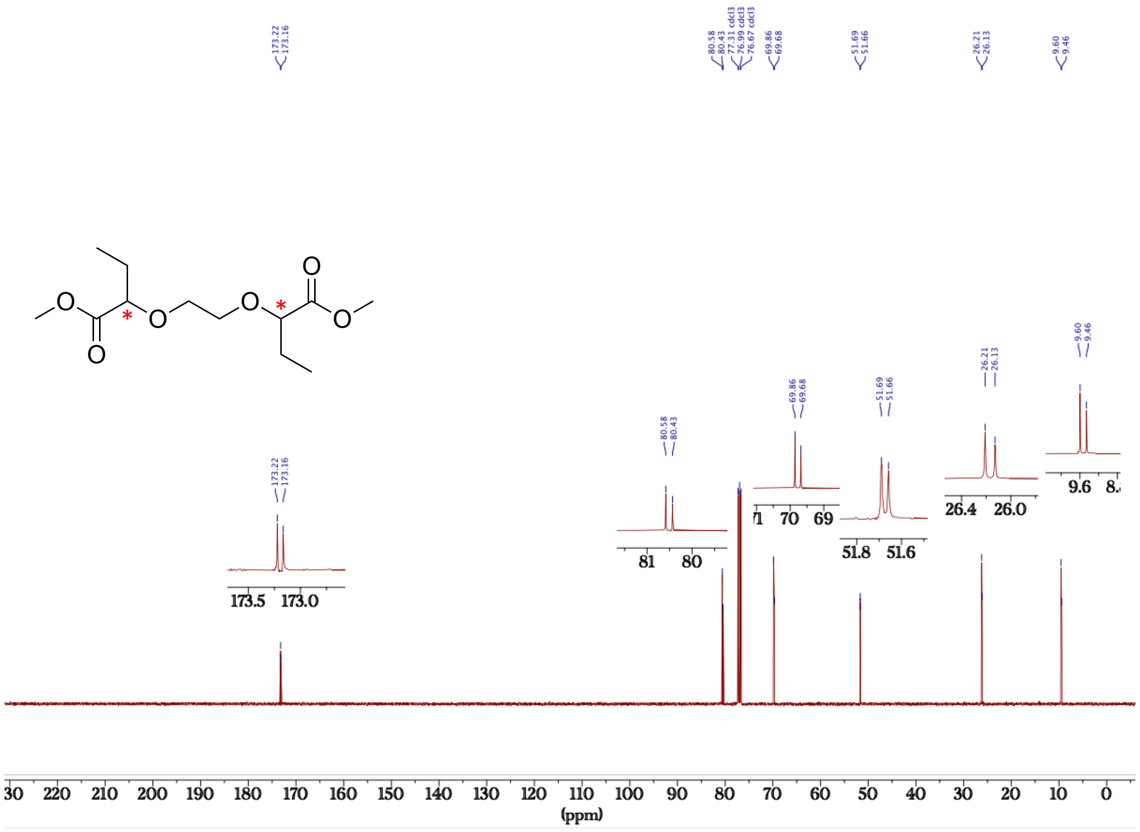

Figure S1. ^{13}C NMR of Compound **17**. Each carbon signal showed two peaks instead of a single peak since the dimethyl ester produced is a mixture of the enantiomers and the meso isomer.

Compound 18

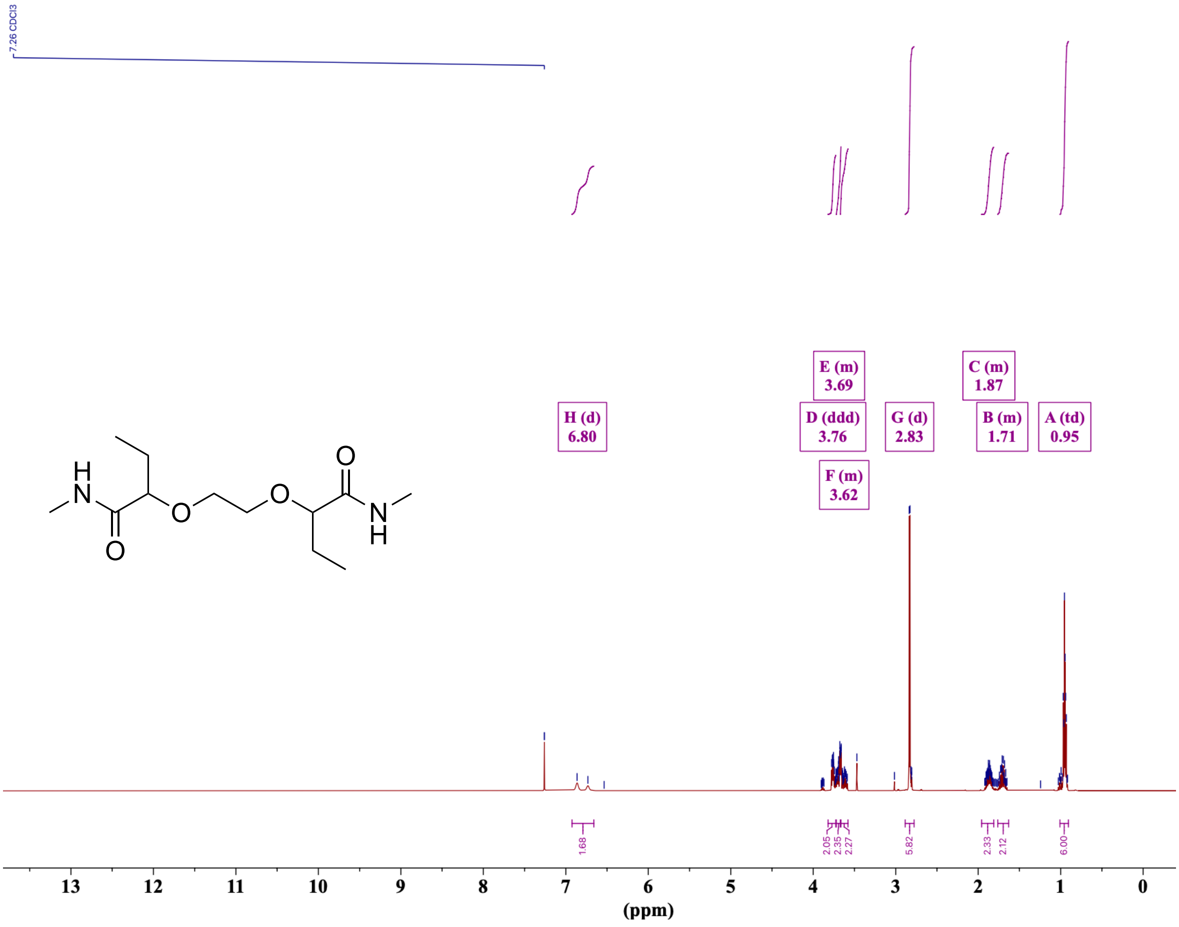

7.26 CDCl3
E (m)
3.69
C (m)
1.87
H (d)
6.80
D (ddd)
3.76
G (d)
2.83
B (m)
1.71
A (td)
0.95
F (m)
3.62
1.68
2.05
2.35
2.27
5.82
2.33
2.12
6.00
13
12
11
10
9
8
7
6
5
4
3
2
1
0
(ppm)

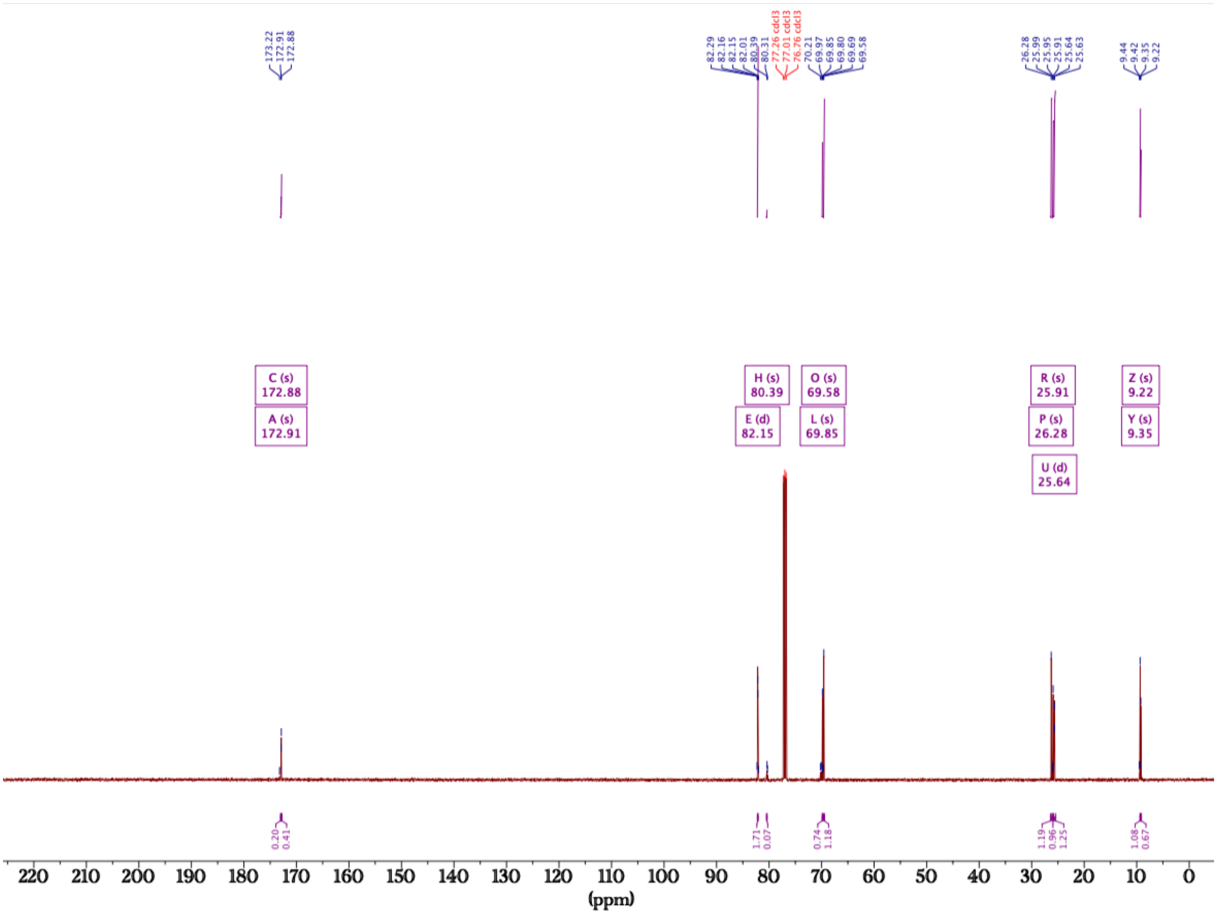

173.22
172.91
172.88
82.29
82.16
82.15
82.01
80.39
80.31
77.26 cdcl3
77.01 cdcl3
76.76 cdcl3
70.21
69.97
69.85
69.80
69.69
69.58
26.28
25.99
25.95
25.91
25.64
25.63
9.44
9.42
9.35
9.22
C (s) 172.88
A (s) 172.91
H (s) 80.39
E (d) 82.15
O (s) 69.58
L (s) 69.85
R (s) 25.91
P (s) 26.28
U (d) 25.64
Z (s) 9.22
Y (s) 9.35
0.20
0.41
1.71
0.07
0.74
1.18
1.19
0.96
1.25
1.08
0.67
220 210 200 190 180 170 160 150 140 130 120 110 100 90 80 70 60 50 40 30 20 10 0
(ppm)

Compound 19

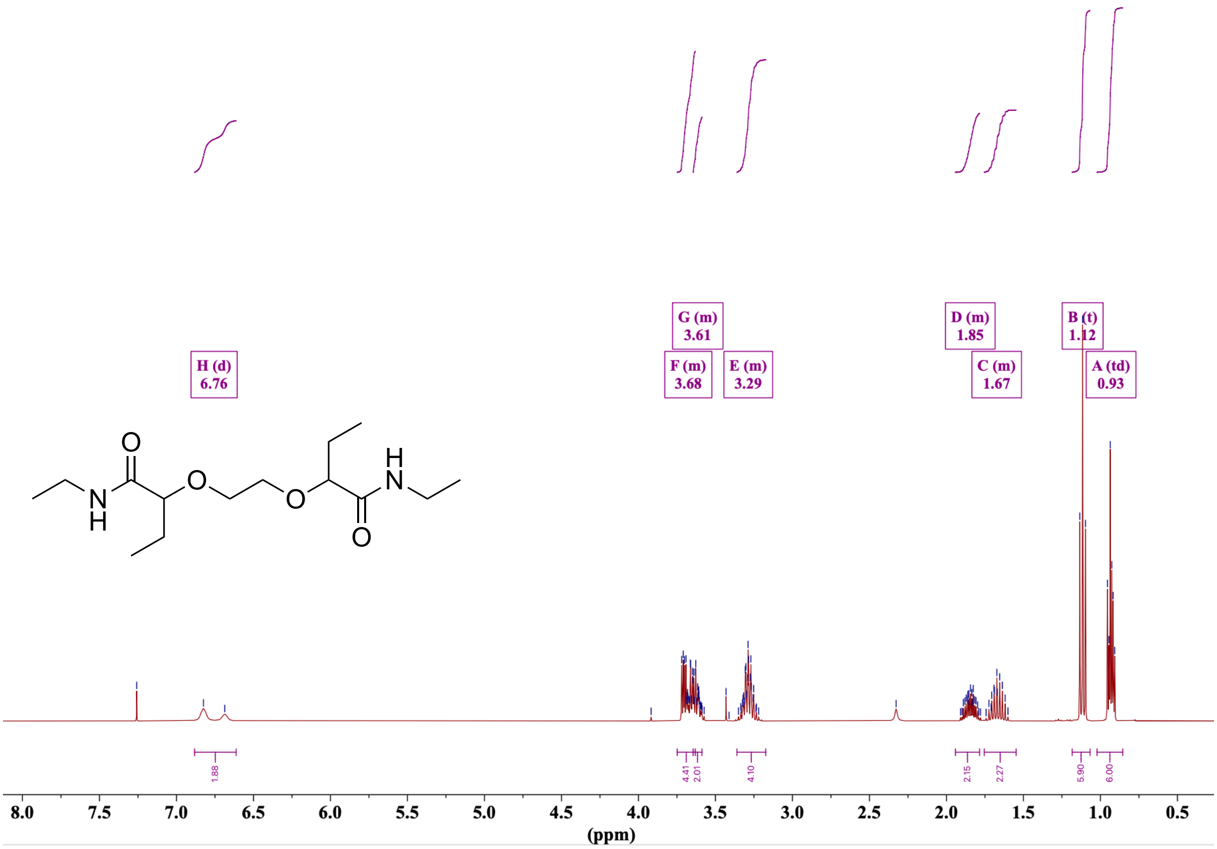

H (d)
6.76
G (m)
3.61
F (m)
3.68
E (m)
3.29
D (m)
1.85
C (m)
1.67
B (t)
1.12
A (td)
0.93
1.88
4.41
2.01
4.10
2.15
2.27
5.90
6.00
8.0
7.5
7.0
6.5
6.0
5.5
5.0
4.5
4.0
3.5
3.0
2.5
2.0
1.5
1.0
0.5
(ppm)

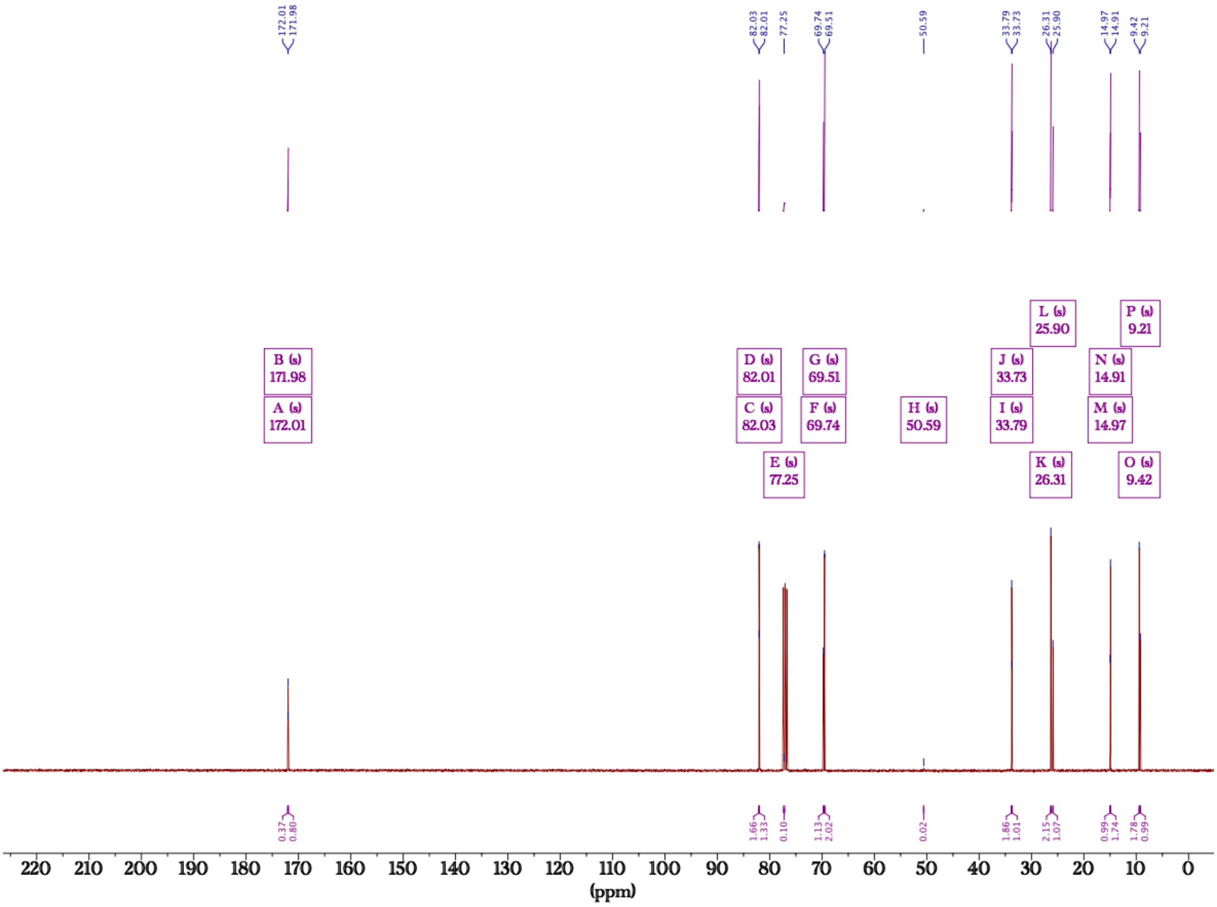

172.01
171.98
82.03
82.01
77.25
69.74
69.51
50.59
33.79
33.73
26.31
25.90
14.97
14.91
9.42
9.21
B (s)
171.98
A (s)
172.01
D (s)
82.01
C (s)
82.03
E (s)
77.25
G (s)
69.51
F (s)
69.74
H (s)
50.59
J (s)
33.73
I (s)
33.79
L (s)
25.90
K (s)
26.31
N (s)
14.91
M (s)
14.97
P (s)
9.21
O (s)
9.42
0.37
0.80
1.66
1.33
0.10
1.13
2.02
0.02
1.86
1.01
2.15
1.07
0.99
1.74
1.78
0.99
220
210
200
190
180
170
160
150
140
130
120
110
100
90
80
70
60
50
40
30
20
10
0
(ppm)

Compound 20

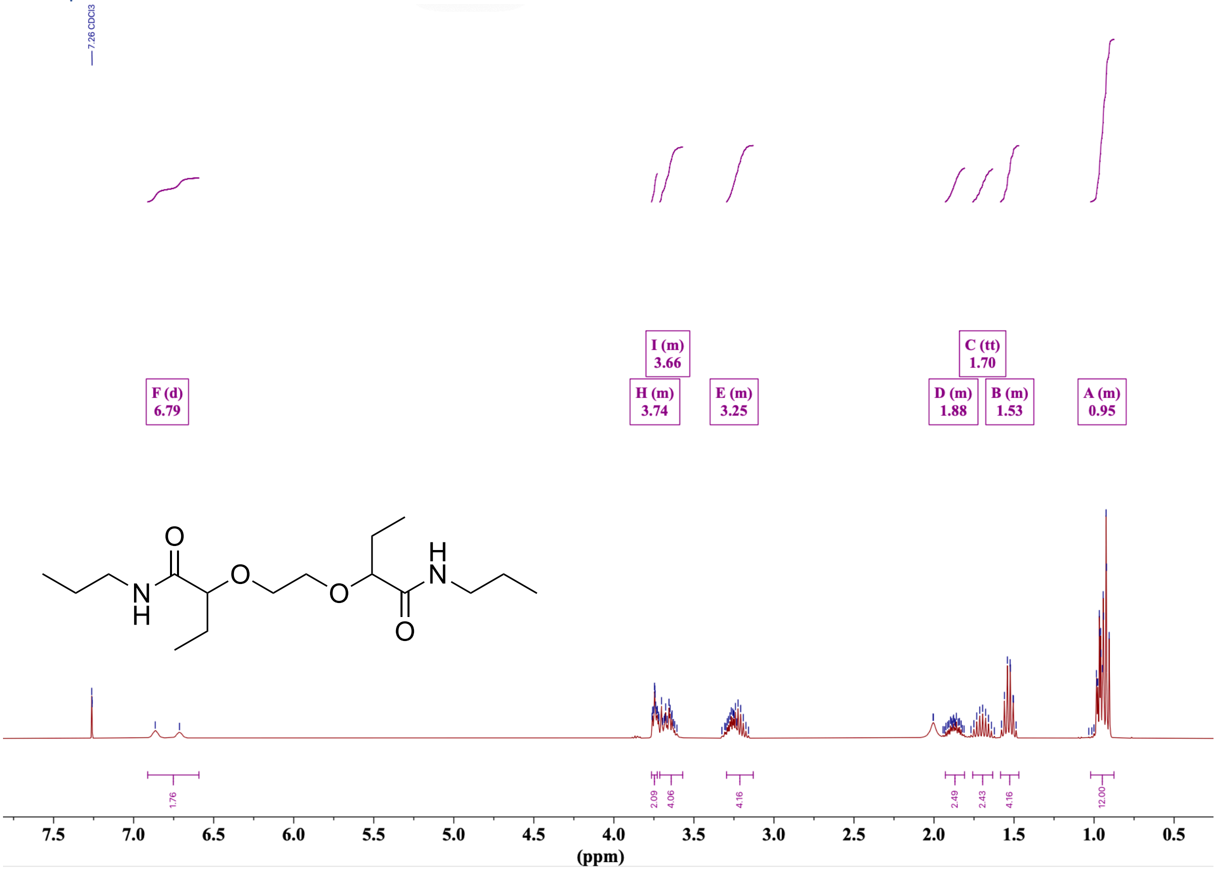

7.26 CDCl3
I (m)
3.66
C (tt)
1.70
F (d)
6.79
H (m)
3.74
E (m)
3.25
D (m)
1.88
B (m)
1.53
A (m)
0.95
1.76
2.09
4.06
4.16
2.49
2.43
4.16
12.00
7.5
7.0
6.5
6.0
5.5
5.0
4.5
4.0
3.5
3.0
2.5
2.0
1.5
1.0
0.5
(ppm)

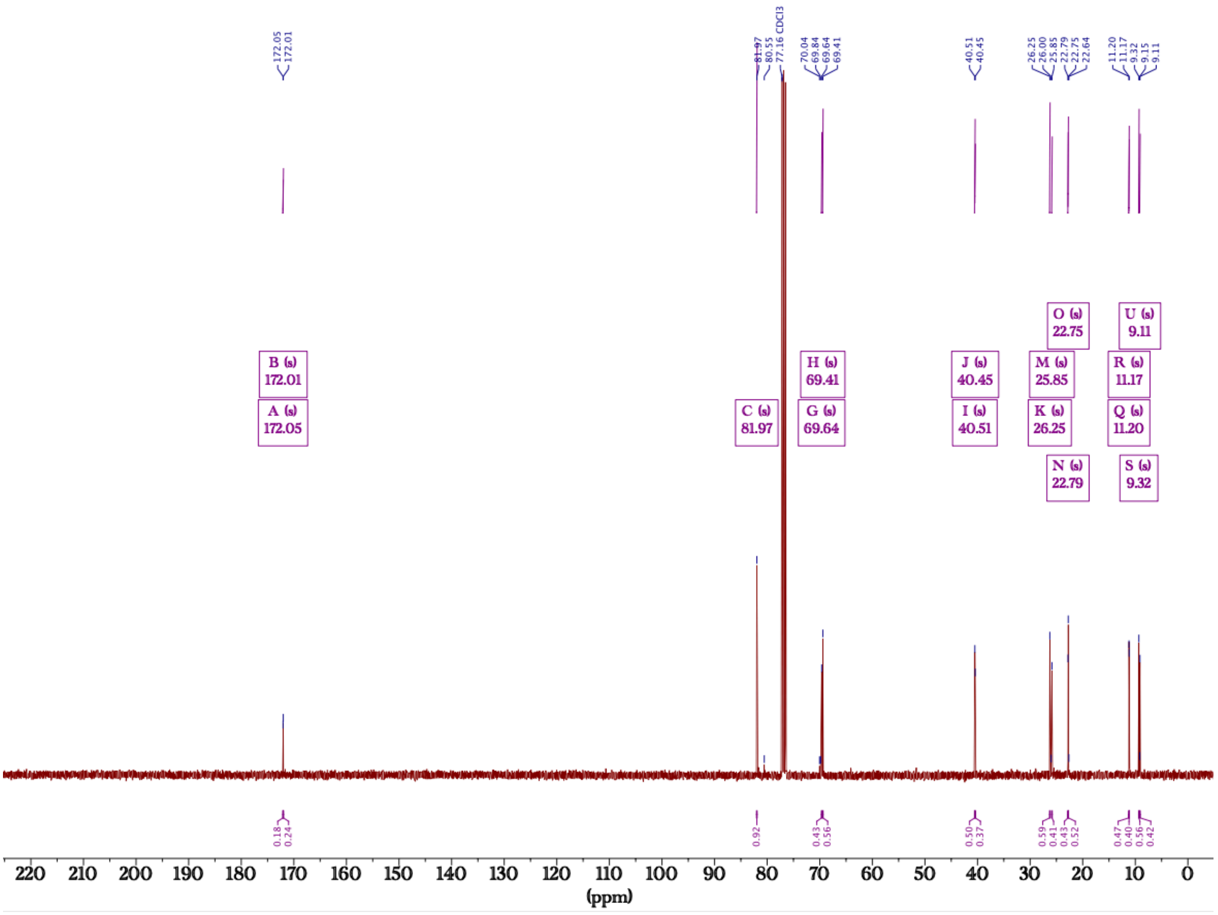
172.05
172.01
81.97
80.55
77.16 CDCl3
70.04
69.84
69.64
69.41
40.51
40.45
26.25
26.00
25.85
22.79
22.75
22.64
11.20
11.17
9.32
9.15
9.11
B (s)
172.01
A (s)
172.05
C (s)
81.97
H (s)
69.41
G (s)
69.64
J (s)
40.45
I (s)
40.51
O (s)
22.75
M (s)
25.85
K (s)
26.25
N (s)
22.79
U (s)
9.11
R (s)
11.17
Q (s)
11.20
S (s)
9.32
0.18
0.24
0.92
0.43
0.56
0.50
0.37
0.59
0.41
0.43
0.52
0.47
0.40
0.56
0.42
220 210 200 190 180 170 160 150 140 130 120 110 100 90 80 70 60 50 40 30 20 10 0
(ppm)

Compound 21

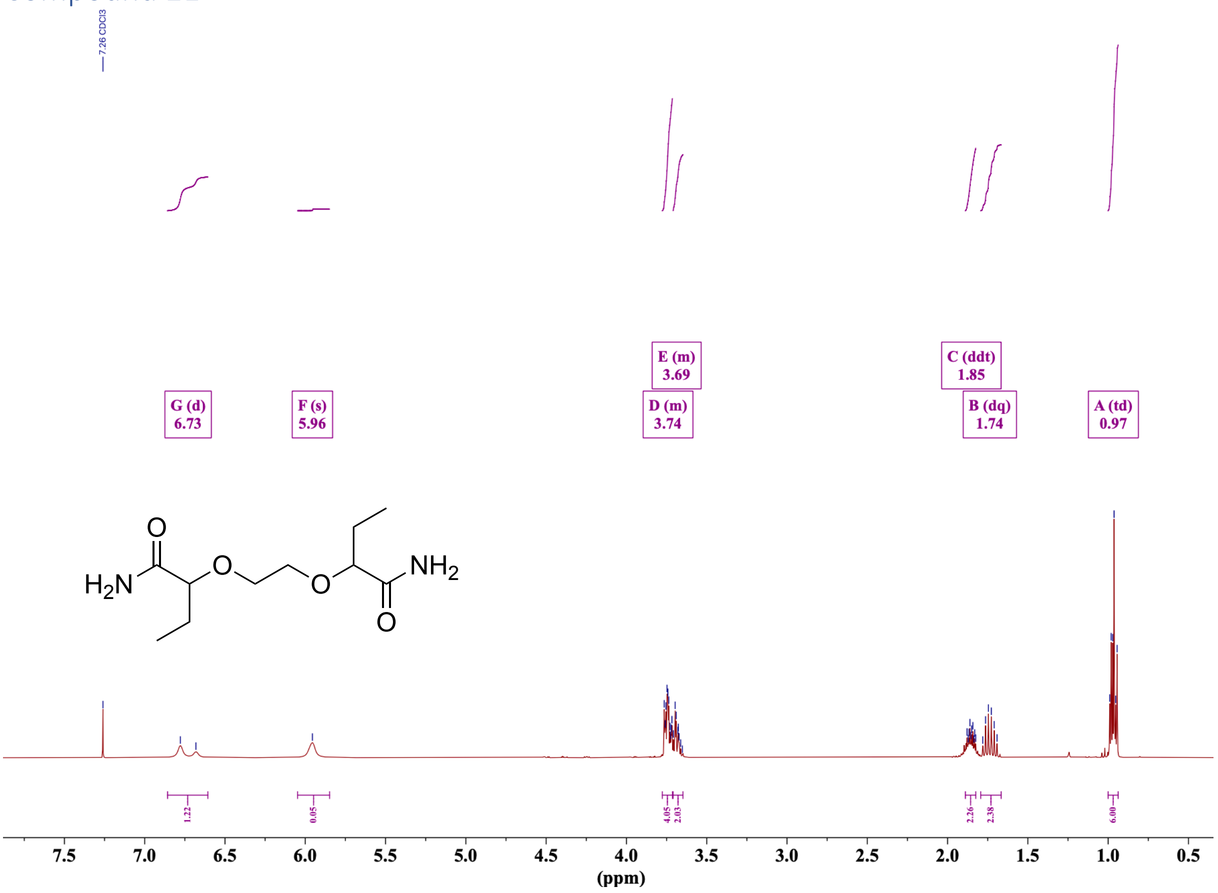
7.26 CDCl3
E (m)
3.69
C (ddt)
1.85
G (d)
6.73
F (s)
5.96
D (m)
3.74
B (dq)
1.74
A (td)
0.97
O
H_2N
O
O
NH_2
O
1.22
0.05
4.05
2.03
2.26
2.38
6.00
7.5
7.0
6.5
6.0
5.5
5.0
4.5
4.0
3.5
3.0
2.5
2.0
1.5
1.0
0.5
(ppm)

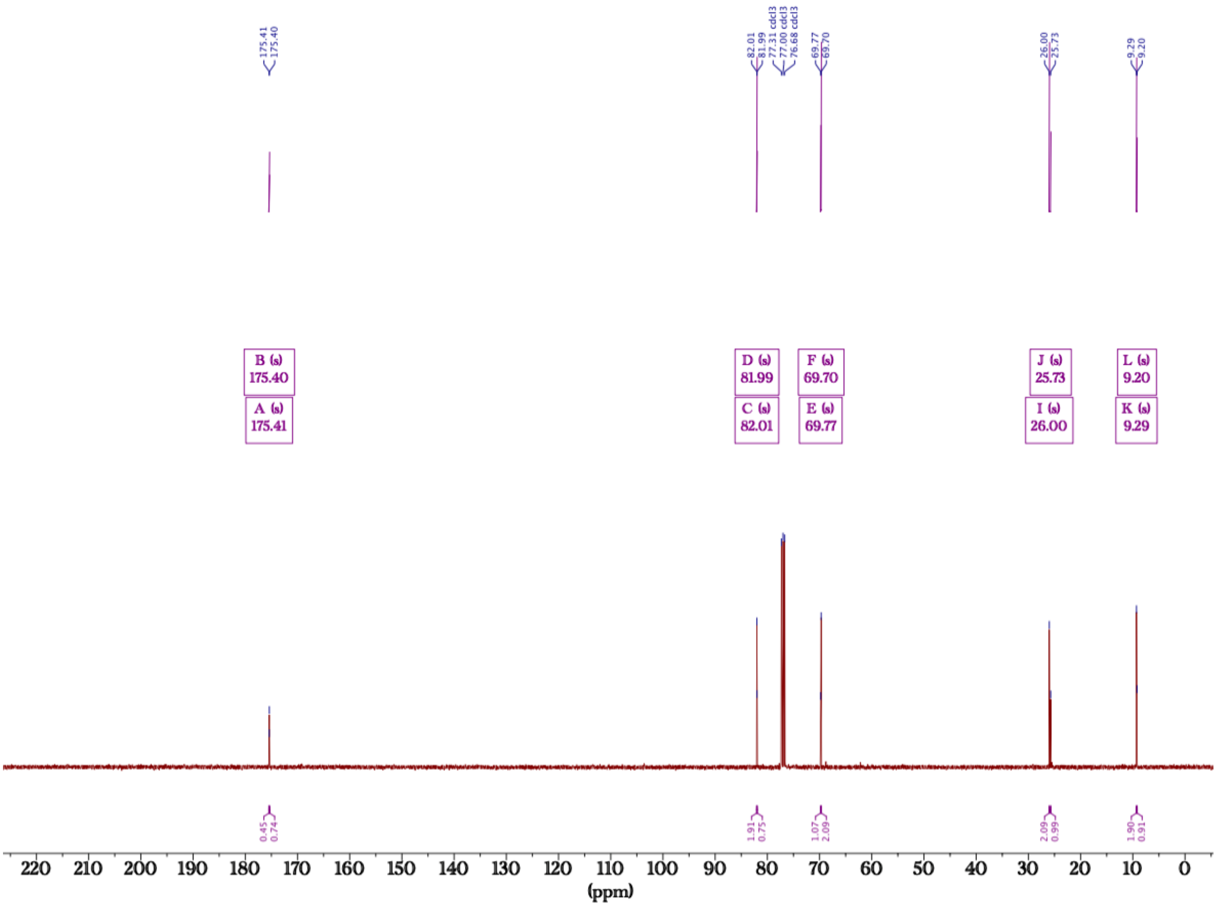
175.41
175.40
82.01
81.99
77.31 cdcl3
77.00 cdcl3
76.68 cdcl3
69.77
69.70
26.00
25.73
9.29
9.20
B (s)
175.40
A (s)
175.41
D (s)
81.99
C (s)
82.01
F (s)
69.70
E (s)
69.77
J (s)
25.73
I (s)
26.00
L (s)
9.20
K (s)
9.29
0.45
0.74
1.91
0.75
1.07
2.09
2.09
0.99
1.90
0.91
220
210
200
190
180
170
160
150
140
130
120
110
100
90
80
70
60
50
40
30
20
10
0
(ppm)

Compound 22

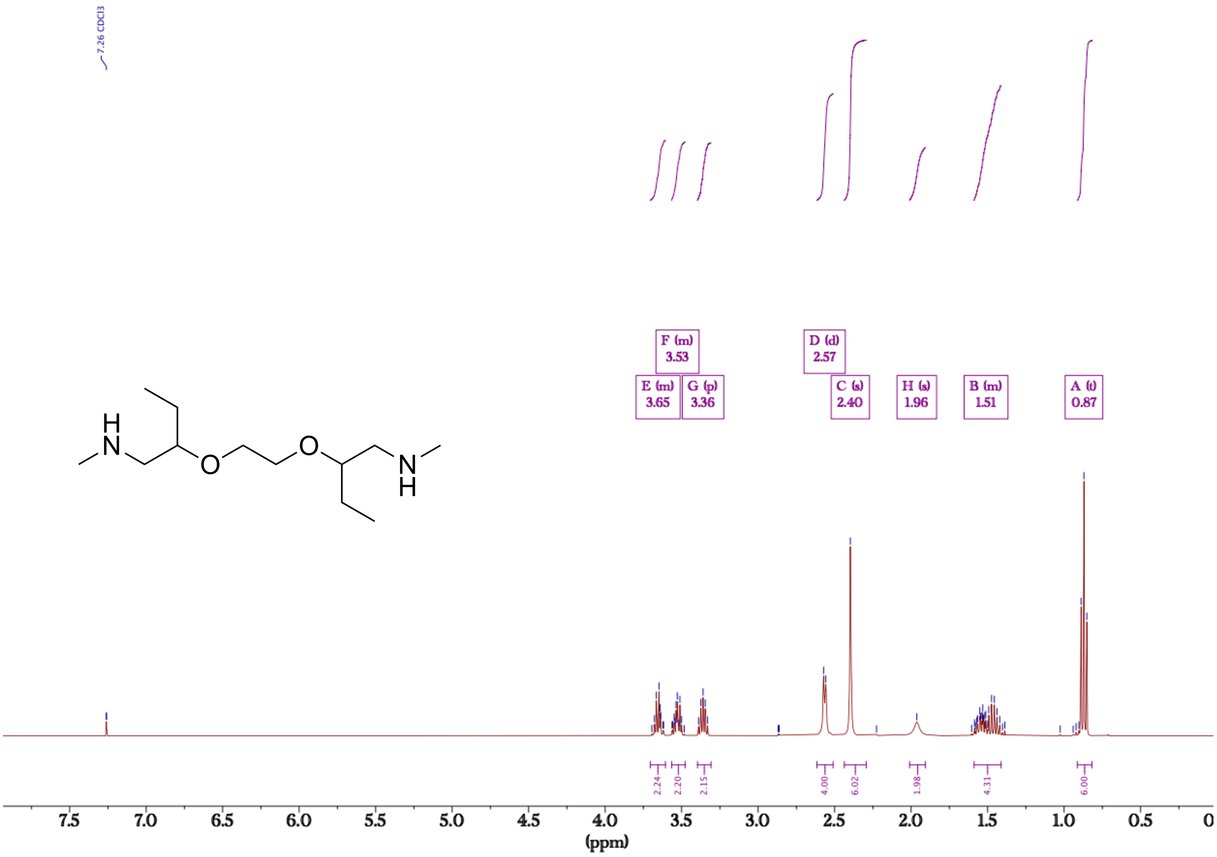

7.26 CDCl3
F (m) 3.53
D (d) 2.57
E (m) 3.65
G (p) 3.36
C (s) 2.40
H (s) 1.96
B (m) 1.51
A (t) 0.87
2.24
2.20
2.15
4.00
6.02
1.98
4.31
6.00
(ppm)

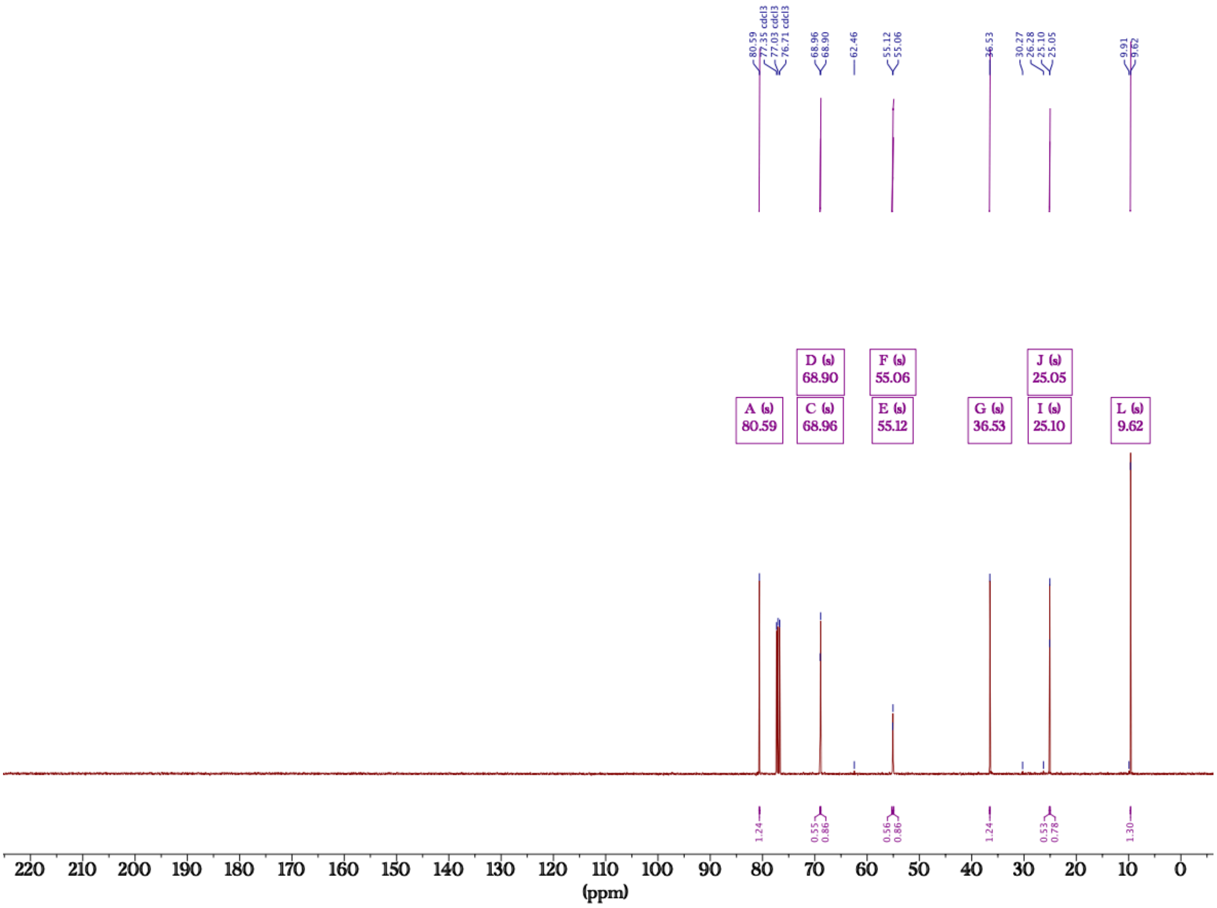

80.59
77.35 cdcl3
77.03 cdcl3
76.71 cdcl3
68.96
68.90
62.46
55.12
55.06
36.53
30.27
26.28
25.10
25.05
9.91
9.62
D (s) 68.90
F (s) 55.06
J (s) 25.05
A (s) 80.59
C (s) 68.96
E (s) 55.12
G (s) 36.53
I (s) 25.10
L (s) 9.62
(ppm)

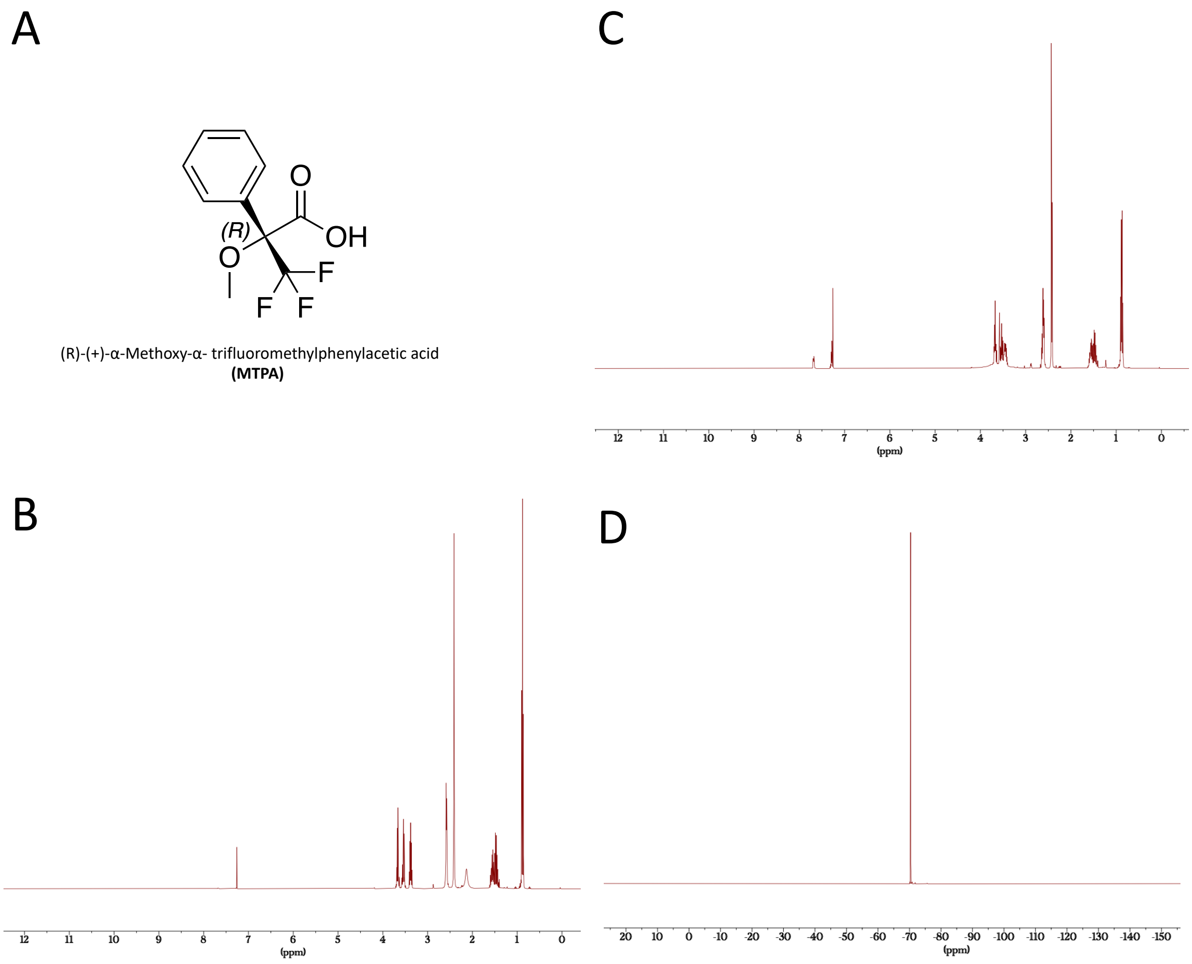

Figure S2. (A) Mosher's acid (MTPA). **(B)** ^{1}H NMR of **22** with 0.16 equivalents MTPA. **(C)** ^{1}H NMR of **22** with 2 equivalents of MTPA. **(D)** ^{19}F NMR of **22** with 2 equivalents MTPA.

Compound 23

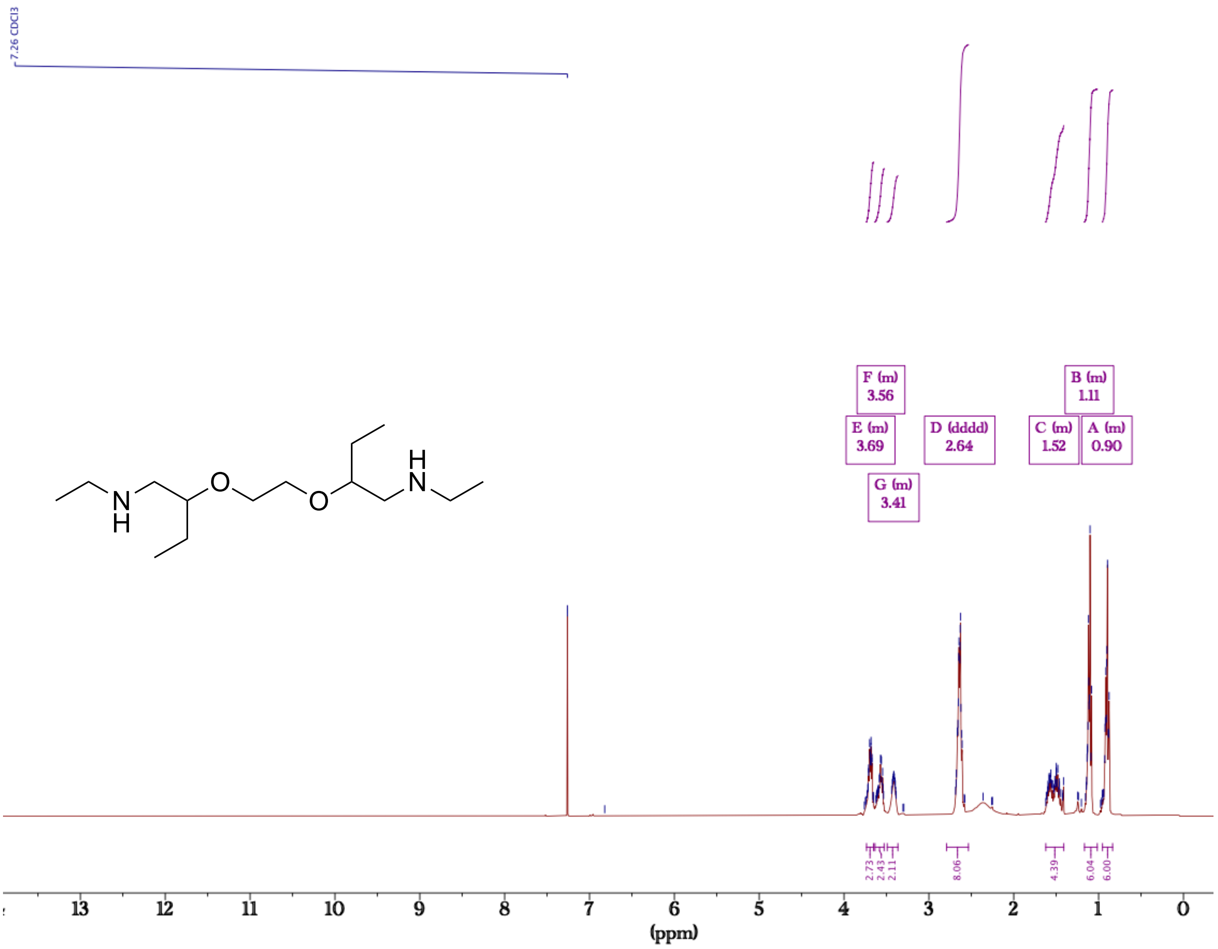
7.26 CDCl3
F (m)
3.56
E (m)
3.69
D (dddd)
2.64
G (m)
3.41
B (m)
1.11
C (m)
1.52
A (m)
0.90
2.73
2.43
2.11
8.06
4.39
6.04
6.00
13
12
11
10
9
8
7
6
5
4
3
2
1
0
(ppm)

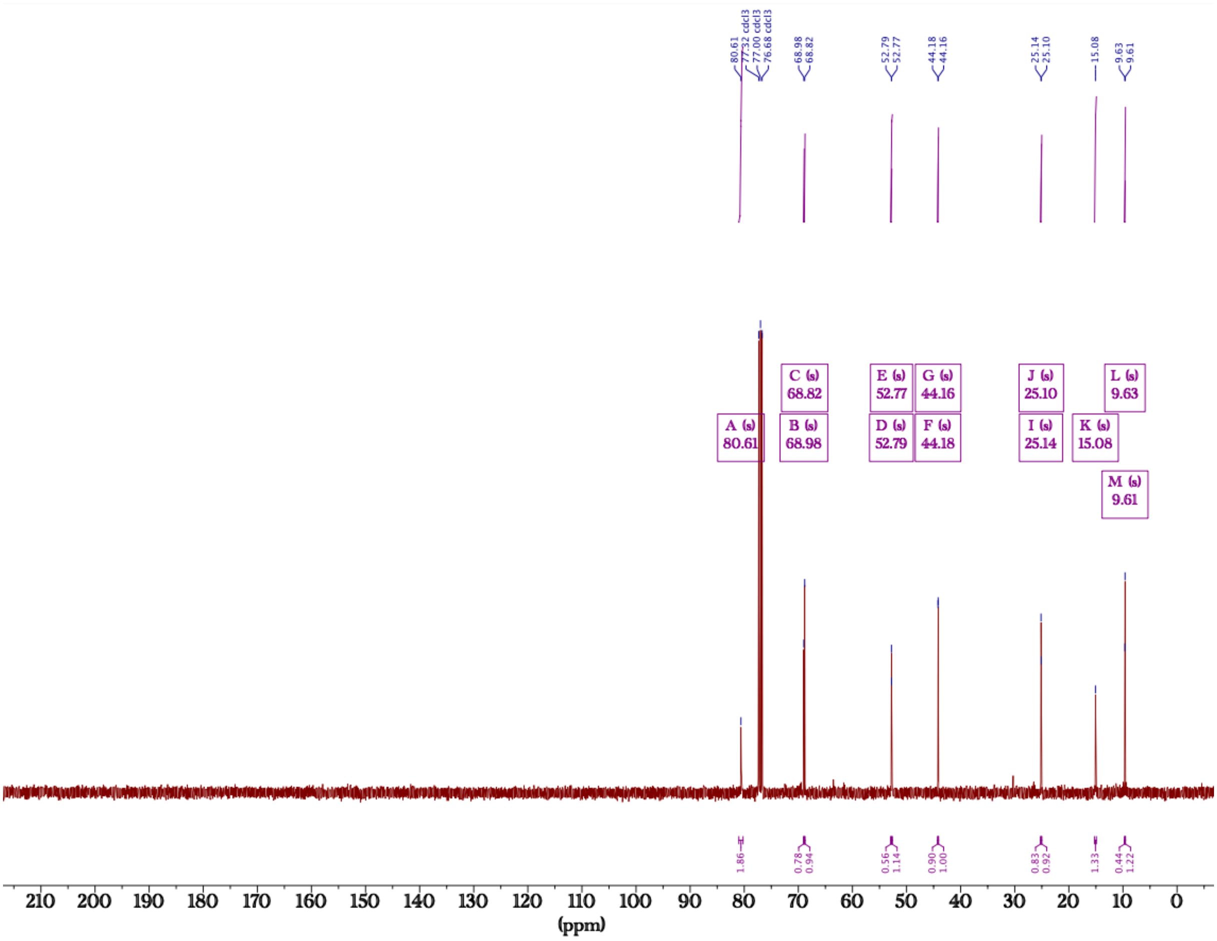

80.61
77.32 cdcl3
77.00 cdcl3
76.68 cdcl3
68.98
68.82
52.79
52.77
44.18
44.16
25.14
25.10
15.08
9.63
9.61
A (s) 80.61
B (s) 68.98
C (s) 68.82
D (s) 52.79
E (s) 52.77
F (s) 44.18
G (s) 44.16
I (s) 25.14
J (s) 25.10
K (s) 15.08
L (s) 9.63
M (s) 9.61
1.86
0.78
0.94
0.56
1.14
0.90
1.00
0.83
0.92
1.33
0.44
1.22
210 200 190 180 170 160 150 140 130 120 110 100 90 80 70 60 50 40 30 20 10 0
(ppm)

Compound 24

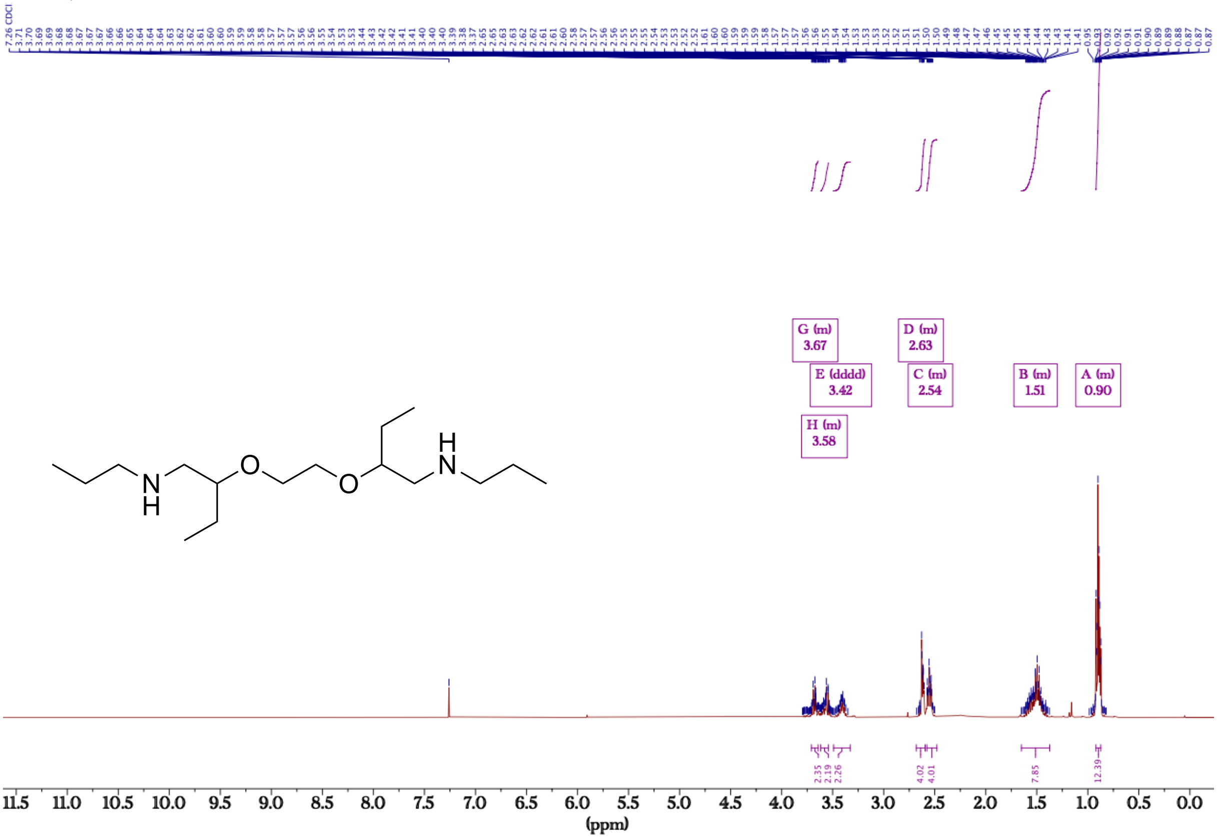

N
H
O
O
H
N
G (m) 3.67
D (m) 2.63
E (dddd) 3.42
C (m) 2.54
B (m) 1.51
A (m) 0.90
H (m) 3.58
(ppm)

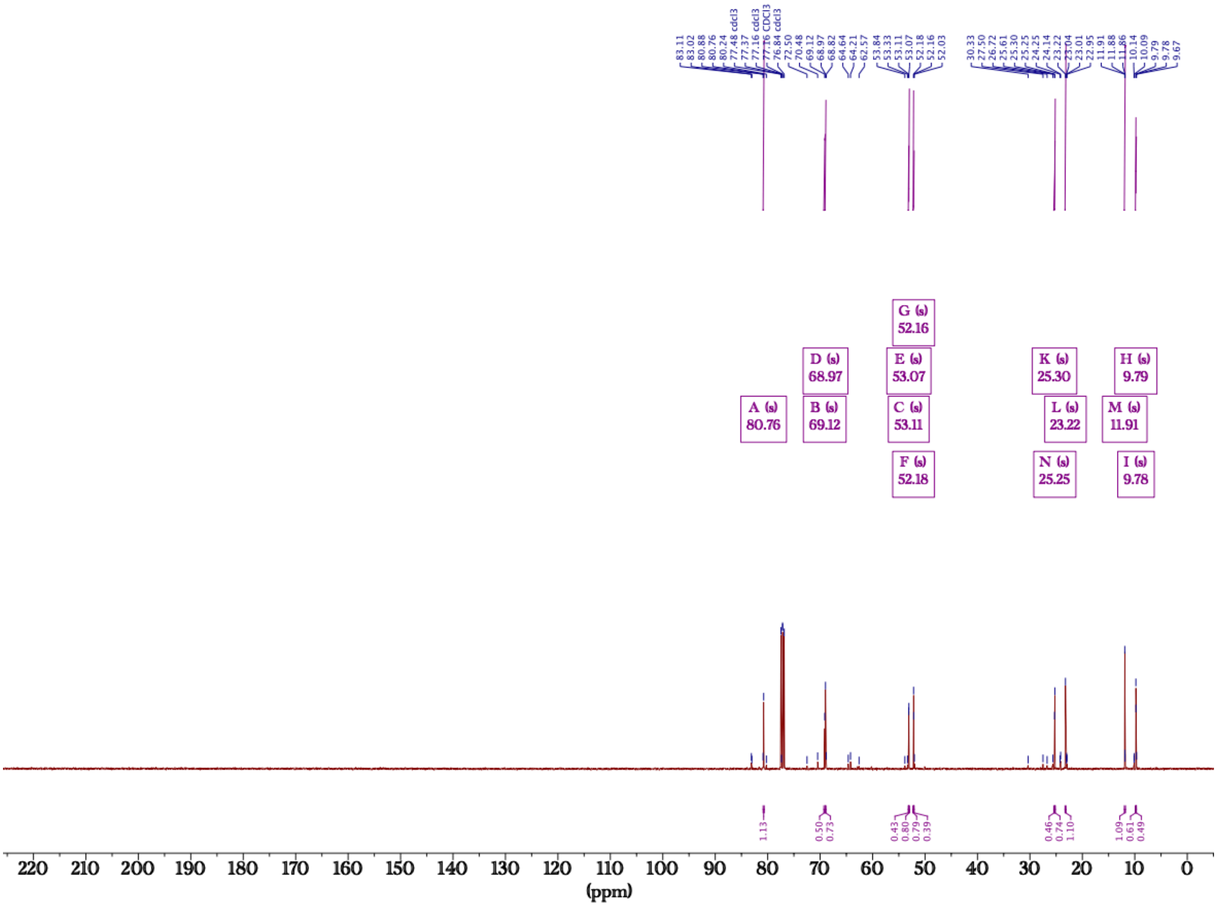

G (s) 52.16
D (s) 68.97
E (s) 53.07
K (s) 25.30
H (s) 9.79
A (s) 80.76
B (s) 69.12
C (s) 53.11
L (s) 23.22
M (s) 11.91
F (s) 52.18
N (s) 25.25
I (s) 9.78
(ppm)

Compound 25

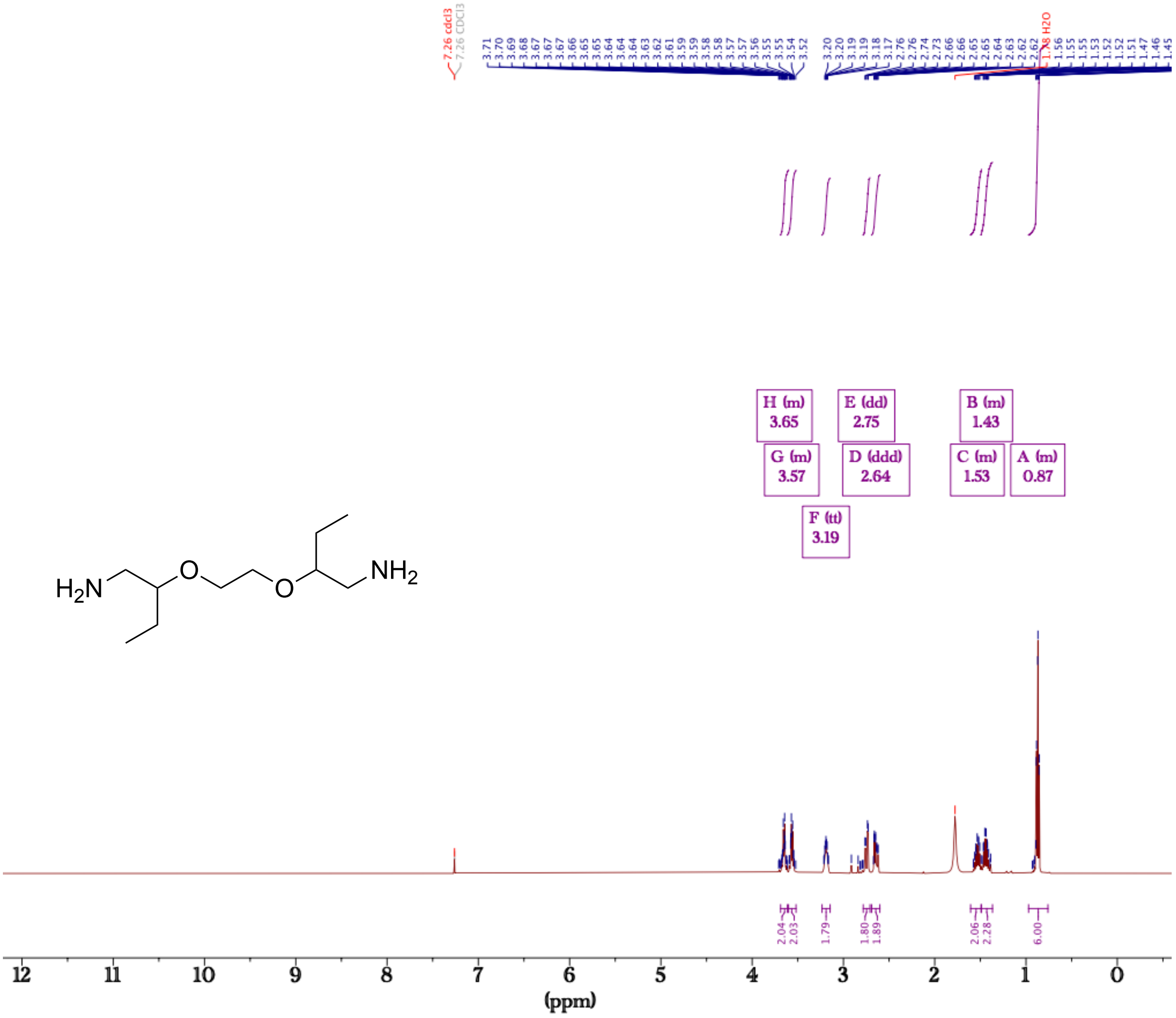

7.26 cdcl3
7.26 CDCl3
3.71
3.70
3.69
3.68
3.67
3.67
3.67
3.66
3.65
3.65
3.64
3.64
3.64
3.63
3.62
3.62
3.61
3.59
3.59
3.58
3.58
3.57
3.57
3.56
3.55
3.55
3.55
3.54
3.52
3.20
3.20
3.19
3.19
3.18
3.17
2.76
2.76
2.74
2.73
2.66
2.66
2.65
2.65
2.64
2.63
2.62
2.62
1.78 H2O
1.56
1.55
1.55
1.53
1.52
1.52
1.51
1.47
1.46
1.45
H (m)
3.65
E (dd)
2.75
B (m)
1.43
G (m)
3.57
D (ddd)
2.64
C (m)
1.53
A (m)
0.87
F (tt)
3.19
H_2N
O
O
NH_2
2.04
2.03
1.79
1.80
1.89
2.06
2.28
6.00
12
11
10
9
8
7
6
5
4
3
2
1
0
(ppm)

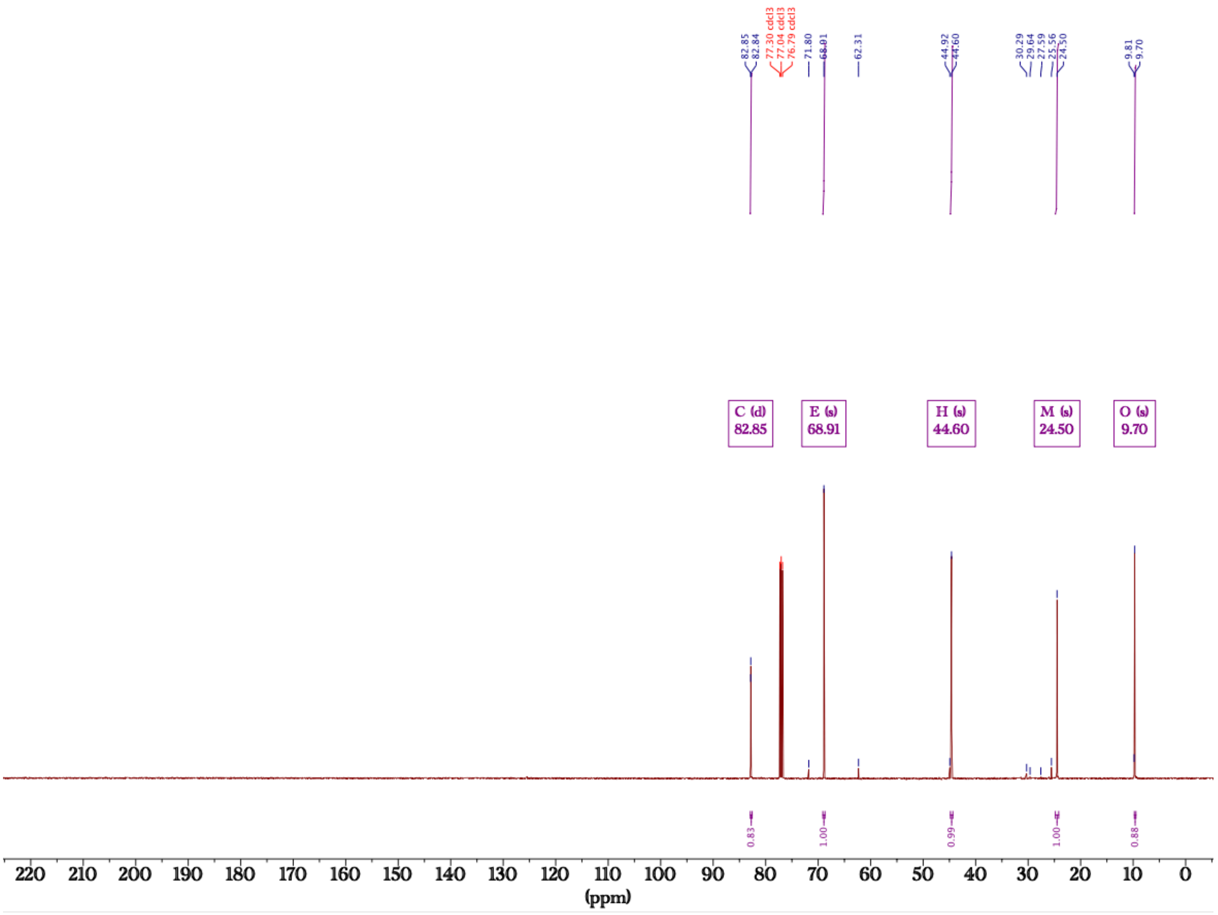

82.85
82.84
77.30 cdcl3
77.04 cdcl3
76.79 cdcl3
71.80
68.91
62.31
44.92
44.60
30.29
29.64
27.59
25.56
24.50
9.81
9.70
C (d)
82.85
E (s)
68.91
H (s)
44.60
M (s)
24.50
O (s)
9.70
0.83
1.00
0.99
1.00
0.88
220 210 200 190 180 170 160 150 140 130 120 110 100 90 80 70 60 50 40 30 20 10 0
(ppm)

HRMS

Compound 8

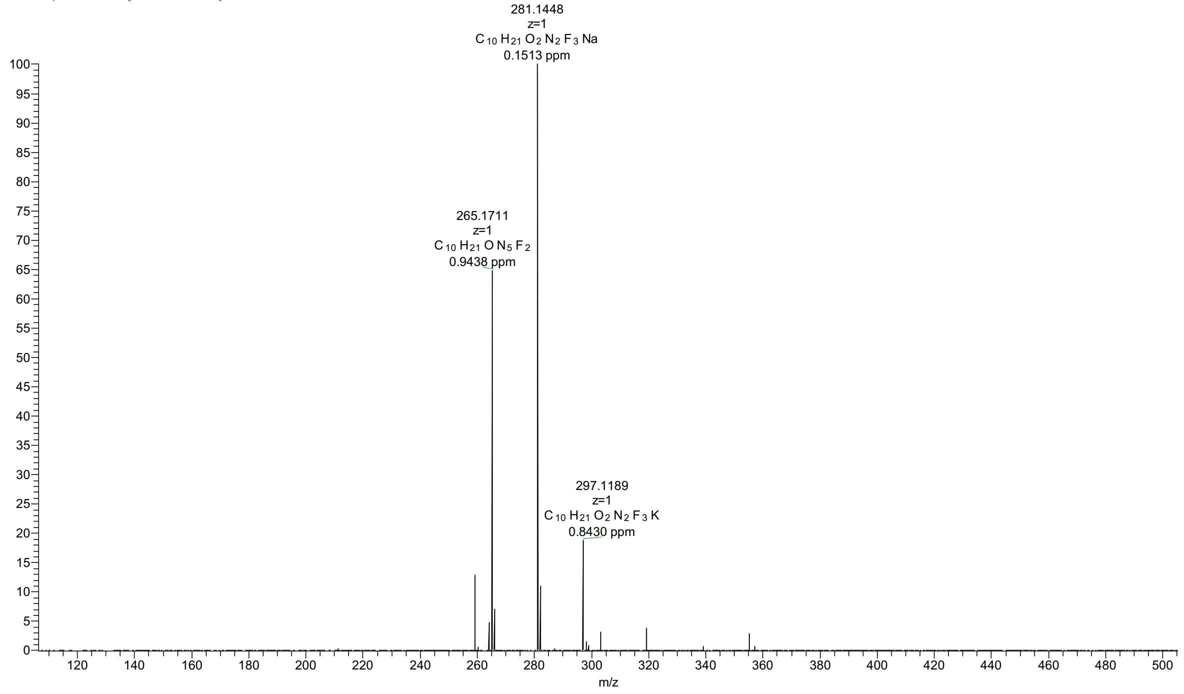

Compound 13

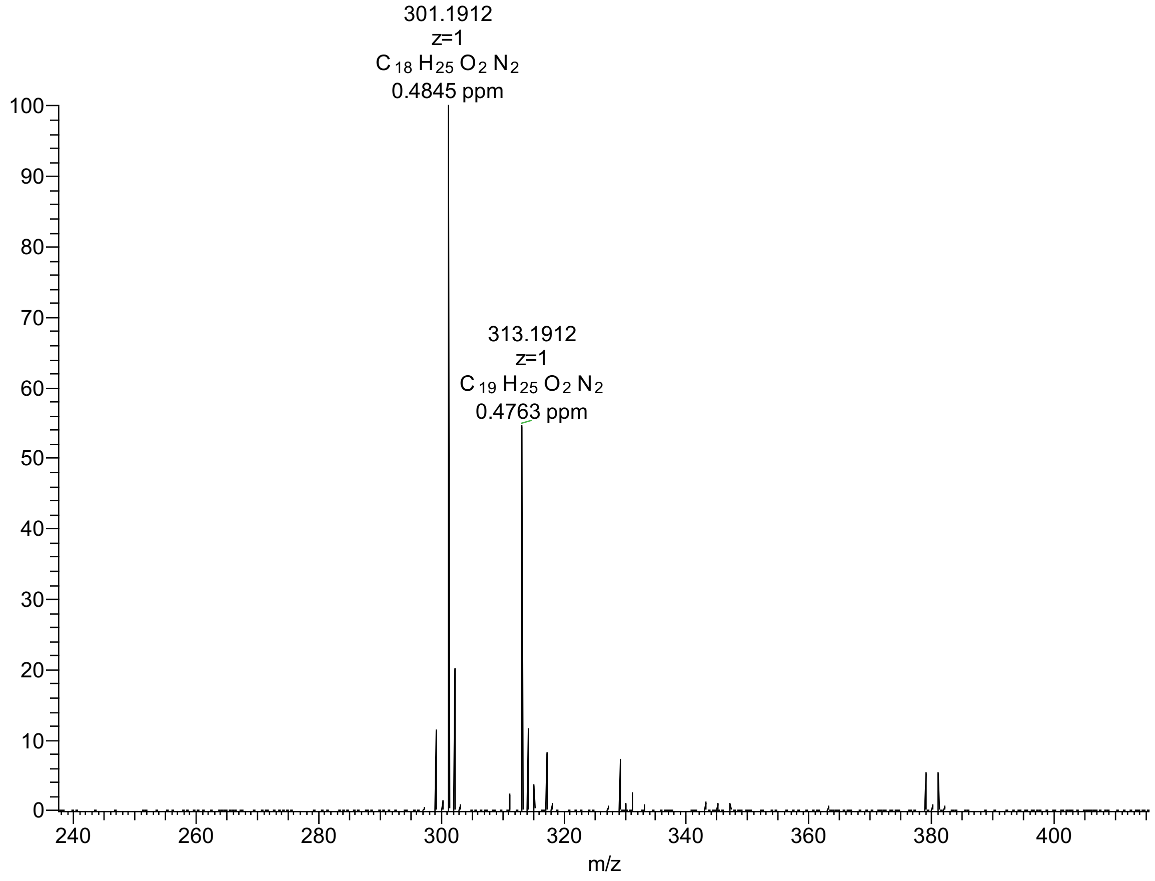

Compound 14

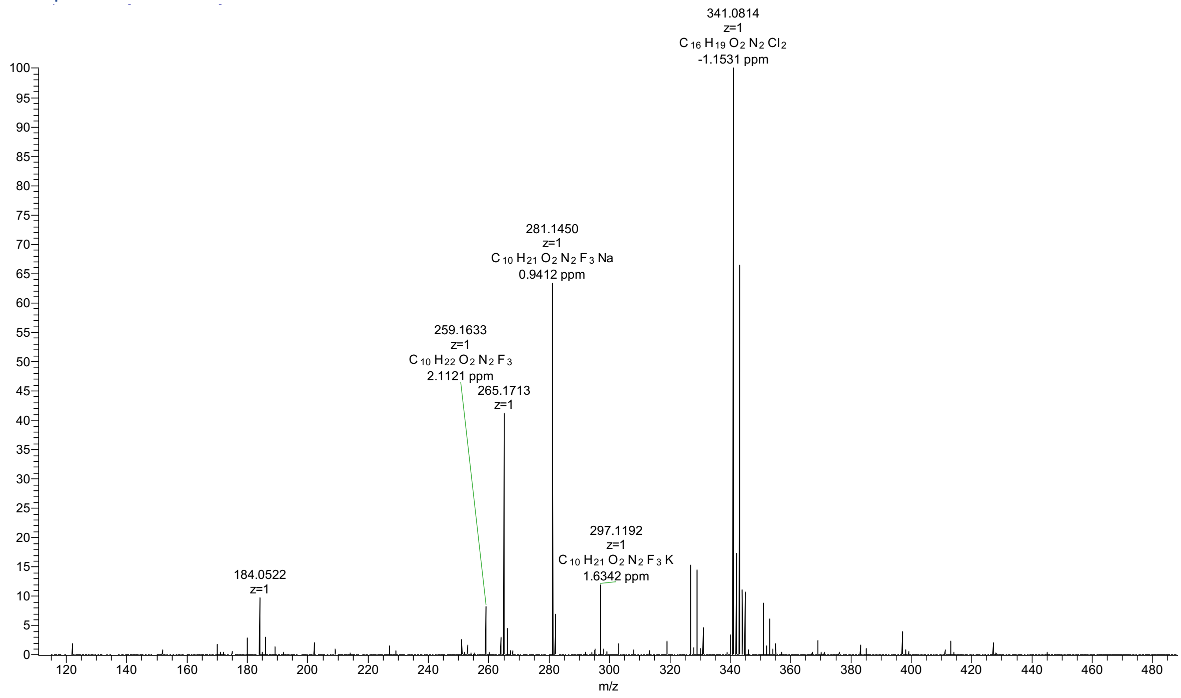

Compound 15

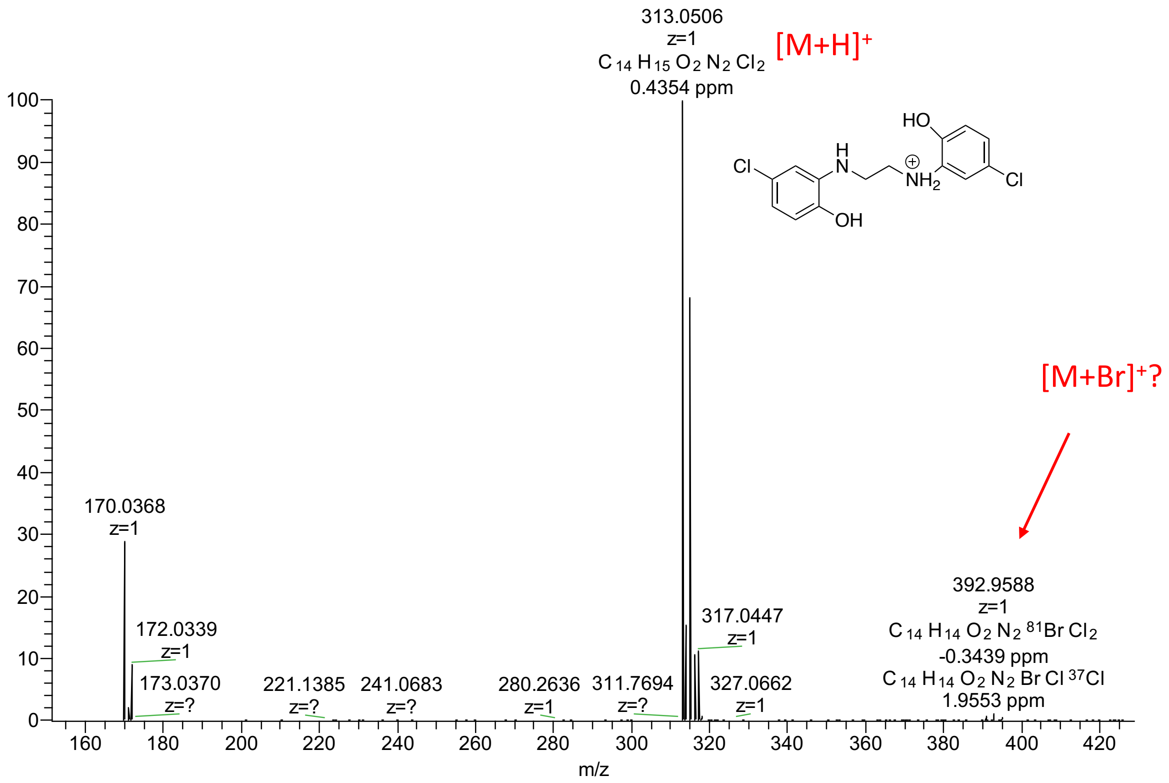

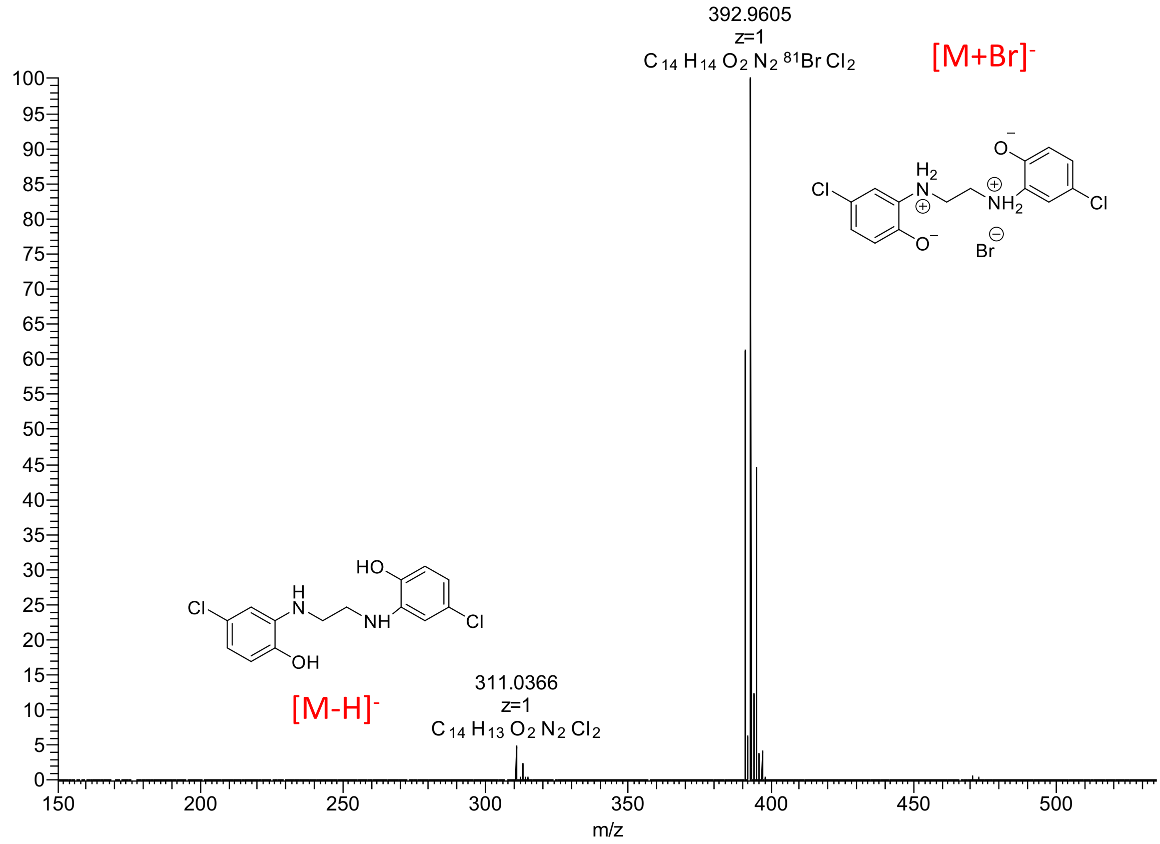

392.9605
z=1
C 14 H 14 O 2 N 2 81Br Cl 2
[M+Br]-
311.0366
z=1
C 14 H 13 O 2 N 2 Cl 2
[M-H]-
m/z

HPLC

Compound 9

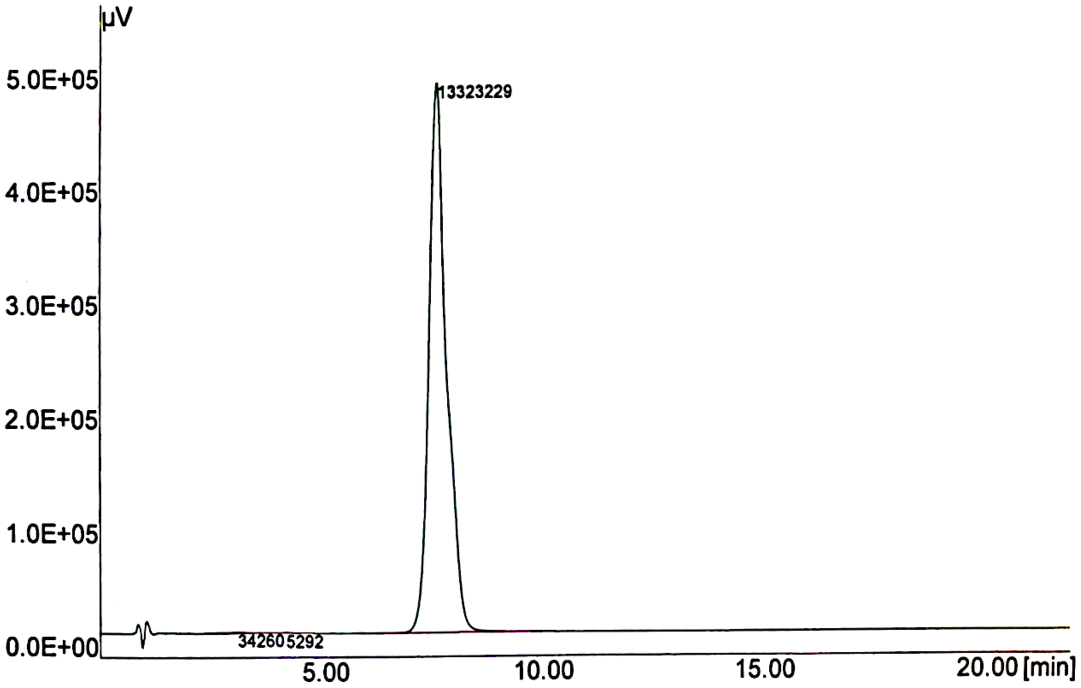

#	Name	RT	Area [µV.Sec]	Quantity
1		3.250	34259.886	0.000
2		4.333	5292.154	0.000
3		7.725	13323228.670	0.000

Total Area of Peak = 13362780.710 [µV.Sec]   % purity = 99.7%

Compound 10

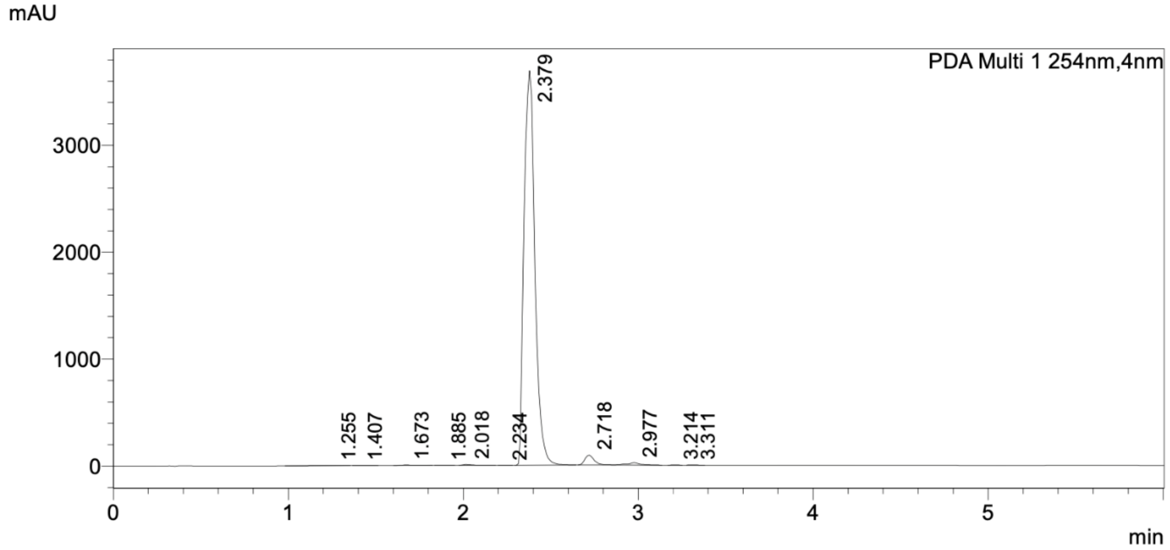

PDA Ch1 254nm

Peak#	Ret. Time	Area	Height	Area%
1	1.255	19370	751	0.118
2	1.407	2831	915	0.017
3	1.673	23621	3445	0.144
4	1.885	3607	1088	0.022
5	2.018	31355	7866	0.191
6	2.234	2854	847	0.017
7	2.379	15868370	3689354	96.786
8	2.718	333031	91251	2.031
9	2.977	101920	20766	0.622
10	3.214	2589	808	0.016
11	3.311	5702	1969	0.035
Total		16395250	3819060	100.000

Compound 11

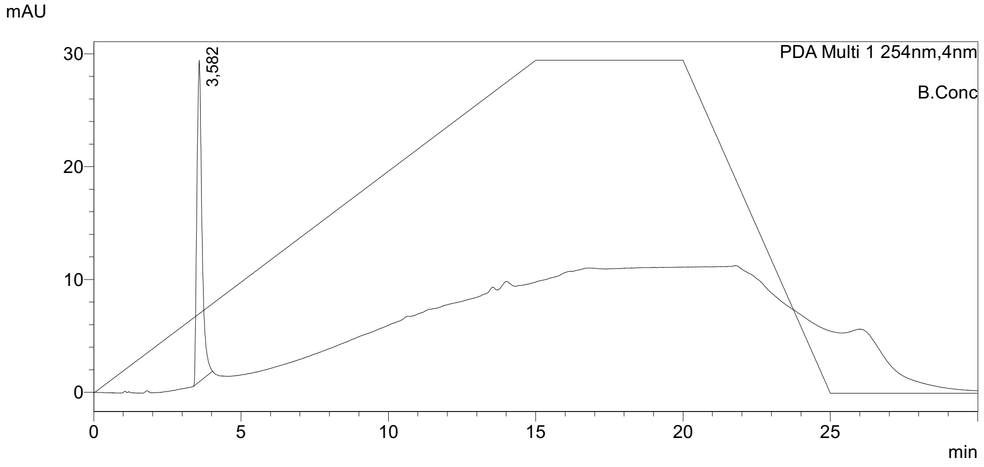

PDA Ch1 254nm

Peak#	Ret. Time	Area	Height	Area%
1	3,582	342411	28482	100,000
Total		342411	28482	100,000

Compound 12

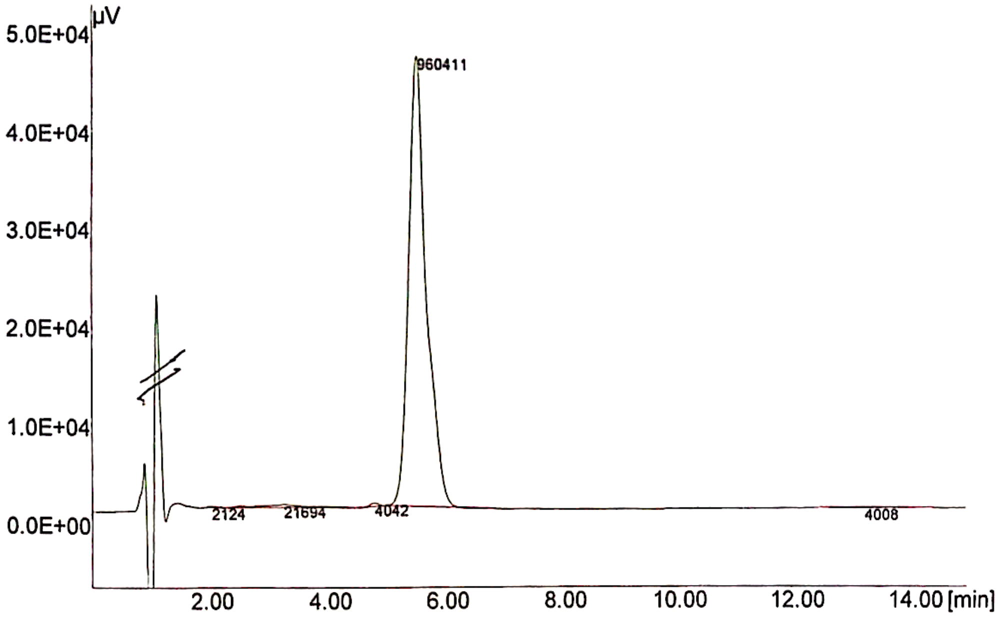

#	Name	RT	Area [µV.Sec]	Quantity
1		2.150	2124.094	0.000
2		3.367	21694.023	0.000
3		4.908	4042.148	0.000
4		5.600	960411.187	0.000
5		13.233	4008.000	0.000

Total Area of Peak = 992279.453 [µV.Sec]

% purity = 96.7%

Compound 13

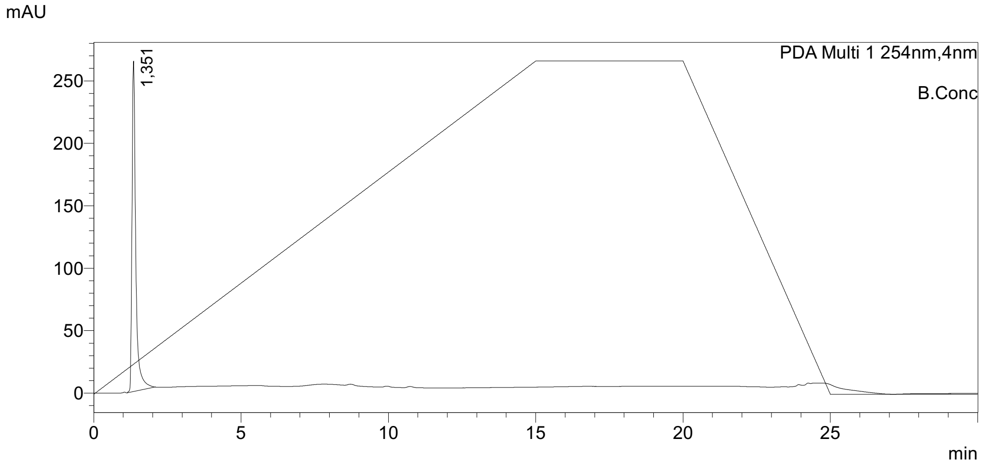

PDA Ch1 254nm

Peak#	Ret. Time	Area	Height	Area%
1	1,351	2357308	264554	100,000
Total		2357308	264554	100,000

Compound 14

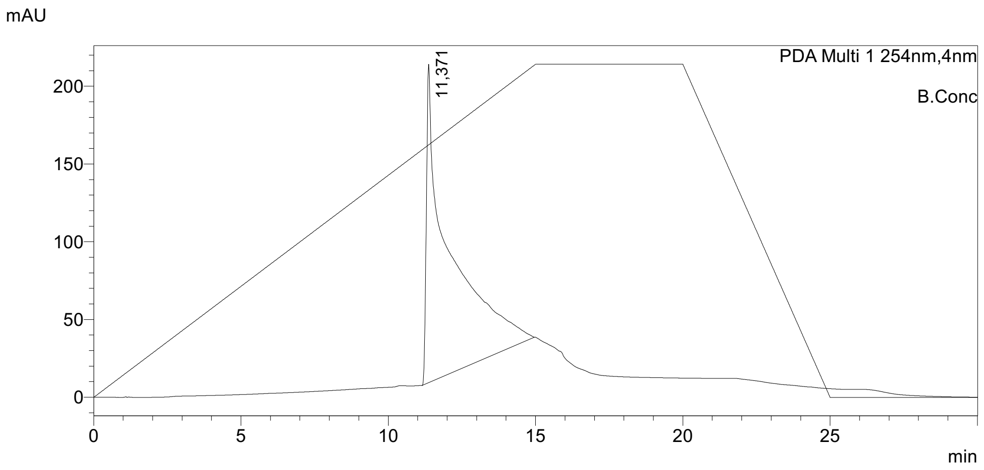

PDA Ch1 254nm

Peak#	Ret. Time	Area	Height	Area%
1	11,371	11421844	204723	100,000
Total		11421844	204723	100,000

Compound 15

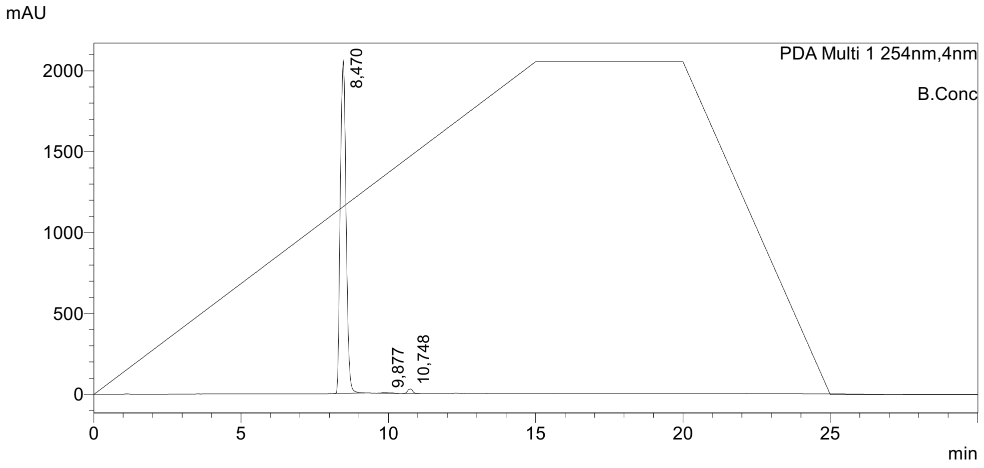

PDA Ch1 254nm

Peak#	Ret. Time	Area	Height	Area%
1	8,470	28213612	2050570	98,474
2	9,877	105142	5473	0,367
3	10,748	331929	27347	1,159
Total		28650683	2083390	100,000

Compound 18

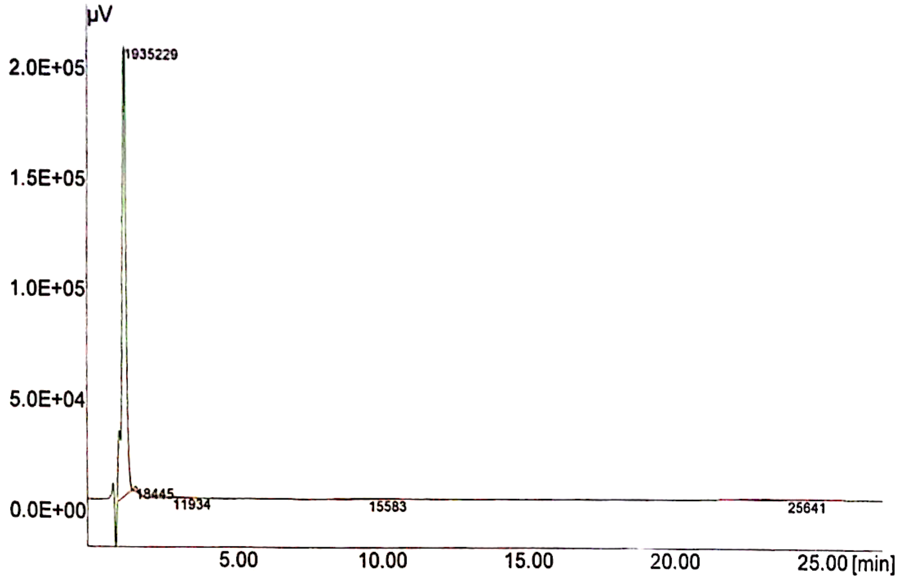

#	Name	RT	Area [µV.Sec]	Quantity
1		1.425	1935228.500	0.000
2		1.800	18445.287	0.000
3		3.058	11933.500	0.000
4		9.717	15582.662	0.000
5		23.992	25640.570	0.000

Total Area of Peak = 2006830.519 [µV.Sec]

% purity = 96.4%

Compound 19

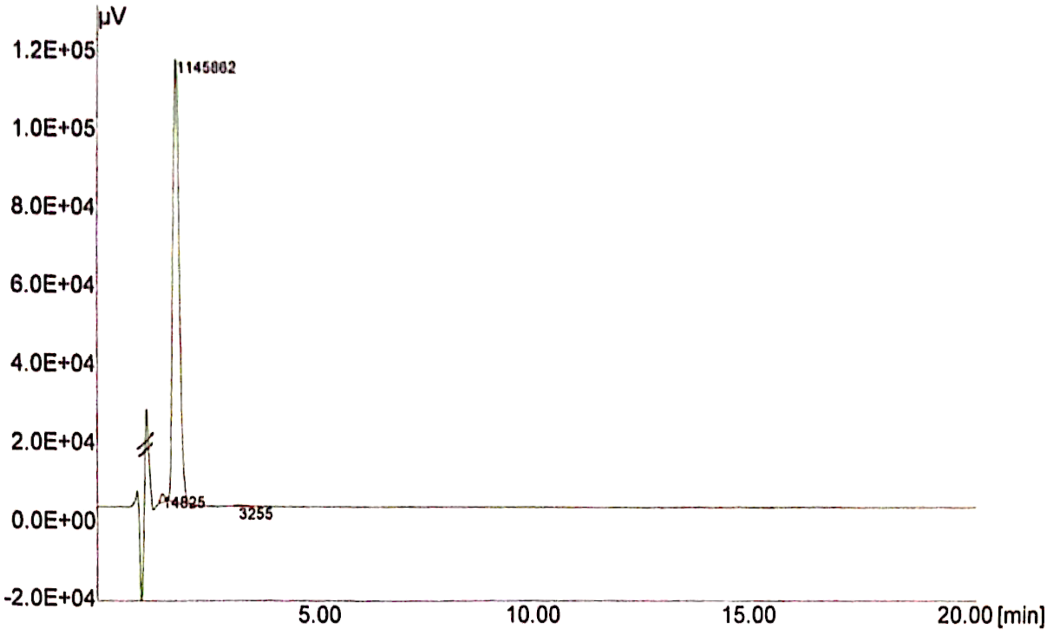

#	Name	RT	Area [μV.Sec]	Quantity
1		1.608	'14824.500	0.000
2		1.908	1145862.118	0.000
3		3.358	3254.692	0.000

Total Area of Peak = 1163941.310 [μV.Sec]

% purity = 98.4%

Compound 20

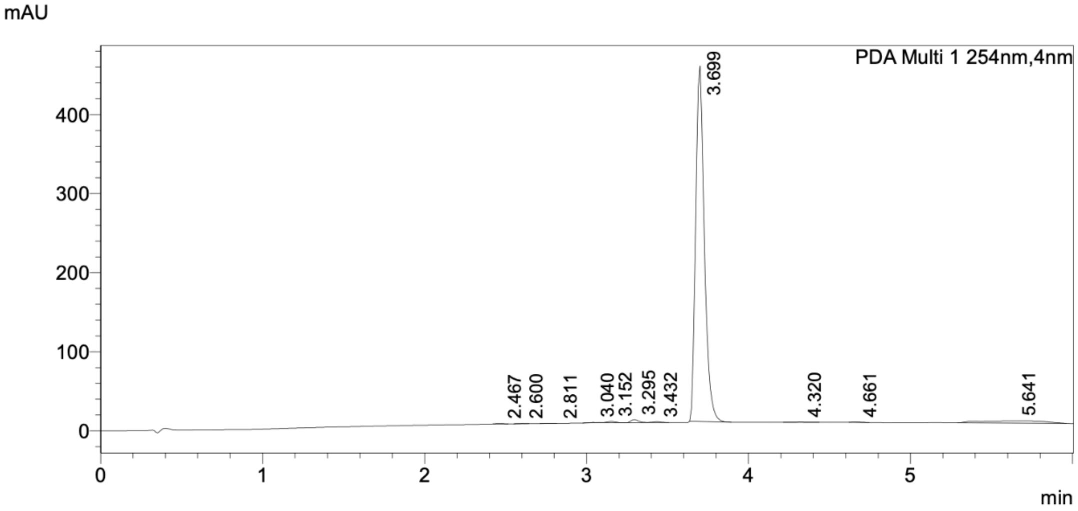

PDA Ch1 254nm

Peak#	Ret. Time	Area	Height	Area%
1	2.467	1253	385	0.070
2	2.600	956	299	0.053
3	2.811	663	12	0.037
4	3.040	1509	483	0.084
5	3.152	5847	1663	0.327
6	3.295	12454	3750	0.697
7	3.432	3170	1002	0.177
8	3.699	1678409	449496	93.924
9	4.320	1418	220	0.079
10	4.661	1461	400	0.082
11	5.641	79855	2667	4.469
Total		1786995	460378	100.000

Compound 21

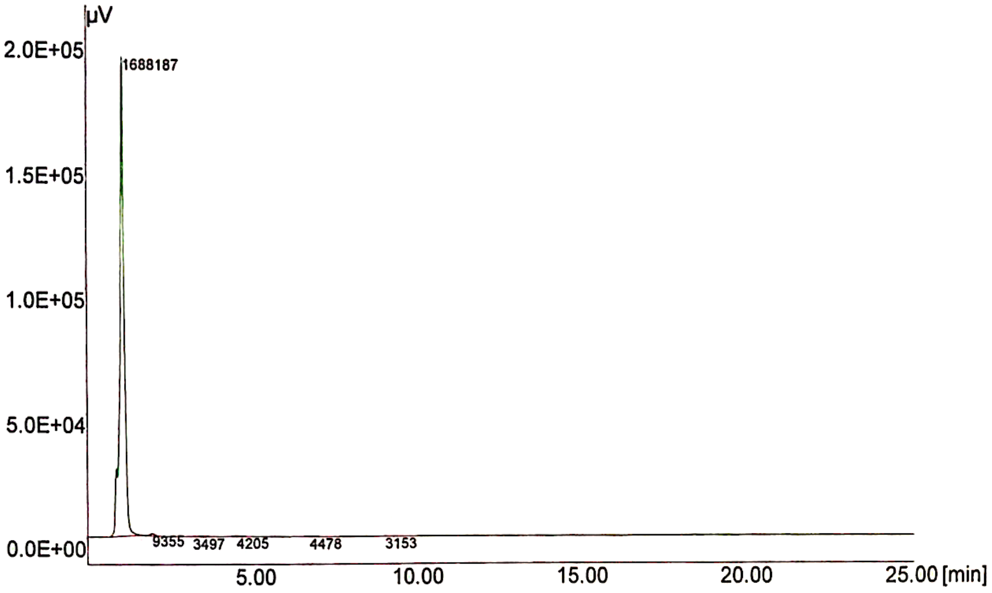

#	Name	RT	Area [μV.Sec]	Quantity
1		1.267	1688187.000	0.000
2		2.133	9354.532	0.000
3		3.358	3497.347	0.000
4		4.683	4205.231	0.000
5		6.892	4478.451	0.000
6		9.175	3153.472	0.000

Total Area of Peak = 1712876.033 [μV.Sec]

% purity = 98.5%
